# Screening of *Lactiplantibacillus plantarum* 67 with Strong Adhesion to Caco-2 Cells and the Effects of Protective Agents on Its Adhesion Ability during Vacuum Freeze Drying

**DOI:** 10.3390/foods12193604

**Published:** 2023-09-28

**Authors:** Dawei Chen, Congcong Guo, Chenyu Ren, Zihan Xia, Haiyan Xu, Hengxian Qu, Yunchao Wa, Chengran Guan, Chenchen Zhang, Jianya Qian, Ruixia Gu

**Affiliations:** 1 College of Food Science and Engineering, Yangzhou University, Yangzhou 225127, China; chendawei0816@163.com (D.C.);; 2 Jiangsu Key Laboratory of Dairy Biotechnology and Safety Control, Yangzhou 225127, China; 3 Jiangsu Yuhang Food Technology Co., Ltd., Yancheng 224000, China

**Keywords:** vacuum freeze-drying, protective agents, *Lactiplantibacillus plantarum* 67, adhesion

## Abstract

Adhesion to the intestinal tract provides the foundation for Lactobacillus to exert its benefits. Vacuum freeze-drying (VFD) is currently one of the main processing methods for Lactobacillus products. Therefore, the effects of VFD on the adhesion and survival of *Lactiplantibacillus plantarum* 67 were investigated in this study. The results show that *L. plantarum* 67 exhibits remarkable tolerance following successive exposure to simulated saliva, gastric juice and intestinal juice, and also has a strong adhesion ability to Caco-2 cells. The adhesion and survival rates of *L. plantarum* 67 significantly decreased after VFD in phosphate-buffered saline (PBS), whereas they significantly increased in protective agents (PAs) (*p* < 0.05). Scanning electron microscope observations show that *L. plantarum* 67 aggregated more to Caco-2 cells in PAs than in PBS, and its shape and size were protected. Proteomics detection findings indicated that differentially expressed proteins (DEPs) related to adhesins and vitality and their pathways in *L. plantarum* 67 were significantly affected by VFD (*p* < 0.05). However, the expression of DEPs (such as cold shock protein, cell surface protein, adherence protein, chitin-binding domain and extracellular transglycosylase, membrane-bound protein) was improved by PAs. Compared with PBS, the PAs significantly adjusted the phosphotransferase system and amino sugar and nucleotide sugar metabolism pathways (*p* < 0.05). VFD decreased the adhesion and vitality of *L. plantarum* 67, while the PAs could exert protective effects by regulating proteins and pathways related to adhesion and vitality.

## 1. Introduction

Adhesion is a fundamental property for bacterial survival in the intestinal tract of the body. This property can help bacteria to colonize the intestinal tract, increase communication between bacteria and the gastrointestinal tract and promote probiotic functions. Therefore, a crucial aspect of the function of probiotics in vivo is the attachment of lactic acid bacteria (LAB) to intestinal epithelial cells (IECs) [1]. LAB can compete with intestinal pathogens for adhesion sites at the mucosal interface through competition, inhibition and repulsion, and the beneficial substances from its metabolism can also inhibit the invasion and colonization of pathogens [2]. It is also found that LAB can regulate immunity in vivo by activating macrophages and enhancing the activity of natural killer cells and immunoglobulin secretion [3].

The specific binding of proteins, polysaccharides, lipoteichoic acid and other adhesins on the surface of LAB to adhesion receptors in IECs or the intestinal mucus layer is essential for their adhesion [4]. Moonlighting proteins are important proteins with multiple functions, and individual polypeptide chains have two or more physiological functions, such as protein synthesis, nucleic acid stabilization, glycolysis and chaperone activity [5]. Among the LAB cell surface moonlighting proteins, there are over 100 cytoplasmic proteins with a second function, which typically bind to host cells and tissues as adhesion proteins [6]. These proteins include 30S and 50S ribosomal proteins, elongation factors (EF-Tu, EF-P and EF-G), enolase (Eno), DnaK, cold shock proteins (Csps), small heat shock proteins (sHsps) and chaperonin GroEL [7].

Due to the increasing identification of probiotic functions of LAB and growing public health awareness, the production and application technology of LAB has become a research hotspot nowadays. Vacuum freeze-drying (VFD) has been widely used to produce starters for many fermented foods and probiotic solid beverages because of its high probiotic survival rate, long food storage time and convenient application.

However, ice crystals formed during VFD can damage cell membrane permeability and metabolism, resulting in changes in intracellular solute concentration and mechanical damage. This can affect the DNA stability and survival of LAB and damage adhesion [8]. It has been increasingly shown that adding protective agents (PAs; such as skim milk, sugars and sodium glutamate) can protect LAB and improve its survival [9,10]. But, the effects of PAs on the adhesion ability during VFD remain unclear. Therefore, LAB with strong resistance to oro-gastrointestinal transit (OGT) stress and strong adhesion to intestinal epithelial Caco-2 cells were selected, and the effects of PAs on the adhesion, survival and the surface morphology of LAB adhesion to Caco-2 cells after VFD were studied. In addition, proteomics was used to explore the possible mechanism of the protective effect of the PAs on the adhesion and survival of LAB. This work can serve as a guide for enhancing the functional properties of probiotics and facilitating their use in probiotic production.

## 2. Materials and Methods

### 2.1. Strains of Bacteria

*Lactiplantibacillus plantarum* strains (67, L160, L167, K118, L169), *Lacticaseibacillus rhamnosus* strains (L13, O62, O165, L63, L115, L36, H113, e15), *Limosilactobacillus fermentum* strains (L7, L73, O45, L120, K112, K161, R412), *Streptococcus thermophilus* strains (F164, Y17, e114, K413, K173 and M613) used in this study were provided by the Jiangsu Key Laboratory of Dairy Biotechnology and Safety Control of China. These strains were isolated from traditional fermented foods and feces of Bama Longevity County, Guangxi Province, China. They were grown till the stationary phase in de Man Rogosa and Sharpe (MRS) broth (Hope Bio-Technology Co., Ltd., Qingdao, China) at 37 °C.

### 2.2. Oro-Gastrointestinal Transit Stress Tolerance of LAB

LAB was centrifuged at 3000× *g* for 20 min at 4 °C after being grown in MRS to stationary phase. The simulated OGT stress conditions (artificial saliva, gastric juice and intestinal juice) and test methods in this section referred to our previous study [11].

### 2.3. Adhesion of LAB to Caco-2 Cells

The Caco-2 cells (Procell CL-0050; Procell Life Science & Technology Co., Ltd., Wuhan, China) were incubated until a stable monolayer developed. The adhesion capacity of LAB to Caco-2 cells was assessed following the Guo et al. [12] approach.

### 2.4. Key Adhesin of LAB

The LAB suspension was centrifuged and washed with phosphate-buffered saline (PBS) after being grown in MRS. The lipoteichoic acids, extracellular polysaccharide (EPS) and surface proteins on the surface of LAB were removed, respectively, according to the methods in the reference [13]. The adhesion capacity of LAB to Caco-2 cells was tested after removing the adhesins, and the adhesion rate considerably decreased, showing that the removed adhesin was the key adhesin [13].

### 2.5. Vacuum Freeze-Drying of LAB

The collected LAB cells were washed and resuspended with sterile PBS (1 × 10^8^ CFU/mL). Then, the centrifugal mud (1 × 10^8^ CFU/g) obtained from PBS suspension was mixed with PAs (containing 16% skim milk, 10% inulin, 4% sucrose, 7% sodium glutamate and 12% trehalose) and PBS (136.89 mmol/L NaCl, 2.67 mmmol/L KCl, 8.10 mmmol/L Na_2_HPO_4_, 1.76 mmmol/L KH_2_PO_4_) at a ratio of 1:1 (*w*/*w*), respectively, and was dispensed into a penicillin bottle (5 mL). Next, the mixture was vacuum freeze-dried following Chen et al. [14]. The viable count of LAB was assessed, and the survival rate was calculated using the following formula:

(1) $$\mathrm{Survival rate}\;(\%)=\frac{\mathrm{CFU}/g\;\mathrm{after}\;\mathrm{VFD}}{\mathrm{CFU}/g\;\mathrm{before}\;\mathrm{VFD}}\times 100\%$$

### 2.6. Scanning Electron Microscopy

The coculture of LAB and Caco-2 cells was centrifuged at 8000× *g* for 10 min at 4 °C, and then the cells were resuspended in 2.5% (*w*/*v*) glutaraldehyde phosphate buffer (Solarbio Technology Co., Ltd., Beijing, China) to fix the cells. The fixed cells were dehydrated with different grades of ethanol [15]. After sputter-coating, the coculture cells were observed following the reference method [16].

### 2.7. Protein Extraction

The treatments were arranged as follows: C group (LAB without VFD; n = 3), N group (LAB with VFD in the PBS; n = 3) and P group (LAB with VFD in the PAs; n = 3). LAB with different treatments was centrifuged and then washed three times by PBS (pH 7.2) for protein extraction following Yang et al. [17]. The BCA Protein Assay Kit (Thermo Fisher Scientific Inc., Waltham, MA, USA) was used to measure the concentration of the protein.

Sodium dodecyl sulfate polyacrylamide gel electrophoresis (Figure S1) was used to detect the quality of extracted protein according to Fan et al. [18]. Briefly, the extracted proteins were mixed with 5× loading buffer and boiled for 5 min. The mixtures were loaded on 5% gel for 30 min, and then run on a 12% separating gel until they reached the bottom. The separated gel was dyed and then washed with water; finally, it was scanned by Image Scanner (GE HealthCare Technologies Inc., Chicago, IL, USA).

### 2.8. Protein Digestion and Tandem Mass Tag Labeling

The methods of protein digestion and tandem mass tag (TMT) labeling of peptides were performed according to the reference [19]. In detail, the extracted protein was mixed with triethylammonium bicarbonate (TEAB, Sigma, Roedermark, Germany; final concentration 100 mmol/L) and tris (2-carboxyethyl) phosphine (final concentration 10 mmol/L), which were incubated at 37 °C for 60 min. And then, iodoacetamide was added (final concentration 40 mmol/L), and reacted at 25 °C without light for 40 min. Next, precooled acetone (acetone: sample volume = 6:1) was added and centrifuged at 10,000× *g* for 20 min after being precipitated at −20 °C for 4 h. Precipitate was mixed with TEAB and trypsin for digestion overnight at 37 °C.

The TMT reagent (No. A44522, Thermo Fisher Scientific Inc., Waltham, MA, USA) was added to the peptide samples and incubated at 25 °C for 2 h. Then, hydroxylamine was added and was incubated at 25 °C for 30 min. At last, the products were combined in a tube and a vacuum concentrator was used to drain them.

### 2.9. Mass Spectrometry Identification

The peptide samples were redissolved with ultra-performance liquid chromatography loading buffer (2% acetonitrile, pH 10.0), and were loaded onto an ACQUITY UPLC BEH C18 column (1.7 μm, 2.1 mm × 150 mm, Waters, Milford, CT, USA), and separated with phase A (2% acetonitrile, pH 10) and phase B (80% acetonitrile, pH 10) at a flow rate of 200 μL/min over 48 min. The gradient was as follows: 0–1.9 min, 100% A; 1.9–2 min, 0–5% B; 2–17 min, 5–5% B; 17–18 min, 5–10% B; 18–35.5 min, 10–30% B; 35.5–38 min, 30–36% B; 38–39 min, 36–42% B; 39–40 min, 42–100% B; 40–44 min, 100%–100% B; 44–45 min, 100%–0 B; 45–48 min, 100% A. Peptides were combined into 14 fractions and were concentrated.

The above peptides were dissolved with mass spectrometry buffer for the second two-dimensional analysis and were loaded onto a C18 column (150 μm × 15 cm, Evosep, Odense, Denmark) for liquid phase separation in buffer A (0.1% formic acid) and buffer B (100% acetonitrile with 0.1% formic acid) for 44 min at a flow rate of 300 nL/min. The gradient was as follows: 0–2 min, 5–5% B; 2–30 min, 5–38% B; 30–40 min, 38–90% B; 40–44 min, 90% B. The full mass spectrometer used in this study has a resolution of 60 K, a mass range of 350–1500 m/z and a maximum injection time of 25 ms. MS/MS analysis was performed at a resolution of 15 K, and dynamic exclusion was 30 s.

### 2.10. Protein Identification and Quantification and Bioinformatics

The raw data were searched, identified and quantified following Wen et al. [20]. The database used in this study was uniprot-proteome_UP000000432_unique.fasta. Based on a false discovery rate (FDR) of less than 1%, a 95% confidence level for protein identification was applied to all data. To facilitate protein identification, a minimum of one distinct peptide identification was used. Subsequently, the differentially expressed proteins (DEPs) with thresholds of fold change (FC) > 1.5 or FC < 0.67 (*p* < 0.05) were identified as significantly regulated proteins. The function of each protein was identified by the Gene Ontology (GO) terms and classified by the GO enrichment analysis approach (http://beta.geneontology.org/ accessed on 22 March 2022). The functional subclasses and metabolic pathways of DEPs were identified using the Kyoto Encyclopedia of Genes and Genomes (KEGG; http://www.kegg.jp/kegg/pathway accessed on 28 March 2022) pathway enrichment analysis.

### 2.11. Statistical Analysis

SPSS Statistics 20 (International Business Machines Corporation, Armonk, NY, USA) was used to analyze the data, and the mean and standard deviation were used to express values. The differences were considered significant at *p* < 0.05. All graphs were plotted using Origin 2018 (OriginLab Corporation, Northampton, MA, USA) except for proteomic-related graphs. The proteomic data were analyzed on the Majorbio Cloud Platform (www.majorbio.com accessed on 17 March 2022).

## 3. Results

### 3.1. Survival of LAB after Simulated OGT Stress

Table 1 shows that OGT stress at different stages had different influences on the viability of LAB. The viability of LAB was slightly affected by simulated saliva alone, with which the survival rates were all more than 18%. In contrast, the survival rates were both significantly lower after sequential exposure to simulated saliva + gastric juice and simulated saliva + gastric juice + intestinal juice (*p* < 0.05). Only *L. plantarum* (67, L160, L167) and *L. fermentum* (L7 and L73) had a survival rate greater than 18% after simulated OGT stress in turn. We also found that *S. thermophilus* spp. in this study had a lower survival rate than other strains after exposure to simulated OGT stress in turn (*p* < 0.05).

Significant differences in the survival rates of strains are indicated by different lowercase letters in the same column (*p* < 0.05). The survival rate of the same strain is denoted by different capital letters in the same row when there are significant differences (*p* < 0.05). The results are presented as the mean ± SD (n = 3).

### 3.2. Adhesion to Caco-2 Cells

**Figure 1:**
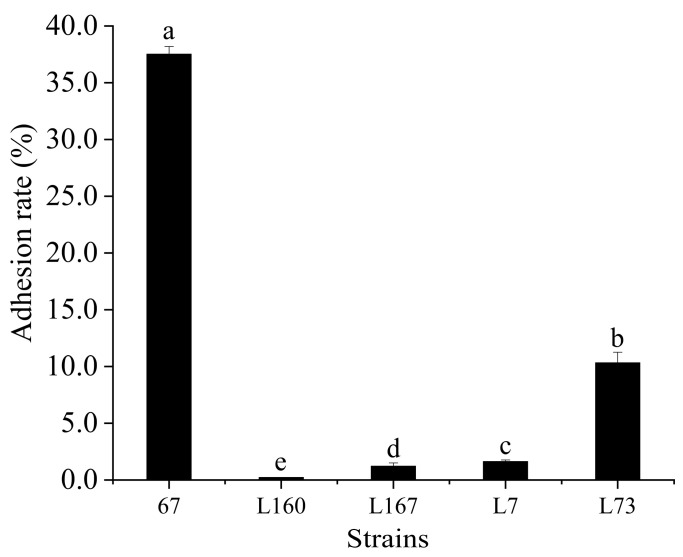
The adhesion rate of LAB to Caco-2 cells (n = 3, x ± SD). Significant differences in adhesion rate to Caco-2 cells are indicated by different letters (*p* < 0.05).

The adhesion ability of LAB with strong resistance to OGT stress was tested with the Caco-2 cells. *L. plantarum* 67 had a substantially greater adhesion rate to Caco-2 cells (37.55%) than other strains (*p* < 0.05; Figure 1).

### 3.3. Key Adhesin and Survival Rate of L. plantarum 67

**Figure 2:**
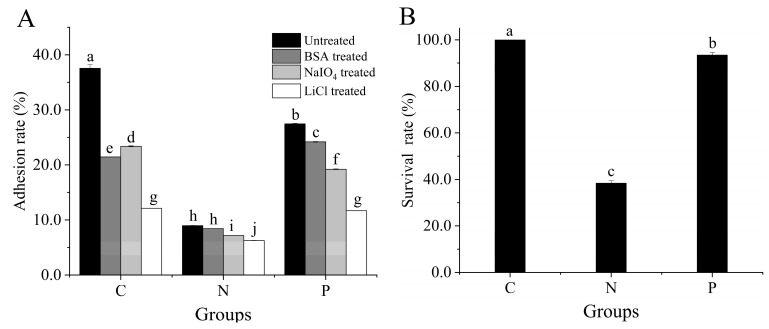
Key adhesin and survival rate of *L. plantarum* 67 before and after VFD: (**A**) the key adhesin and (**B**) the survival rate of *L. plantarum* 67. C group (*L. plantarum* 67 without VFD), N group (*L. plantarum* 67 with VFD in PBS) and P group (*L. plantarum* 67 with VFD in PAs). Different letters indicate significant differences in the adhesion rate to Caco-2 cells and the survival rate (*p* < 0.05).

Figure 2 shows that VFD significantly affected the adhesion and viability of *L. plantarum* 67 to Caco-2 cells. The adhesion rate and survival rate of *L. plantarum* 67 significantly decreased after VFD in the PBS (*p* < 0.05) and significantly increased in the PAs (*p* < 0.05). Therefore, the adverse effects of VFD on *L. plantarum* 67 adhesion and viability could be mitigated by PAs.

The adhesion rate of *L. plantarum* 67 significantly decreased under LiCl treatment, compared with those under control (without treatment), and BSA and NaIO_4_ treatments before VFD (*p* < 0.05; Figure 2A). This indicates that surface proteins were the key adhesins of *L. plantarum* 67. The adhesion rate showed the same changing trend after *L. plantarum* 67 was vacuum freeze-dried in the PBS and the PAs, respectively (*p* < 0.05; Figure 2A). This indicates that surface proteins were also the key adhesin after VFD (*p* < 0.05; Figure 2A). Hence, it is suggested that VFD did not alter the key adhesin of *L. plantarum* 67.

### 3.4. SEM Observation of L. plantarum 67 Adhering to Caco-2 Cells

**Figure 3:**
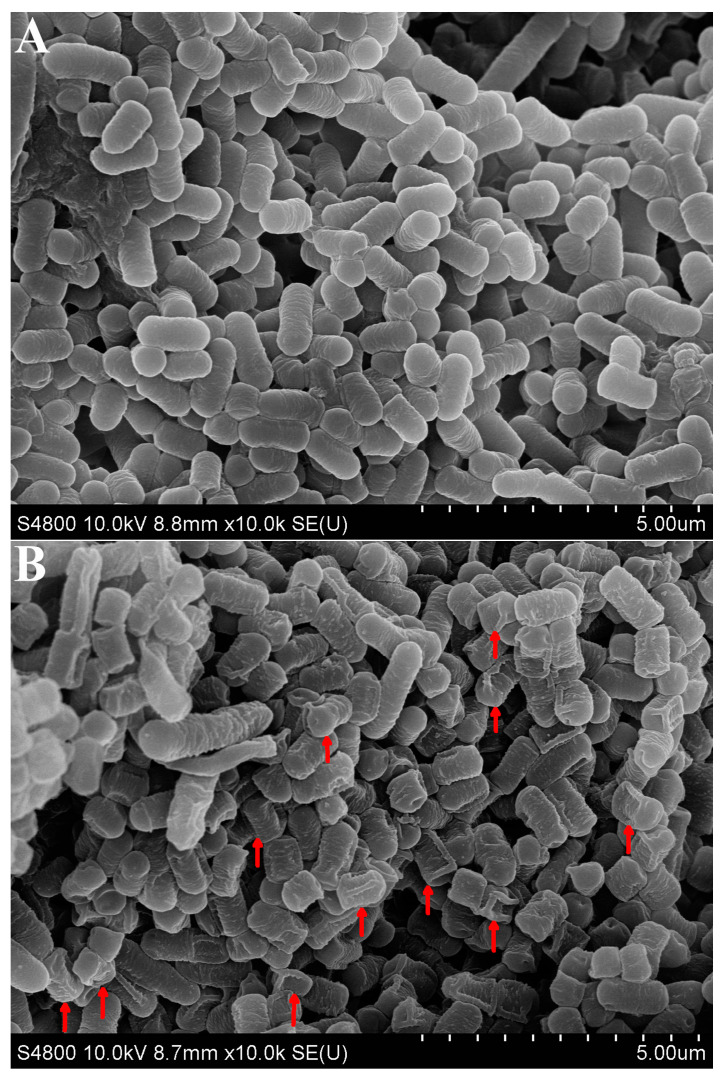

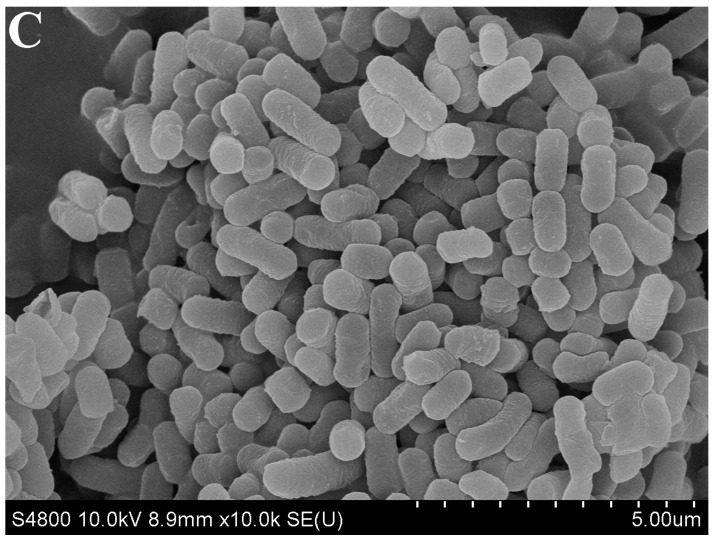
Surface micromorphology of *L. plantarum* 67 adhering to Caco-2 before and after VFD. (**A**) *L. plantarum* 67 without VFD; (**B**) *L. plantarum* 67 with VFD in PBS, red arrow indicates a significant change in the morphology and size of *L. plantarum* 67 cells; (**C**) *L. plantarum* 67 with VFD in PAs.

*L. plantarum* 67 without VFD adhered closely to Caco-2 cells, and the *L. plantarum* 67 cells were rod-shaped with obtusely rounded ends and had a smooth surface, intact morphology and uniform cell size (Figure 3A). After VFD in the PBS, *L. plantarum* 67 adhered loosely to the Caco-2 cells, and the cells were collapsed cell folds (red arrows) without uniform cell size (Figure 3B). However, after VFD in the PAs, *L. plantarum* 67 showed higher aggregation and adhesion ability to Caco-2 cells than that in the PBS, and the cells’ shapes and sizes showed little difference from those without VFD (Figure 3C). Thus, VFD damaged the adhesion and shape of *L. plantarum* 67, which could be improved by PAs.

### 3.5. Number of DEPs in L. plantarum 67 after Vacuum Freeze-Drying

**Figure 4:**
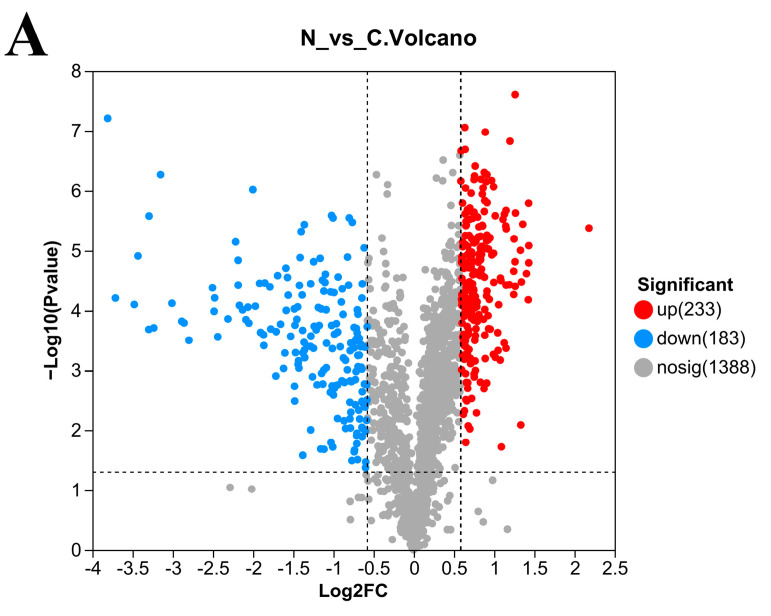

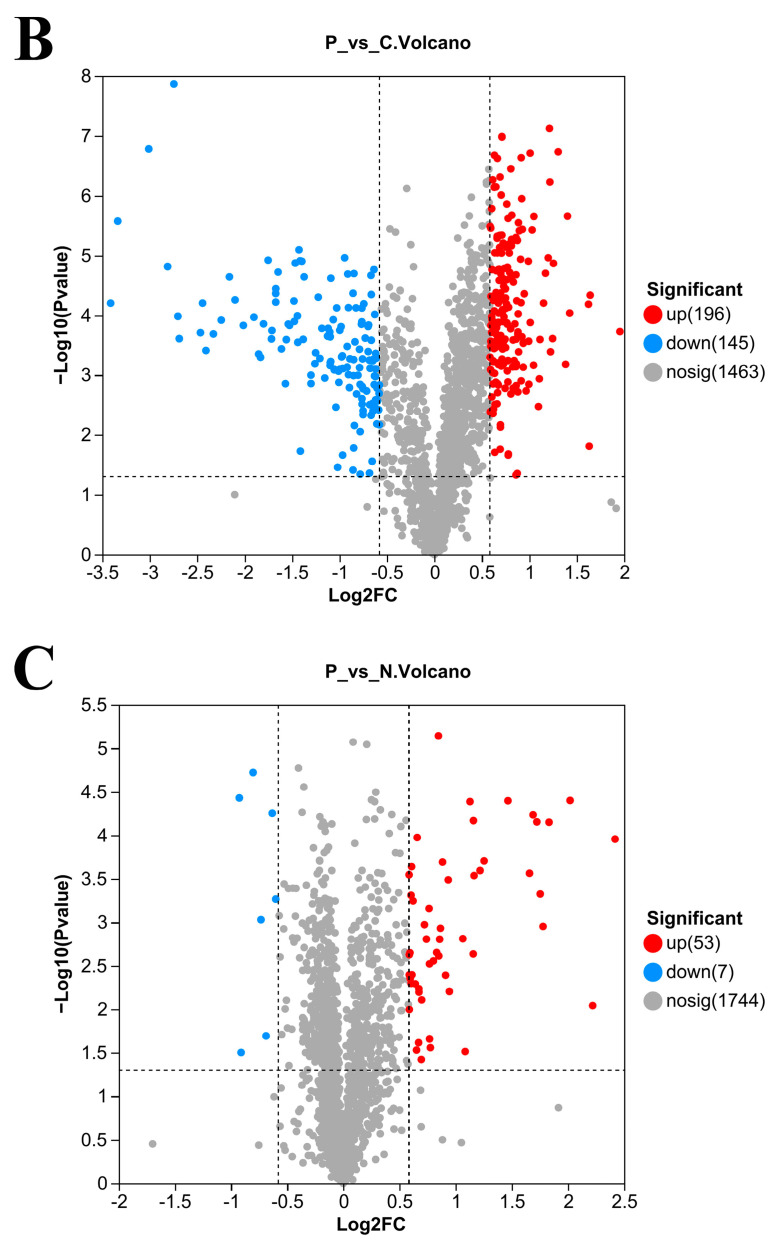
Volcano plot of DEPs in *L. plantarum* 67 before and after VFD. (**A**) N/C group; (**B**) P/C group; (**C**) P/N group. A particular protein is represented by each point on the graph; the points on the left are the proteins that are downregulated and the points on the right are the proteins that are upregulated.

A total of 416 proteins were significantly differentially expressed in the N/C group (*p* < 0.05; Figure 4A), with 233 upregulated DEPs and 183 downregulated DEPs (*p* < 0.05; Figure 4A). A total of 341 proteins were significantly differentially expressed in the P/C group (*p* < 0.05; Figure 4B), with 196 upregulated DEPs and 145 downregulated DEPs. When compared with the PAs, 53 proteins were upregulated, and 7 proteins were downregulated after *L. plantarum* 67 was vacuum freeze-dried in the PBS (*p* < 0.05; Figure 4C).

Table 2 presents the DEPs, mainly including some chaperone proteins, moonlighting proteins and cell surface proteins, and so on.

### 3.6. Gene Ontology (GO) Enrichment Analysis

**Figure 5:**
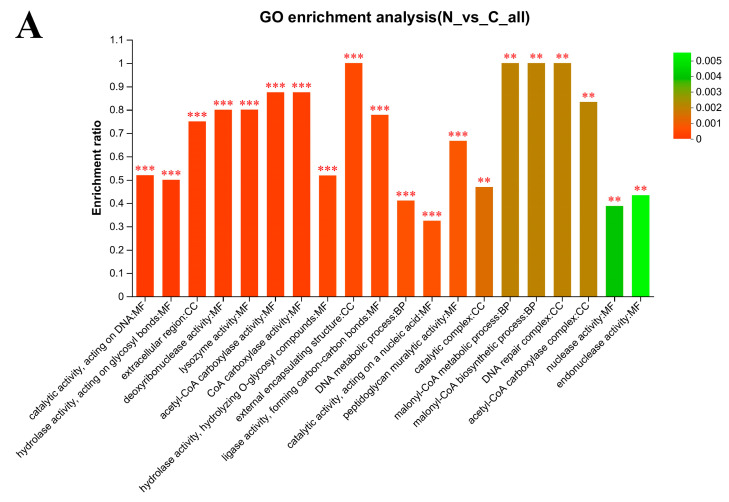

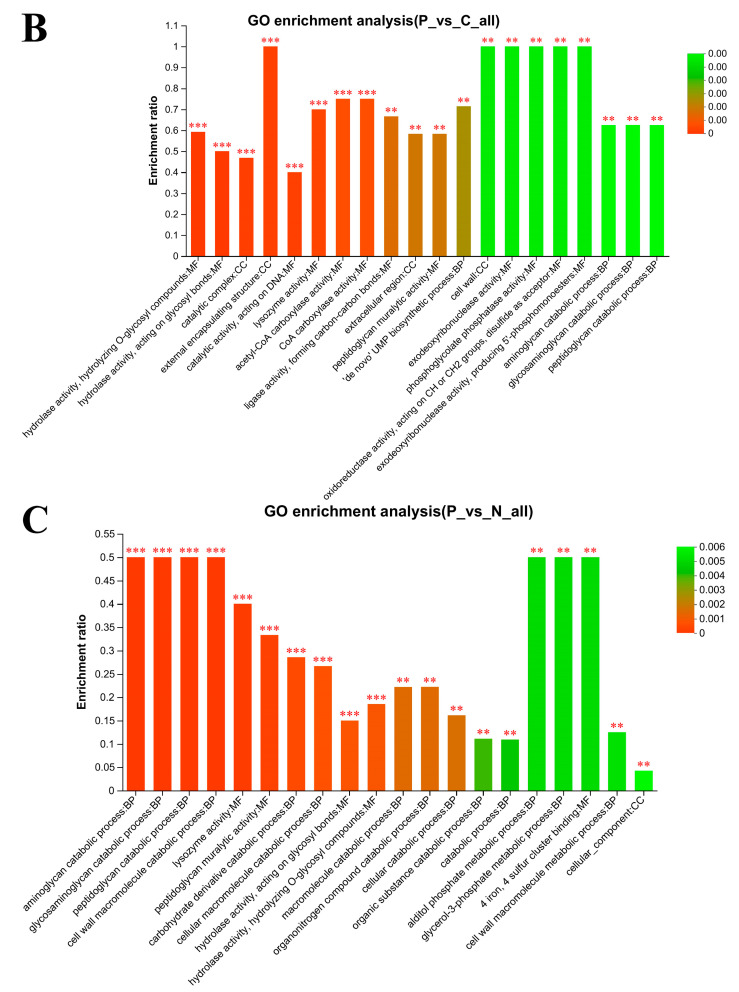
GO enrichment analysis of DEPs in *L. plantarum* 67 (top 20). (**A**) N/C group; (**B**) P/C group; (**C**) P/N group. Column color gradients indicate the significance of enrichment, where P or FDR less than 0.001 is marked as ***, P or FDR less than 0.01 is marked as **.

The GO functional analysis shows that the Biological Process (BP) categories (such as DNA metabolic processes, malonyl-CoA biosynthetic process and malonyl-CoA metabolic process) were extremely significantly enriched after *L. plantarum* 67 was vacuum freeze-dried in the PBS, compared with those without VFD (*p* < 0.01; Figure 5A), while aminoglycan catabolic process, “de novo” UMP biosynthetic process, glycosaminoglycan catabolic process, and peptidoglycan catabolic process were extremely significantly enriched in the PAs, compared with those without VFD (*p* < 0.01; Figure 5B).

The catalytic complex, external encapsulating structure and extracellular region in the Cellular Component (CC) categories were extremely significantly enriched after *L. plantarum* 67 was vacuum freeze-dried in the PBS and the PAs, compared with those without VFD (*p* < 0.01; Figure 5A,B).

The acetyl-CoA carboxylase activity, catalytic activity, acting on DNA, CoA carboxylase activity, hydrolase activity, acting on glycosyl bonds, hydrolase activity, hydrolyzing O-glycosyl compounds, ligase activity, forming carbon–carbon bonds, lysozyme activity and peptidoglycan muralytic activity in the Molecular Function (MF) categories were extremely significantly enriched after *L. plantarum* 67 was vacuum freeze-dried in the PBS and the PAs, compared with those without VFD (*p* < 0.01; Figure 5A,B). These categories contained a large number of DEPs. These results may suggest that VFD had a great influence on the molecular function of *L. plantarum* 67 cells.

In addition, the lysozyme activity, peptidoglycan muralytic activity, hydrolase activity, acting on glycosyl bonds and hydrolase activity, hydrolyzing O-glycosyl compounds in the MF categories were extremely significantly enriched after *L. plantarum* 67 was vacuum freeze-dried in the PAs when compared with those in the PBS (*p* < 0.01; Figure 5C), and they were also extremely significantly enriched in the N/C and P/C group. At the same time, it was found that the BP categories accounted for 15 among the top 20 most significant categories (*p* < 0.01; Figure 5C), and contained a large number of DEPs, which may indicate that PAs had a significant influence on the function of the biological process occurring when *L. plantarum* 67 was vacuum freeze-dried when compared with PBS.

### 3.7. KEGG Enrichment Analysis

**Figure 6:**
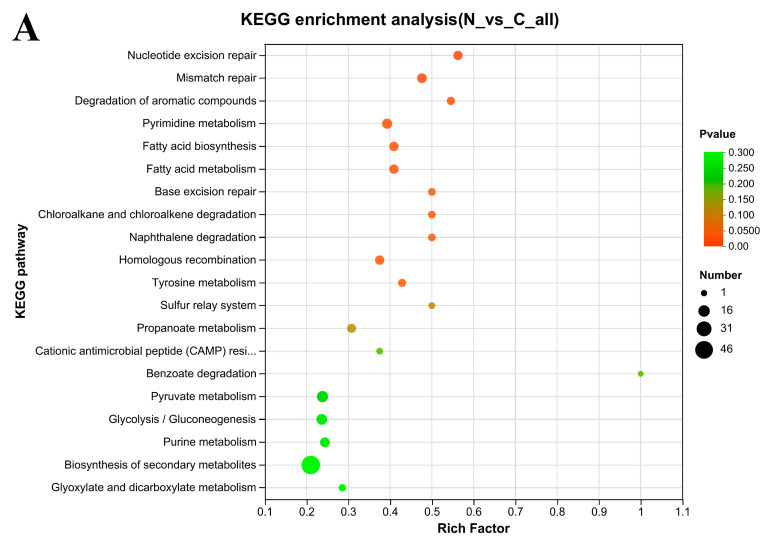

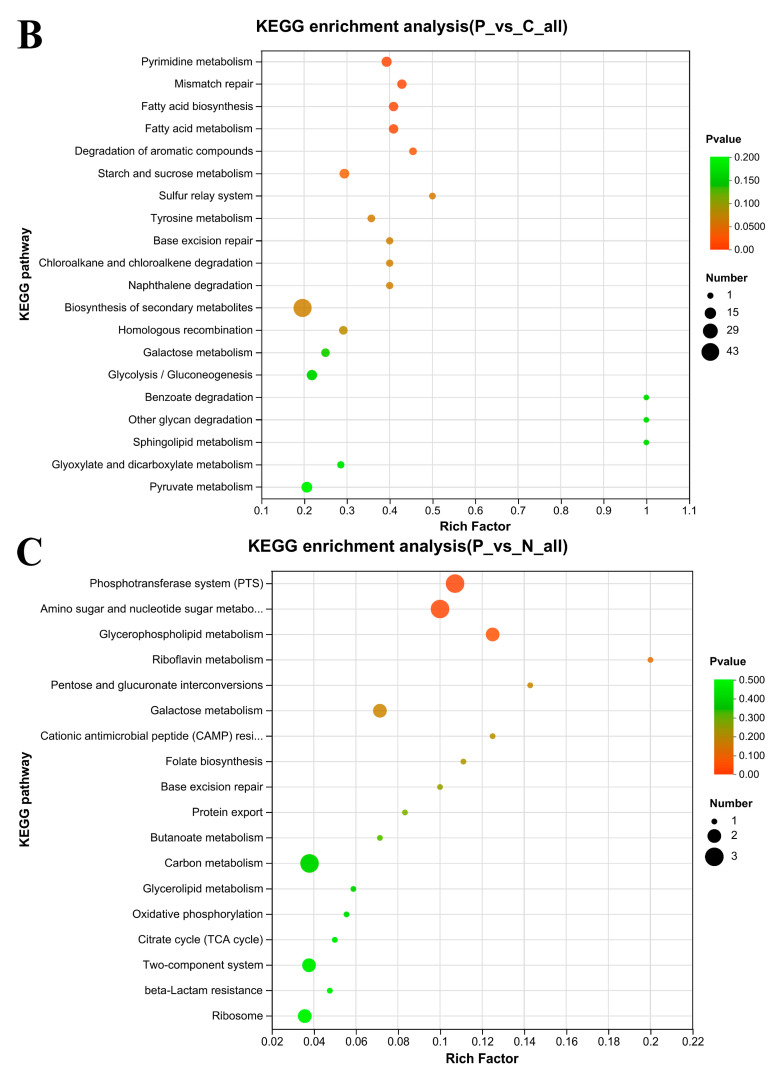
KEGG pathway enrichment scatter plot of DEPs in *L. plantarum* 67 (top 20). (**A**) N/C group; (**B**) P/C group; (**C**) P/N group. A KEGG pathway is represented by each bubble. The bubble size was the number of proteins enriched by the KEGG pathway. The bubbles’ colors correspond to the *p* value. The *p* value represents whether the enriched results are statistically significant, and the smaller the *p* value, the more statistically significant it is; generally, a *p* value less than 0.05 is considered a significant enrichment item for this function.

The DEPs were further subjected to KEGG pathway analysis. After *L. plantarum* S7 was vacuum freeze-dried in the PBS, the associations of DEPs with the pathways were as follows (Figure 6A): 9 DEPs, nucleotide excision repair (map03420, *p* = 0.0010); 10 DEPs, mismatch repair (map03430, *p* = 0.0029); 6 DEPs, degradation of aromatic compounds (map01220, *p* = 0.0096); 11 DEPs, pyrimidine metabolism (map00240, *p* = 0.0109); 9 DEPs, fatty acid biosynthesis (map00061, *p* = 0.0156); 9 DEPs, fatty acid metabolism (map01212, *p* = 0.0156); 5 DEPs, chloroalkane and chloroalkene degradation (map00625, *p* = 0.0283); 5 DEPs, naphthalene degradation (map00626, *p* = 0.0283); 5 DEPs, base excision repair (map03410, P=0.0283); 9 DEPs, homologous recombination (map03440, *p* = 0.0287); 6 DEPs, tyrosine metabolism (map00350, *p* = 0.0372).

After *L. plantarum* 67 was vacuum freeze-dried in the PAs, the associations of DEPs with the pathways were as follows (Figure 6B): 11 DEPs, pyrimidine metabolism (*p* = 0.0022); 9 DEPs, mismatch repair (*p* = 0.0028); 9 DEPs, fatty acid biosynthesis (*p* = 0.0041); 9 DEPs, fatty acid metabolism (*p* = 0.0041); 5 DEPs, degradation of aromatic compounds (*p* = 0.0196); 10 DEPs, starch and sucrose metabolism (map00500, *p* = 0.0323).

A total of 3 DEPs were associated with the phosphotransferase system (PTS) (map02060, *p* = 0.0387) and 3 DEPs were associated with amino sugar and nucleotide sugar metabolism (map00520, *p* = 0.0462) after *L. plantarum* 67 was vacuum freeze-dried in the PAs, compared with those in the PBS (Figure 6C).

## 4. Discussion

After simulating saliva conditions, the survival rate of most LAB was greater than 40% (Table 2), indicating that the pH 7.0 saliva is a suitable environment for the survival of LAB [21]; however, the fact that the expression of the *hsp*1 gene of LAB was upregulated may be one of the reasons that the survival rate of some strains was higher than 100% (Table 2) [22]. After successive exposure to simulated saliva and gastric juice conditions, the survival rate of LAB was significantly lower than that when exposed to simulated saliva (Table 2; *p* < 0.05), which may suggest that a low pH environment had a significant impact on the growth of strains; but the activity of the proton transfer membrane ATPase and glutamic acid decarboxylase of LAB were aroused when subjected to gastric acid stress to keep the balance of intracellular and extracellular pH, thereby contributing to LAB maintaining a high survival rate (Table 2) [23]. After successive exposure to simulated saliva, gastric juice and intestinal juice conditions, the genes related to bile stress of LAB were encoded, which may also be conducive to some strains having a higher survival rate (Table 2) [24,25].

During VFD, the intracellular enzyme outflow caused by cell dehydration easily leads to the decline of cell vitality [26], and the cells of *L. plantarum* 67 may collapse and rupture (Figure 3B) with the increasing degree of dehydration. However, the collapsing and rupturing of *L. plantarum* 67 cells were improved (Figure 3C) by the added PAs due to the reduction in the permeability of the cell membrane and the formation rate of ice crystals, and improvements regarding changes in ionic strength caused by electrolyte concentration inside and outside the cells [27,28].

The calcium ions in skim milk can activate the mediated signaling pathway, promote specific binding between LAB and surface receptors of Caco-2 cells, and enhance the adhesion ability of LAB to Caco-2 cells [29]. Furthermore, sodium glutamate can maintain the adhesion ability of LAB by improving the activity and expression of surface proteins during VFD [30]. It is suggested that PAs can affect the adhesion of LAB by affecting their associated proteins and pathways. Therefore, the effects of PAs (16% skim milk, 10% inulin, 4% sucrose, 7% sodium glutamate, and 12% trehalose) on the adhesion ability of *L. plantarum* 67 during VFD according to proteins and pathways were further investigated by proteomics in this study.

### 4.1. Effect of Moonlight Proteins on L. plantarum 67 Adherence and Survival

After VFD in the PBS and the PAs, the expression of 50S ribosomal proteins (L7/L12, L27; Accession: Q88YW7, Q88WN3) of *L. plantarum* 67 was significantly downregulated, while the expressions of EF-G (Accession: Q88XY8, F9USR0) was significantly upregulated, compared with those without VFD (*p* < 0.05; Table 2). These changes can reduce cellular energy consumption so as to favor the survival of *L. plantarum* 67 in the adverse environment of VFD [31]. They can also promote the secretion of cytoplasmic proteins into the extracellular system, thus helping to maintain the adhesion of *L. plantarum* 67 (Figure 2A) [7].

Stress proteins and folding proteins are the main companion proteins in LAB, including cold shock protein (Csp; Accession: F9UMI2, P96349) and small heat shock proteins (sHsps; Accession: F9USV1, F9UTM5). SHsps can prevent damage caused by cold stress [32]. The expression of Csp 2 and sHsps in *L. plantarum* 67 was markedly reduced in the N/C and P/C groups (*p* < 0.05; Table 2), while the expression of Csp 2 was significantly upregulated in the P/N group (*p* < 0.05; Table 2). However, Csp could improve the transcription and translation efficiency of cellular RNA and maintain the normal physiological function of LAB when the activity of other proteins was inhibited in the low temperature environment [33]. These results suggest that the adhesion and vitality of *L. plantarum* 67 could be improved by PAs accordingly to regulate the expression of moonlight proteins (Figure 2).

### 4.2. Effect of Cell Surface Proteins and Other Proteins on the Adhesion and Survival of L. plantarum 67

Cell surface protein has important physiological functions, such as maintaining cell structure and shape, regulating LAB’s adhesion to IECs and participating in intercellular recognition [34]. MUB is an important adhesion protein on the surface of Lactobacillus [35], which has a characteristic secretory signal sequence and an LPXTG-anchoring motif at its N-terminus and C-terminus, respectively. MUB can realize its adhesion function by recognizing sialic acid residues of mucin chains [36,37]. In this study, the expression of cell surface proteins (CscA/DUF916 family, CscB family, LPXTG-motif cell wall anchor; Accession: F9ULM1, F9USA9, F9UU92, F9UNI8, F9UU91, F9UU93, F9UUC6, F9ULS6, F9UM21, F9US12, F9US24) and adherence protein, chitin-binding domain (Accession: F9UP60) and mucus-binding protein (MUB; Accession: F9UME2, F9UP14, F9UR18, F9USM7) in *L. plantarum* 67 were significantly downregulated in PBS after VFD (*p* < 0.05; Table 2), while being significantly upregulated in the PAs (*p* < 0.05; Table 2). Hence, the PAs could facilitate the synthesis of MUB and improve the expression of adhesion protein, which was conducive to maintaining the adhesion of *L. plantarum* 67 (Figure 2A).

The hydroxyl groups contained in trehalose and sucrose in the PAs can replace the lost water, form hydrogen bonds with the phosphate and protein polar groups in the LAB cell membrane, and wrap on the protein surface in the form of a “hydration membrane”, thereby protecting the integrity of the LAB cell membrane and protein structure and function during the VFD [38,39]; and at the same time, a glassy layer is formed on the surface of the LAB cell to wrap the biological structure, limiting the intramolecular mobility of the protein and protecting the function of the surface proteins of LAB [40]. In addition, sodium glutamate in the PAs can also form hydrogen bonds with the polar groups of LAB cell surface proteins so as to maintain the structural and functional integrity of LAB surface proteins [30]. Therefore, PAs may also maintain the adhesion ability of *L. plantarum* 67 by protecting the activity and function of surface proteins, adhesion proteins and mucus-binding proteins.

The expression of extracellular transglycosylase membrane-bound protein (Accession: F9USH2) and extracellular protein membrane-anchored (Accession: F9URS4), NlpC/P60 family, gamma-D-glutamate-meso-diaminopimelate muropeptidase (Accession: F9UQA0) in *L. plantarum* 67 were significantly downregulated after VFD in PBS (*p* < 0.05; Table 2) while being significantly upregulated in PAs (*p* < 0.05; Table 2). However, extracellular transglycosylase is an important protein for synthesizing LAB exopolysaccharides [41]. Extracellular proteins can promote LAB’s adherence to the intestinal tract [42]. Therefore, the PAs may contribute to repairing the damage to adhesion and vitality caused by VFD (Figure 2).

### 4.3. Some KEGG Pathways Enrichment Analysis

DNA damage repair pathways include (e.g.,) mismatch repair, homologous recombination, base excision repair, and tyrosine metabolism [43]. These pathways were subjected to significant differential changes in the N/C and P/C groups (*p* < 0.005; Figure 6). The expression of UvrABC system protein C (Accession: Q88VE9; Nucleotide excision repair pathway), DNA mismatch repair proteins MutL and MutS (Accession: Q88UZ8, Q88UZ7; Mismatch repair pathway), DNA polymerase III subunit alpha and DNA-directed DNA polymerase III delta chain (Accession: F9UQ72; Homologous recombination pathway) were significantly upregulated by VFD (*p* < 0.05; Table 2). In comparison to PAs, the expression of UvrABC system protein C in PBS was noticeably higher (*p* < 0.05; Table 2). The results show that PBS may cause more DNA damage than PAs, and the DNA damage repair pathways were employed by *L. plantarum* 67 to repair DNA lesions by VFD [44,45].

The DEPs of the PTS system, N-acetylglucosamine/galactosamine-specific EIIA component (Accession: F9URE0) and Putative PTS system EIIA component (Accession: F9UUF2) in *L. plantarum* 67 were all enriched in the phosphotransferase system (PTS) and amino sugar and nucleotide sugar metabolism pathways, which underwent significant differential changes in the P/N group (*p* < 0.05; Figure 6). However, the PTS system can not only regulate the carbon metabolism, but also improve their response to cold shock [46], and the Positive PTS system EIIA component may also be related to the transport of raffinose, which can promote the adhesion of Lactobacillus to intestine [47]. At the same time, we also found that galactokinase (Accession: Q88SE8) was enriched in the amino sugar and nucleoside sugar metabolism pathway, which can synthesize the UDP-galactose through galactose-1-phase, a precursor of EPS [48]. And the three proteins above were all significantly upregulated in P/N group (*p* < 0.05; Table 2), indicating that PAs may regulate the related proteins and pathways to protect the vitality and adhesion of *L. plantarum* 67 during VFD when compared with PBS.

In the cationic antimicrobial peptide resistance pathway (map01503), the DEPs of D-alanyl carrier protein 1 (Accession: Q88VM8) was enriched, which was critical carrier proteins for the synthesis of teichoic acid and lipoteichoic acid [49]. However, the expression of D-alanyl carrier protein 1 was significantly downregulated by VFD, while being significantly upregulated in PAs (*p* < 0.05; Table 2). The change indicates that the expression of protein and its pathway related to teichoic acid synthesis in *L. plantarum* 67 was improved, thus maintaining its adhesion by PAs.

In the peptidoglycan biosynthesis pathway (map00550), peptidoglycan was finally synthetized under the action of penicillin-binding protein (ClassA PBP), serine-type D-Ala-D-Ala carboxypeptidase (LMW PBP) and zinc D-Ala-D-Ala carboxypeptidase (VanY). It was found that the expression of LMW PBP (Accession: F9UN64) in *L. plantarum* 67 was significantly downregulated in the N/C group (*p* < 0.05; Table 2) while being significantly upregulated in the P/N group (*p* < 0.05; Table 2). Therefore, upregulating the expression of related proteins in the peptidoglycan pathway by the PAs can improve the adhesion of *L. plantarum* 67 [50].

The oligopeptide ABC transporters lipoprotein-binding protein (Accession: F9USS1) was significantly upregulated in the Quorum sensing pathway (map02024) in the P/N group (*p* < 0.05; Table 2). However, oligopeptide ABC transporters lipoprotein-binding protein is involved in biofilm formation [51,52], and could also promote Lactobacillus adhesion to host cells [53]. Therefore, the PAs can help to maintain the adhesion of *L. plantarum* 67 to host cells and enhance the vitality of *L. plantarum* 67 during VFD by promoting biofilm formation.

## 5. Conclusions

*L. plantarum* 67 exhibited strong resistance to OGT stress and strong adhesion to intestinal epithelial Caco-2 cells. The adhesion and vitality of *L. plantarum* 67 significantly decreased due to VFD, while adding PAs had a protective effect. Furthermore, the expressions of proteins related to adhesins (such as moonlight proteins, teichoic acid, exopolysaccharides and cell surface protein, and so on) and proteins related to survival (such as cold shock protein, sHsps, and so on) in *L. plantarum* 67 were regulated by the PAs after VFD. The pathways related to adhesion and survival were also adjusted by the PAs to resist the damaging effects on the adhesion and vitality of *L. plantarum* 67 caused by VFD.

## Figures and Tables

**Table 1:** The survival rate of LAB after exposure to simulated OGT stress (n = 3, x ± SD).

Strains		Survival Rate after Exposure in Simulated Saliva/(%)	Survival Rate after Exposure in Simulated Saliva and Gastric Juice/(%)	Survival Rate after Exposure in Simulated Saliva, Gastric Juice and Intestinal Juice/(%)
*L. plantarum*	67	97.19 ± 0.14 ^eA^	84.29 ± 0.02 ^bB^	83.23 ± 0.10 ^aC^
	L160	131.11 ± 0.42 ^aA^	83.75 ± 0.05 ^cB^	67.63 ± 0.11 ^bC^
	L167	86.82 ± 0.09 ^iA^	55.48 ± 0.11 ^hB^	18.59 ± 0.07 ^dC^
	K118	95.10 ± 0.58 ^gA^	29.02 ± 0.02 ^rB^	3.12 ± 0.00 ^nC^
	L169	75.18 ± 0.33 ^mA^	35.54 ± 0.06 ^mB^	2.81 ± 0.01 ^oC^
*L. rhamnosus*	L13	48.75 ± 0.32 ^tA^	34.59 ± 0.12 ^nB^	15.34 ± 0.04 ^fC^
	O62	42.87 ± 0.22 ^wA^	32.16 ± 0.11 ^oB^	9.98 ± 0.01 ^hC^
	O165	96.25 ± 0.11 ^fA^	30.92 ± 0.05 ^pB^	7.51 ± 0.01 ^iC^
	L63	79.91 ± 0.07 ^lA^	52.66 ± 0.09 ^iB^	7.34 ± 0.02 ^jC^
	L115	63.93 ± 0.34 ^qA^	29.18 ± 0.11 ^qB^	6.54 ± 0.01 ^kC^
	L36	87.59 ± 0.19 ^hA^	6.93 ± 0.02 ^wB^	1.97 ± 0.00 ^qC^
	H113	71.95 ± 0.01 ^oA^	1.44 ± 0.00 ^zB^	0.77 ± 0.00 ^tC^
	e15	97.91 ± 0.03 ^dA^	36.85 ± 0.17 ^lB^	0.36 ± 0.00 ^uC^
*L. fermentum*	L7	102.33 ± 0.49 ^cA^	60.24 ± 0.17 ^gB^	25.09 ± 0.06 ^cC^
	L73	84.65 ± 0.26 ^jA^	127.73 ± 0.32 ^aB^	18.26 ± 0.03 ^eC^
	O45	104.86 ± 0.44 ^bA^	71.99 ± 0.47 ^eB^	14.37 ± 0.03^gC^
	L120	84.05 ± 0.27 ^kA^	38.38 ± 0.06 ^kB^	l5.37 ± 0.00 ^lC^
	K112	49.29 ± 0.09 ^sA^	15.82 ± 0.06 ^tB^	3.44 ± 0.01 ^mC^
	K161	74.39 ± 0.21 ^nA^	7.35 ± 0.01 ^vB^	2.14 ± 0.00 ^pC^
	R412	46.66 ± 0.18 ^uA^	18.33 ± 0.07 ^sB^	1.25 ± 0.00 ^sC^
*S. thermophilus*	F164	66.41 ± 0.15 ^pA^	5.54 ± 0.01 ^xB^	1.26 ± 0.00 ^rC^
	Y17	45.76 ± 0.35 ^vA^	2.73 ± 0.01 ^yB^	0.16 ± 0.00 ^vC^
	e114	74.41 ± 0.11 ^nA^	48.24 ± 0.05 ^jB^	0.13 ± 0.00 ^wC^
	K413	63.09 ± 0.21 ^rA^	61.19 ± 0.75 ^fB^	0.12 ± 0.00 ^xC^
	K173	41.33 ± 0.40 ^xB^	77.19 ± 0.16 ^dA^	0.06 ± 0.00 ^yC^
	M613	20.80 ± 0.10 ^yA^	13.06 ± 0.09 ^uB^	0.04 ± 0.00 ^zC^

Significant differences in the survival rates of strains are indicated by different lowercase letters in the same column (*p* < 0.05). The survival rate of the same strain is denoted by different capital letters in the same row when there are significant differences (*p* < 0.05). The results are presented as the mean ± SD (n = 3).

**Table 2:** Some DEPs related to adhesion and survival in *L. plantarum* 67 before and after VFD.

Accession	N/C		P/C		P/N	
	FC	*p* Value	FC	*p* Value	FC	*p* Value
Q88WN3	0.67	0.0031	-	-	-	-
Q88YW7	-	-	0.62	0.0002	-	-
F9URT0	0.61	0.0002	-	-	-	-
F9UR61	0.52	0.0002	0.62	0.0006	-	-
F9UTV9	0.52	<0.0001	0.55	<0.0001	-	-
F9UTM2	0.46	<0.0001	0.66	0.0027	-	-
F9UP60	0.12	<0.0001	0.21	0.0001	1.70	0.0007
F9ULM1	0.46	0.0017	-	-	-	-
F9USA9	0.50	<0.0001	-	-	-	-
F9UU92	0.62	0.0006	0.65	0.0010	-	-
F9UNI8	0.41	0.0099	0.58	0.0457	-	-
F9UU91	0.22	<0.0001	0.31	<0.0001	-	-
F9UU93	0.14	0.0003	0.19	0.0004	-	-
F9UUC6	0.29	0.0002	0.64	0.0030	2.19	<0.0001
F9ULS6	0.55	0.0016	0.65	0.0036	-	-
F9UM21	0.47	0.0009	0.59	0.0016	-	-
F9US12	0.50	0.0026	0.66	0.0066	-	-
F9US24	0.18	0.0001	0.25	0.0001	-	-
F9URE0	0.10	0.0002	0.28	0.0005	2.76	<0.0001
F9UUF2	0.64	0.0033	-	-	2.23	<0.0001
P96349	0.58	0.0064	-	-	1.65	0.0011
F9UMI2	-	-	1.82	0.0015	1.81	0.0016
Q88VM8	0.66	0.0034	-	-	1.56	0.0051
F9UPA0	0.34	<0.0001	0.54	0.0005	1.59	0.0058
F9UQ72	1.78	<0.0001	1.71	<0.0001	-	-
Q88UZ8	2.11	0.0007	1.84	0.0019	-	-
Q88UZ7	1.96	<0.0001	1.82	<0.0001	-	-
F9USR0	1.51	<0.0001	-	-	-	-
Q88SE8	-	-	1.95	0.0018	1.78	0.0022
Q88XY8	1.50	<0.0001	1.50	<0.0001	-	-
F9URS4	0.18	0.0003	0.28	0.0004	1.50	0.0024
F9UQA0	0.14	0.0002	0.18	0.0002	-	-
F9UUA0	0.18	<0.0001	0.23	<0.0001	-	-
F9USH2	0.52	0.0064	-	-	1.57	0.0295
Q890I8	0.23	<0.0001	0.29	0.0001	-	-
F9USL7	0.41	0.0004	0.47	0.0006	-	-
F9UME2	0.30	0.0012	0.34	0.0014	-	-
F9UP14	0.11	<0.0001	0.12	<0.0001	-	-
F9UR18	0.60	0.0004	-	-	-	-
F9USM7	0.36	0.0005	0.47	0.0007	-	-
F9USS1	0.61	0.0167			1.50	0.0101
F9UN64	0.44	0.0002	0.55	0.0006	-	-
F9USV1	0.28	<0.0001	0.31	<0.0001	-	-
F9UTM5	0.38	<0.0001	0.36	<0.0001	-	-
Q88VE9	1.75	0.0001	1.45	0.0002	0.83	0.0044

The names of DEPs are shown in Table S1. “-” indicates that the proteins’ FCs were not greater than 1.2 or not less than 0.83, and there was not a significant difference (*p* > 0.05) in this group.

## Data Availability

The datasets generated during and/or analyzed during the current study are available from the corresponding author on reasonable request.

## Supplementary Materials

The following supporting information can be downloaded at: https://www.mdpi.com/article/10.3390/foods12193604/s1, Figure S1: SDS-PAGE electrophoresis map of *L. plantarum* 67 before and after vacuum freeze-drying. Table S1: DEPs in *L. plantarum* 67 before and after vacuum freeze-drying.
