# Supplementary material for: Screening of Lactiplantibacillus plantarum 67 with Strong Adhesion to Caco-2 Cells and the Effects of Protective Agents on Its Adhesion Ability during Vacuum Freeze Drying
Source: Foods. 2023 Sep 28;12(19):3604. doi: 10.3390/foods12193604 (PMC10572606; doi:10.3390/foods12193604)
Supplement: Supplementary file 1 [file foods-12-03604-s001.zip › foods-2583801-supplementary.pdf]

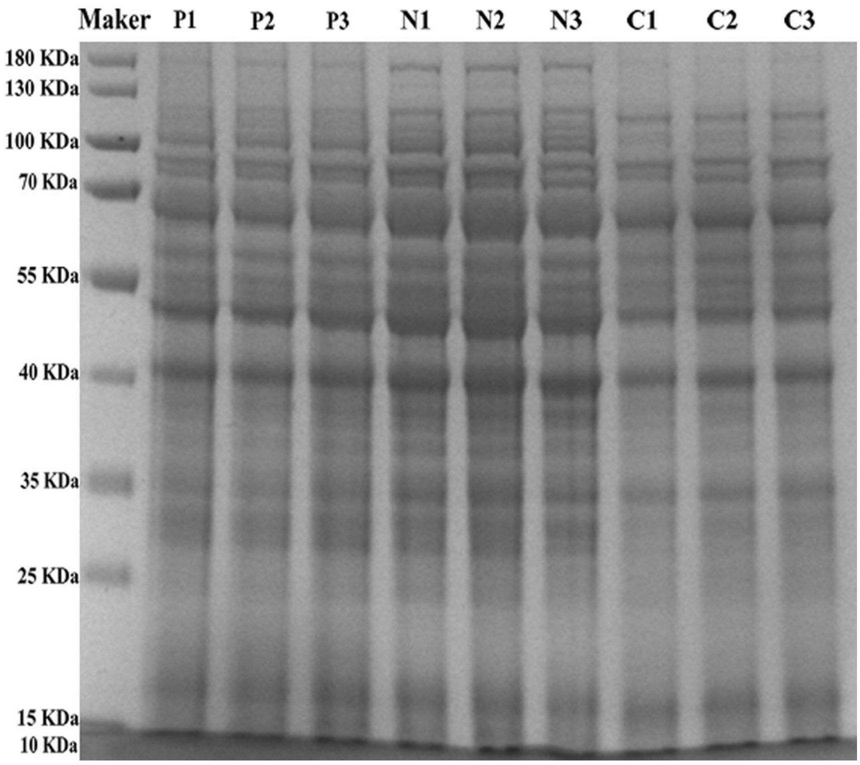

Figure S1. SDS-PAGE electrophoresis map of *L. plantarum* 67 before and after vacuum freeze drying

Table S1. DEPs in *L. plantarum* 67 before and after vacuum freeze drying

Group	Accession	Protein Name	Description	FC	P_ value	Regulate
N/C	F9UMQ5	ttdB	L(+)-tartrate dehydratase, subunit B OS=Lactobacillus plantarum (strain ATCC BAA-793 / NCIMB 8826 / WCFS1) OX=220668 GN=ttdB PE=3 SV=1	0.0762 18905	6.21E-0 5	down
	F9USC4	S9 family peptidase	Uncharacterized protein OS=Lactobacillus plantarum (strain ATCC BAA-793 / NCIMB 8826 / WCFS1) OX=220668 GN=lp_2994 PE=4 SV=1	0.1018 87403	2.65E-0 6	down
	F9UP14	lp_1643	Mucus-binding protein, LPXTG-motif cell wall anchor OS=Lactobacillus plantarum (strain ATCC BAA-793 / NCIMB 8826 / WCFS1) OX=220668 GN=lp_1643 PE=4 SV=1	0.1125 43184	5.37E-0 7	down
	F9USB9	zmp3	Extracellular zinc metalloproteinase, M10 family OS=Lactobacillus plantarum (strain ATCC BAA-793 / NCIMB 8826 / WCFS1) OX=220668 GN=zmp3 PE=4 SV=1	0.0926 0749	1.23E-0 5	down
	Q6LWH7	repA	Copy-number control protein OS=Lactobacillus plantarum (strain ATCC BAA-793 / NCIMB 8826 / WCFS1) OX=220668 GN=repA PE=4 SV=1	0.0713 04472	6.19E-0 8	down
	F9URU9	lp_2847	Extracellular transglycosylase, with LysM peptidoglycan binding domain OS=Lactobacillus plantarum (strain ATCC BAA-793 / NCIMB 8826 / WCFS1) OX=220668 GN=lp_2847 PE=4 SV=1	0.1061 14574	0.00019 57	down
	F9UQA0	lp_2162	Extracellular protein, NlpC/P60 family,gamma-D-glutamate-meso-diaminopimelate muropeptidase OS=Lactobacillus plantarum (strain ATCC BAA-793 / NCIMB 8826 / WCFS1) OX=220668 GN=lp_2162 PE=3 SV=1	0.1378 05316	0.00016 13	down
	F9UTQ7	lp_0302	Extracellular transglycosylase OS=Lactobacillus plantarum (strain ATCC BAA-793 / NCIMB 8826 / WCFS1) OX=220668 GN=lp_0302 PE=4 SV=1	0.0897 00244	7.97E-0 5	down
	F9UU93	lp_3414	Cell surface protein, CscB family OS=Lactobacillus plantarum (strain ATCC BAA-793 / NCIMB 8826 / WCFS1) OX=220668 GN=lp_3414 PE=4 SV=1	0.1437 42975	0.00031 39	down
	F9USE1	lp_3014	Extracellular transglycosylase, with LysM peptidoglycan binding domain OS=Lactobacillus plantarum (strain ATCC BAA-793 / NCIMB 8826 / WCFS1) OX=220668 GN=lp_3014 PE=4 SV=1	0.1351 40674	0.00015 17	down
	F9UM79	lp_0869	Extracellular protein, Ser/Thr-rich OS=Lactobacillus plantarum (strain ATCC BAA-793 / NCIMB 8826 / WCFS1) OX=220668 GN=lp_0869 PE=4 SV=1	0.1794 77021	6.17E-0 5	down
	F9UP60	lp_1697	Adherence protein, chitin-binding domain OS=Lactobacillus plantarum (strain ATCC BAA-793 / NCIMB 8826 / WCFS1) OX=220668 GN=lp_1697 PE=4 SV=1	0.1241 3657	7.58E-0 5	down
	F9UUA0	lp_3421	Extracellular protein,gamma-D-glutamate-meso-diaminopimelate muropeptidase OS=Lactobacillus plantarum (strain ATCC BAA-793 / NCIMB 8826 / WCFS1) OX=220668 GN=lp_3421 PE=3 SV=1	0.1761 28432	4.17E-0 5	down
	P77889	Orotate phosphori bosyltrans ferase	Orotate phosphoribosyltransferase OS=Lactobacillus plantarum (strain ATCC BAA-793 / NCIMB 8826 / WCFS1) OX=220668 GN=pyrE PE=3 SV=1	0.2197 29799	1.45E-0 5	down
	F9USJ7	lp_3081	Transcription regulator, MarR family OS=Lactobacillus plantarum (strain ATCC BAA-793 / NCIMB 8826 / WCFS1) OX=220668 GN=lp_3081 PE=4 SV=1	0.3090 22773	2.62E-0 5	down
	F9UQZ9	lp_2463	Prophage P2b protein 18, major capsid protein OS=Lactobacillus plantarum (strain ATCC BAA-793 / NCIMB 8826 / WCFS1) OX=220668 GN=lp_2463 PE=4 SV=1	0.6400 14472	0.01286	down
	F9US24	lp_2940	Cell surface protein, LPXTG-motif cell wall anchor OS=Lactobacillus plantarum (strain ATCC BAA-793 / NCIMB 8826 / WCFS1) OX=220668 GN=lp_2940 PE=4 SV=1	0.1785 48979	0.00010 35	down
	F9UM68	lp_0856	Acyltransferase OS=Lactobacillus plantarum (strain ATCC BAA-793 / NCIMB 8826 / WCFS1) OX=220668 GN=lp_0856 PE=4 SV=1	0.2221 59412	8.23E-0 5	down
	Q890I8	Glycogen synthase	Glycogen synthase OS=Lactobacillus plantarum (strain ATCC BAA-793 / NCIMB 8826 / WCFS1) OX=220668 GN=glgA PE=3 SV=1	0.2283 35234	9.75E-0 5	down
	F9URS2	lp_2809	Extracellular protein OS=Lactobacillus plantarum (strain ATCC BAA-793 / NCIMB 8826 / WCFS1) OX=220668 GN=lp_2809 PE=4 SV=1	0.2667 16152	0.00023 33	down
	F9UR89	lp_2575	Transporter, MMPL family OS=Lactobacillus plantarum (strain ATCC BAA-793 / NCIMB 8826 / WCFS1) OX=220668 GN=lp_2575 PE=4 SV=1	0.6454 22943	0.00934 2	down
	F9URU8	lp_2845	Extracellular transglycosylase, with LysM peptidoglycan binding domain OS=Lactobacillus plantarum (strain ATCC BAA-793 / NCIMB 8826 / WCFS1) OX=220668 GN=lp_2845 PE=4 SV=1	0.2012 89029	0.00013 84	down
	P77885	Carbamo yl-phosph	Carbamoyl-phosphate synthase pyrimidine-specific small chain OS=Lactobacillus plantarum (strain ATCC BAA-793 / NCIMB 8826 /	0.3890 2148	3.68E-0 6	down

	ate synthase pyrimidine-specific small chain	WCFS1) OX=220668 GN=pyrAA PE=3 SV=2			
F9URS4	lp_2812	Extracellular protein, membrane-anchored OS=Lactobacillus plantarum (strain ATCC BAA-793 / NCIMB 8826 / WCFS1) OX=220668 GN=lp_2812 PE=4 SV=1	0.184334511	0.0002759	down
F9UKV7	lp_0473	Lipoprotein OS=Lactobacillus plantarum (strain ATCC BAA-793 / NCIMB 8826 / WCFS1) OX=220668 GN=lp_0473 PE=4 SV=1	0.345753609	0.0002714	down
P77887	Dihydroorotate dehydrogenase A (fumarate)	Dihydroorotate dehydrogenase A (fumarate) OS=Lactobacillus plantarum (strain ATCC BAA-793 / NCIMB 8826 / WCFS1) OX=220668 GN=pyrD PE=3 SV=2	0.331780366	1.97E-05	down
F9USV1	hsp1	Small heat shock protein OS=Lactobacillus plantarum (strain ATCC BAA-793 / NCIMB 8826 / WCFS1) OX=220668 GN=hsp1 PE=3 SV=1	0.276636714	3.48E-05	down
F9USB8	Conserved domain protein	Uncharacterized protein OS=Lactobacillus plantarum (strain ATCC BAA-793 / NCIMB 8826 / WCFS1) OX=220668 GN=lp_2987 PE=4 SV=1	0.215030675	7.13E-06	down
F9UTM5	hsp3	Small heat shock protein OS=Lactobacillus plantarum (strain ATCC BAA-793 / NCIMB 8826 / WCFS1) OX=220668 GN=hsp3 PE=3 SV=1	0.375131349	1.31E-05	down
F9UT05	lp_0197	Cell surface protein, LPXTG-motif cell wall anchor OS=Lactobacillus plantarum (strain ATCC BAA-793 / NCIMB 8826 / WCFS1) OX=220668 GN=lp_0197 PE=4 SV=1	0.364775496	0.0001406	down
F9UME2	lp_0946	Mucus-binding protein, LPXTG-motif cell wall anchor OS=Lactobacillus plantarum (strain ATCC BAA-793 / NCIMB 8826 / WCFS1) OX=220668 GN=lp_0946 PE=4 SV=1	0.304273217	0.001244	down
F9UMC3	lp_0924	Extracellular protein, MORN repeat family OS=Lactobacillus plantarum (strain ATCC BAA-793 / NCIMB 8826 / WCFS1) OX=220668 GN=lp_0924 PE=4 SV=1	0.235030419	0.0001425	down
F9URE0	pts19A	PTS system,N-acetylglucosamine/galactosamine-specific EIIA component OS=Lactobacillus plantarum (strain ATCC BAA-793 / NCIMB 8826 / WCFS1) OX=220668 GN=pts19A PE=4 SV=1	0.101670507	0.0002078	down
Q88VY3	UPF0756 membrane protein lp_1894	UPF0756 membrane protein lp_1894 OS=Lactobacillus plantarum (strain ATCC BAA-793 / NCIMB 8826 / WCFS1) OX=220668 GN=lp_1894 PE=3 SV=1	0.305152868	0.000226	down
F9UU85	Inner membrane protein	Uncharacterized protein OS=Lactobacillus plantarum (strain ATCC BAA-793 / NCIMB 8826 / WCFS1) OX=220668 GN=lp_3406 PE=4 SV=1	0.274311927	0.0003834	down
F9UNG3	30S ribosomal protein S21	Uncharacterized protein OS=Lactobacillus plantarum (strain ATCC BAA-793 / NCIMB 8826 / WCFS1) OX=220668 GN=lp_1412 PE=4 SV=1	0.434200459	0.001747	down
F9UQX6	lp_2436	Prophage P2a protein 21 OS=Lactobacillus plantarum (strain ATCC BAA-793 / NCIMB 8826 / WCFS1) OX=220668 GN=lp_2436 PE=4 SV=1	0.577711358	0.0004689	down
F9US93	wapA	Cell surface protein, LPXTG-motif cell wall anchor OS=Lactobacillus plantarum (strain ATCC BAA-793 / NCIMB 8826 / WCFS1) OX=220668 GN=wapA PE=4 SV=1	0.33271806	9.97E-05	down
F9UU91	lp_3412	Cell surface protein, CscB family OS=Lactobacillus plantarum (strain ATCC BAA-793 / NCIMB 8826 / WCFS1) OX=220668 GN=lp_3412 PE=4 SV=1	0.219833887	3.78E-05	down
F9USJ2	lp_3075	Cell surface protein, CscC family OS=Lactobacillus plantarum (strain ATCC BAA-793 / NCIMB 8826 / WCFS1) OX=220668 GN=lp_3075 PE=4 SV=1	0.383773585	0.02619	down
F9US95	lp_2960	Lipase/esterase, subfamily of SGNH-hydrolases OS=Lactobacillus plantarum (strain ATCC BAA-793 / NCIMB 8826 / WCFS1) OX=220668 GN=lp_2960 PE=4 SV=1	0.288916607	4.03E-05	down
F9URZ8	lp_2909	Acetyltransferase, GNAT family OS=Lactobacillus plantarum (strain ATCC BAA-793 / NCIMB 8826 / WCFS1) OX=220668 GN=lp_2909 PE=4 SV=1	0.489677952	0.01606	down
P77883	Aspartate carbamoyltransferase	Aspartate carbamoyltransferase OS=Lactobacillus plantarum (strain ATCC BAA-793 / NCIMB 8826 / WCFS1) OX=220668 GN=pyrB PE=3 SV=1	0.336423077	2.81E-05	down
Q88YM6	Co-chaperonin GroES	Co-chaperonin GroES OS=Lactobacillus plantarum (strain ATCC BAA-793 / NCIMB 8826 / WCFS1) OX=220668 GN=groES PE=3 SV=1	0.492261393	0.0001777	down

F9UQT3	Glyco_hydro_38C domain-containing protein	Uncharacterized protein OS=Lactobacillus plantarum (strain ATCC BAA-793 / NCIMB 8826 / WCFS1) OX=220668 GN=lp_2391 PE=4 SV=1	0.646248813	0.003574	down
F9UMC2	lp_0923	Cell surface protein, LPXTG-motif cell wall anchor OS=Lactobacillus plantarum (strain ATCC BAA-793 / NCIMB 8826 / WCFS1) OX=220668 GN=lp_0923 PE=4 SV=1	0.325184037	0.0009277	down
F9UNC6	Pseudouridylate synthase	Uncharacterized protein OS=Lactobacillus plantarum (strain ATCC BAA-793 / NCIMB 8826 / WCFS1) OX=220668 GN=lp_1362 PE=4 SV=1	0.421556388	1.54E-05	down
F9UUC3	lp_3450	Cell surface protein, CscC family OS=Lactobacillus plantarum (strain ATCC BAA-793 / NCIMB 8826 / WCFS1) OX=220668 GN=lp_3450 PE=4 SV=1	0.388089431	0.0003391	down
F9USW1	lp_0141	Extracellular protein OS=Lactobacillus plantarum (strain ATCC BAA-793 / NCIMB 8826 / WCFS1) OX=220668 GN=lp_0141 PE=4 SV=1	0.254889472	8.50E-05	down
F9UU40	Ribonuclease M5	Ribonuclease M5 OS=Lactobacillus plantarum (strain ATCC BAA-793 / NCIMB 8826 / WCFS1) OX=220668 GN=rnmV PE=3 SV=1	0.469414894	9.73E-05	down
F9UMD1	lp_0932	Extracellular lipoprotein OS=Lactobacillus plantarum (strain ATCC BAA-793 / NCIMB 8826 / WCFS1) OX=220668 GN=lp_0932 PE=4 SV=1	0.390335052	0.0006114	down
F9URD4	lp_2636	Extracellular protein OS=Lactobacillus plantarum (strain ATCC BAA-793 / NCIMB 8826 / WCFS1) OX=220668 GN=lp_2636 PE=4 SV=1	0.273517787	0.0002554	down
F9UNI8	lp_1446	Cell surface protein, CscB family OS=Lactobacillus plantarum (strain ATCC BAA-793 / NCIMB 8826 / WCFS1) OX=220668 GN=lp_1446 PE=4 SV=1	0.410879218	0.009866	down
F9ULL9	lp_3676	Cell surface protein, CscC family OS=Lactobacillus plantarum (strain ATCC BAA-793 / NCIMB 8826 / WCFS1) OX=220668 GN=lp_3676 PE=4 SV=1	0.370064795	0.0006925	down
F9UPB8	lp_1767	Glycosyl hydrolase, family 25 OS=Lactobacillus plantarum (strain ATCC BAA-793 / NCIMB 8826 / WCFS1) OX=220668 GN=lp_1767 PE=3 SV=1	0.384316295	0.0005113	down
F9UMH5	lp_0988	Extracellular lipoprotein, Asp-rich OS=Lactobacillus plantarum (strain ATCC BAA-793 / NCIMB 8826 / WCFS1) OX=220668 GN=lp_0988 PE=4 SV=1	0.358818419	0.0001763	down
F9UTQ8	lp_0304	Extracellular transglycosylase OS=Lactobacillus plantarum (strain ATCC BAA-793 / NCIMB 8826 / WCFS1) OX=220668 GN=lp_0304 PE=4 SV=1	0.372827041	3.81E-05	down
F9UL76	Mini-ribonuclease 3	Mini-ribonuclease 3 OS=Lactobacillus plantarum (strain ATCC BAA-793 / NCIMB 8826 / WCFS1) OX=220668 GN=mrnC PE=3 SV=1	2.12900633	0.01896	up
F9ULX3	Phosphate-binding protein	Phosphate-binding protein OS=Lactobacillus plantarum (strain ATCC BAA-793 / NCIMB 8826 / WCFS1) OX=220668 GN=pstE PE=3 SV=1	0.488147073	4.90E-05	down
F9USW8	lp_0154	Transcription regulator, PadR family OS=Lactobacillus plantarum (strain ATCC BAA-793 / NCIMB 8826 / WCFS1) OX=220668 GN=lp_0154 PE=4 SV=1	0.464849354	3.41E-05	down
Q88X33	UPF0342 protein lp_1415	UPF0342 protein lp_1415 OS=Lactobacillus plantarum (strain ATCC BAA-793 / NCIMB 8826 / WCFS1) OX=220668 GN=lp_1415 PE=3 SV=1	0.6171459	0.000563	down
F9UM52	spx1	RNA polymerase (RNAP)-binding regulatory protein, arsenate reductase (ArsC) family, Spx subfamily OS=Lactobacillus plantarum (strain ATCC BAA-793 / NCIMB 8826 / WCFS1) OX=220668 GN=spx1 PE=3 SV=1	0.249481973	9.63E-07	down
F9UNC2	lp_1357	Extracellular protein, membrane-anchored OS=Lactobacillus plantarum (strain ATCC BAA-793 / NCIMB 8826 / WCFS1) OX=220668 GN=lp_1357 PE=4 SV=1	0.328918322	0.0005127	down
F9ULN5	lp_0646	Prophage P1 protein 23 OS=Lactobacillus plantarum (strain ATCC BAA-793 / NCIMB 8826 / WCFS1) OX=220668 GN=lp_0646 PE=4 SV=1	0.448026949	0.02058	down
F9URC0	lp_2616	Bacteriocin immunity protein OS=Lactobacillus plantarum (strain ATCC BAA-793 / NCIMB 8826 / WCFS1) OX=220668 GN=lp_2616 PE=4 SV=1	0.61352657	0.0002366	down
F9UQH9	lp_2260	Extracellular protein, DUF336 family OS=Lactobacillus plantarum (strain ATCC BAA-793 / NCIMB 8826 / WCFS1) OX=220668 GN=lp_2260 PE=4 SV=1	0.604378003	0.001426	down
F9UT96	lp_3185	Branched-chain amino acid transport protein OS=Lactobacillus plantarum (strain ATCC BAA-793 / NCIMB 8826 / WCFS1) OX=220668 GN=lp_3185 PE=3 SV=1	0.583864119	0.001533	down
F9USM7	lp_3114	Mucus-binding protein, LPXTG-motif cell wall anchor OS=Lactobacillus plantarum (strain ATCC BAA-793 / NCIMB 8826 / WCFS1) OX=220668 GN=lp_3114 PE=4 SV=1	0.360615809	0.0005055	down
F9UMC4	lp_0925	Acyltransferase OS=Lactobacillus plantarum (strain ATCC BAA-793 / NCIMB 8826 / WCFS1) OX=220668 GN=lp_0925 PE=4 SV=1	0.240998741	0.0001627	down

F9URK4	N5-carboxyaminoimidazole ribonucleotide synthase	N5-carboxyaminoimidazole ribonucleotide synthase OS=Lactobacillus plantarum (strain ATCC BAA-793 / NCIMB 8826 / WCFS1) OX=220668 GN=purK1 PE=3 SV=1	0.545216937	0.0006215	down
F9URZ3	lp_2901	Hypothetical membrane protein OS=Lactobacillus plantarum (strain ATCC BAA-793 / NCIMB 8826 / WCFS1) OX=220668 GN=lp_2901 PE=4 SV=1	0.371249419	0.0009142	down
Q88VJ7	Ribosome-recycling factor	Ribosome-recycling factor OS=Lactobacillus plantarum (strain ATCC BAA-793 / NCIMB 8826 / WCFS1) OX=220668 GN=frr PE=3 SV=1	0.617971334	0.0001151	down
F9UT63	DUF4123 domain-containing protein	Uncharacterized protein OS=Lactobacillus plantarum (strain ATCC BAA-793 / NCIMB 8826 / WCFS1) OX=220668 GN=lp_0266 PE=4 SV=1	0.543386243	0.0001163	down
F9UTM7	lp_3355	Short-chain dehydrogenase/oxidoreductase,atypical SDR family, subgroup 6 OS=Lactobacillus plantarum (strain ATCC BAA-793 / NCIMB 8826 / WCFS1) OX=220668 GN=lp_3355 PE=4 SV=1	0.439108911	8.92E-05	down
F9URS3	lp_2810	Glycosyl hydrolase, family 25 OS=Lactobacillus plantarum (strain ATCC BAA-793 / NCIMB 8826 / WCFS1) OX=220668 GN=lp_2810 PE=3 SV=1	0.577217963	0.001499	down
F9UTV9	Biotin carboxyl carrier protein of acetyl-CoA carboxylase	Biotin carboxyl carrier protein of acetyl-CoA carboxylase OS=Lactobacillus plantarum (strain ATCC BAA-793 / NCIMB 8826 / WCFS1) OX=220668 GN=accB3 PE=4 SV=1	0.520707871	2.76E-05	down
F9USL7	fhuD	Iron chelatin ABC transporter, substrate binding protein OS=Lactobacillus plantarum (strain ATCC BAA-793 / NCIMB 8826 / WCFS1) OX=220668 GN=fhuD PE=4 SV=1	0.410433071	0.0003977	down
F9UPF5	lp_1812	Lipoprotein OS=Lactobacillus plantarum (strain ATCC BAA-793 / NCIMB 8826 / WCFS1) OX=220668 GN=lp_1812 PE=4 SV=1	0.383341475	0.0002157	down
F9US78	lp_0058	Flavoprotein OS=Lactobacillus plantarum (strain ATCC BAA-793 / NCIMB 8826 / WCFS1) OX=220668 GN=lp_0058 PE=4 SV=1	0.564614531	1.29E-05	down
F9UNT7	lp_1557	Transcription regulator, MarR family OS=Lactobacillus plantarum (strain ATCC BAA-793 / NCIMB 8826 / WCFS1) OX=220668 GN=lp_1557 PE=4 SV=1	1.682296651	7.43E-06	up
F9URD9	acm2	Cell wall hydrolase/muramidase OS=Lactobacillus plantarum (strain ATCC BAA-793 / NCIMB 8826 / WCFS1) OX=220668 GN=acm2 PE=3 SV=1	0.356768559	9.39E-05	down
F9UQY6	lp_2447	Prophage P2a protein 10, phage transcription regulator, Cro/CI family OS=Lactobacillus plantarum (strain ATCC BAA-793 / NCIMB 8826 / WCFS1) OX=220668 GN=lp_2447 PE=4 SV=1	0.459884937	0.0009861	down
F9UTC0	lp_3216	Transcription regulator, PadR family OS=Lactobacillus plantarum (strain ATCC BAA-793 / NCIMB 8826 / WCFS1) OX=220668 GN=lp_3216 PE=4 SV=1	0.611111111	0.01226	down
F9USK7	lp_3093	Lysozyme/muramidase, glycoside hydrolase family 25 OS=Lactobacillus plantarum (strain ATCC BAA-793 / NCIMB 8826 / WCFS1) OX=220668 GN=lp_3093 PE=3 SV=1	0.316841104	0.0001697	down
F9UTP5	lp_0290	Transcriptional attenuator, cell envelope-related, LytR family OS=Lactobacillus plantarum (strain ATCC BAA-793 / NCIMB 8826 / WCFS1) OX=220668 GN=lp_0290 PE=3 SV=1	0.423556701	0.0001837	down
F9USJ9	lp_3084	Cell surface protein, ErfK family OS=Lactobacillus plantarum (strain ATCC BAA-793 / NCIMB 8826 / WCFS1) OX=220668 GN=lp_3084 PE=4 SV=1	0.357423795	0.001844	down
F9UT67	Flavin prenyltransferase LpdB	Flavin prenyltransferase LpdB OS=Lactobacillus plantarum (strain ATCC BAA-793 / NCIMB 8826 / WCFS1) OX=220668 GN=lpdB PE=3 SV=1	0.624851367	0.000292	down
F9UQL7	lp_2306	Non-proteolytic protein, peptidase family M16 OS=Lactobacillus plantarum (strain ATCC BAA-793 / NCIMB 8826 / WCFS1) OX=220668 GN=lp_2306 PE=4 SV=1	0.634892086	0.0003712	down
Q88WN3	50S ribosomal protein L27	50S ribosomal protein L27 OS=Lactobacillus plantarum (strain ATCC BAA-793 / NCIMB 8826 / WCFS1) OX=220668 GN=rpmA PE=3 SV=1	0.667694205	0.003145	down
F9UQH8	lp_2259	Transcription regulator, MerR family OS=Lactobacillus plantarum (strain ATCC BAA-793 / NCIMB 8826 / WCFS1) OX=220668	0.517410967	0.00176	down

		GN=lp_2259 PE=4 SV=1			
Q6LWD8	Mobilization protein	Uncharacterized protein OS=Lactobacillus plantarum (strain ATCC BAA-793 / NCIMB 8826 / WCFS1) OX=220668 GN=orf39 PE=4 SV=1	0.368689444	0.0007928	down
F9UPN8	lp_1915	Lipoprotein OS=Lactobacillus plantarum (strain ATCC BAA-793 / NCIMB 8826 / WCFS1) OX=220668 GN=lp_1915 PE=4 SV=1	0.389320892	0.000459	down
Q88S51	L-rhamnose isomerase	L-rhamnose isomerase OS=Lactobacillus plantarum (strain ATCC BAA-793 / NCIMB 8826 / WCFS1) OX=220668 GN=rhaA PE=3 SV=1	0.411544544	0.0002689	down
F9UQ26	lp_2075	Transcriptional attenuator, cell envelope-related, LytR family OS=Lactobacillus plantarum (strain ATCC BAA-793 / NCIMB 8826 / WCFS1) OX=220668 GN=lp_2075 PE=3 SV=1	0.420977164	0.0004271	down
F9UMI0	PhaC_N domain-containing protein	Uncharacterized protein OS=Lactobacillus plantarum (strain ATCC BAA-793 / NCIMB 8826 / WCFS1) OX=220668 GN=lp_0995 PE=4 SV=1	0.656483791	0.004092	down
F9US28	zinc_ribbon_2 domain-containing protein	zinc_ribbon_2 domain-containing protein OS=Lactobacillus plantarum (strain ATCC BAA-793 / NCIMB 8826 / WCFS1) OX=220668 GN=lp_2948 PE=4 SV=1	0.604413543	0.0003215	down
F9US02	lp_2914	Hypothetical membrane protein, DUF2207 family OS=Lactobacillus plantarum (strain ATCC BAA-793 / NCIMB 8826 / WCFS1) OX=220668 GN=lp_2914 PE=4 SV=1	0.504555809	0.0002506	down
F9ULD4	spx5	RNA polymerase (RNAP)-binding regulatory protein, arsenate reductase (ArsC) family, Spx subfamily OS=Lactobacillus plantarum (strain ATCC BAA-793 / NCIMB 8826 / WCFS1) OX=220668 GN=spx5 PE=3 SV=1	0.445888158	1.35E-05	down
Q88VL9	Protein GrpE	Protein GrpE OS=Lactobacillus plantarum (strain ATCC BAA-793 / NCIMB 8826 / WCFS1) OX=220668 GN=grpE PE=3 SV=1	0.614718615	9.76E-05	down
F9UQ45	lp_2098	Metallophosphoesterase, lipoprotein OS=Lactobacillus plantarum (strain ATCC BAA-793 / NCIMB 8826 / WCFS1) OX=220668 GN=lp_2098 PE=4 SV=1	0.430858086	0.0002108	down
P71479	Bifunctional protein PyrR 1	Bifunctional protein PyrR 1 OS=Lactobacillus plantarum (strain ATCC BAA-793 / NCIMB 8826 / WCFS1) OX=220668 GN=pyrR1 PE=3 SV=1	0.492135476	2.60E-06	down
F9UM21	lp_0800	Cell surface protein, LPXTG-motif cell wall anchor OS=Lactobacillus plantarum (strain ATCC BAA-793 / NCIMB 8826 / WCFS1) OX=220668 GN=lp_0800 PE=4 SV=1	0.470290268	0.0009002	down
F9UPT6	GatB/YqeY domain-containing protein	Uncharacterized protein OS=Lactobacillus plantarum (strain ATCC BAA-793 / NCIMB 8826 / WCFS1) OX=220668 GN=lp_1972 PE=4 SV=1	0.635436584	0.0008837	down
Q88XP8	Aspartyl/glutamyl-tRNA(Asn/Gln) amidotransferase subunit C	Aspartyl/glutamyl-tRNA(Asn/Gln) amidotransferase subunit C OS=Lactobacillus plantarum (strain ATCC BAA-793 / NCIMB 8826 / WCFS1) OX=220668 GN=gatC PE=3 SV=1	0.65374677	0.00169	down
F9UQC5	Cell ivision protein DivIB	Cell division protein DivIB OS=Lactobacillus plantarum (strain ATCC BAA-793 / NCIMB 8826 / WCFS1) OX=220668 GN=divIB PE=3 SV=1	0.502469136	5.02E-05	down
F9UPA5	pbp1A	DD-transpeptidase OS=Lactobacillus plantarum (strain ATCC BAA-793 / NCIMB 8826 / WCFS1) OX=220668 GN=pbp1A PE=4 SV=1	0.607876497	0.0003024	down
F9UL67	zmp2	Extracellular zinc metalloproteinase, M10 family OS=Lactobacillus plantarum (strain ATCC BAA-793 / NCIMB 8826 / WCFS1) OX=220668 GN=zmp2 PE=4 SV=1	0.563811297	0.0009775	down
F9UTM2	Adenine DNA glycosylase	Adenine DNA glycosylase OS=Lactobacillus plantarum (strain ATCC BAA-793 / NCIMB 8826 / WCFS1) OX=220668 GN=mutY PE=3 SV=1	0.460417876	4.75E-05	down
F9UQY4	lp_2445	Prophage P2a protein 12 OS=Lactobacillus plantarum (strain ATCC BAA-793 / NCIMB 8826 / WCFS1) OX=220668 GN=lp_2445 PE=4 SV=1	0.504186603	0.001995	down
F9UR96	lp_2586	Cell surface hydrolase, DUF915 family,membrane-bound OS=Lactobacillus plantarum (strain ATCC BAA-793 / NCIMB 8826 / WCFS1) OX=220668 GN=lp_2586 PE=4 SV=1	0.569725343	3.78E-05	down
F9URZ6	endA	DNA-entry nuclease OS=Lactobacillus plantarum (strain ATCC BAA-793 / NCIMB 8826 / WCFS1) OX=220668 GN=endA PE=4 SV=1	0.486856128	0.002333	down

F9UU92	lp_3413	Cell surface protein, CscA/DUF916 family OS=Lactobacillus plantarum (strain ATCC BAA-793 / NCIMB 8826 / WCFS1) OX=220668 GN=lp_3413 PE=4 SV=1	0.624837451	0.0005579	down
F9URW1	Endonuclease III	Endonuclease III OS=Lactobacillus plantarum (strain ATCC BAA-793 / NCIMB 8826 / WCFS1) OX=220668 GN=nth1 PE=3 SV=1	1.565942551	0.01596	up
F9US19	lp_2934	Lipoprotein OS=Lactobacillus plantarum (strain ATCC BAA-793 / NCIMB 8826 / WCFS1) OX=220668 GN=lp_2934 PE=4 SV=1	0.367278798	8.72E-05	down
F9UMW3	Universal stress protein	Universal stress protein OS=Lactobacillus plantarum (strain ATCC BAA-793 / NCIMB 8826 / WCFS1) OX=220668 GN=lp_1163 PE=1 SV=1	0.641219839	0.002281	down
Q88YP9	Nucleoid-associated protein lp_0699	Nucleoid-associated protein lp_0699 OS=Lactobacillus plantarum (strain ATCC BAA-793 / NCIMB 8826 / WCFS1) OX=220668 GN=lp_0699 PE=3 SV=1	0.580659767	0.0003486	down
Q88UT8	ATP synthase subunit c	ATP synthase subunit c OS=Lactobacillus plantarum (strain ATCC BAA-793 / NCIMB 8826 / WCFS1) OX=220668 GN=atpE PE=3 SV=1	1.622124863	0.009475	up
F9URL1	lp_2737	Cell surface hydrolase, DUF915 family,membrane-bound OS=Lactobacillus plantarum (strain ATCC BAA-793 / NCIMB 8826 / WCFS1) OX=220668 GN=lp_2737 PE=4 SV=1	0.450242718	9.40E-05	down
F9UL16	divIC	Septum formation initiator OS=Lactobacillus plantarum (strain ATCC BAA-793 / NCIMB 8826 / WCFS1) OX=220668 GN=divIC PE=4 SV=1	0.641443299	3.59E-05	down
F9US12	lp_2925	Cell surface protein, LPXTG-motif cell wall anchor OS=Lactobacillus plantarum (strain ATCC BAA-793 / NCIMB 8826 / WCFS1) OX=220668 GN=lp_2925 PE=4 SV=1	0.500125707	0.002582	down
F9UMH9	lp_0992	Transcription regulator, MerR family OS=Lactobacillus plantarum (strain ATCC BAA-793 / NCIMB 8826 / WCFS1) OX=220668 GN=lp_0992 PE=4 SV=1	2.216376636	0.0004271	up
F9UUJ0	pbg10	6-phospho-beta-glucosidase OS=Lactobacillus plantarum (strain ATCC BAA-793 / NCIMB 8826 / WCFS1) OX=220668 GN=pbg10 PE=3 SV=1	0.530283505	0.0004022	down
F9UQ61	lp_2114	NTP pyrophosphohydrolase OS=Lactobacillus plantarum (strain ATCC BAA-793 / NCIMB 8826 / WCFS1) OX=220668 GN=lp_2114 PE=4 SV=1	0.597708895	0.000192	down
F9UR61	lp_2541	ABC transporter, substrate binding protein OS=Lactobacillus plantarum (strain ATCC BAA-793 / NCIMB 8826 / WCFS1) OX=220668 GN=lp_2541 PE=4 SV=1	0.519114817	0.0002295	down
F9UTL5	lp_3341	Cell surface hydrolase, DUF915 family,membrane-bound OS=Lactobacillus plantarum (strain ATCC BAA-793 / NCIMB 8826 / WCFS1) OX=220668 GN=lp_3341 PE=4 SV=1	0.417299578	0.001283	down
F9UN64	dacA2	Serine-type D-Ala-D-Ala carboxypeptidase OS=Lactobacillus plantarum (strain ATCC BAA-793 / NCIMB 8826 / WCFS1) OX=220668 GN=dacA2 PE=3 SV=1	0.442260442	0.0001779	down
F9UMU1	acm1	Cell wall hydrolase/muramidase OS=Lactobacillus plantarum (strain ATCC BAA-793 / NCIMB 8826 / WCFS1) OX=220668 GN=acm1 PE=3 SV=1	0.497002551	0.01902	down
F9UPA0	lp_1746	D-methionine ABC transporter, substrate binding protein OS=Lactobacillus plantarum (strain ATCC BAA-793 / NCIMB 8826 / WCFS1) OX=220668 GN=lp_1746 PE=3 SV=1	0.33847032	5.54E-05	down
F9UMF7	DNA helicase	DNA helicase OS=Lactobacillus plantarum (strain ATCC BAA-793 / NCIMB 8826 / WCFS1) OX=220668 GN=recQ1 PE=3 SV=1	0.659560907	0.04214	down
F9UUF7	lp_3491	Fumarate reductase, flavoprotein subunit OS=Lactobacillus plantarum (strain ATCC BAA-793 / NCIMB 8826 / WCFS1) OX=220668 GN=lp_3491 PE=1 SV=1	1.646099853	1.06E-05	up
F9UN26	lp_1233	Priming glycosyltransferase,undecaprenyl-phosphate beta-glucosephosphotransferase OS=Lactobacillus plantarum (strain ATCC BAA-793 / NCIMB 8826 / WCFS1) OX=220668 GN=lp_1233 PE=3 SV=1	2.560994323	3.62E-06	up
F9UMX0	pbpX1	Serine-type D-Ala-D-Ala carboxypeptidase OS=Lactobacillus plantarum (strain ATCC BAA-793 / NCIMB 8826 / WCFS1) OX=220668 GN=pbpX1 PE=4 SV=1	0.607911173	0.001047	down
F9UMN8	lp_1070	Lipoprotein, FMN-binding protein OS=Lactobacillus plantarum (strain ATCC BAA-793 / NCIMB 8826 / WCFS1) OX=220668 GN=lp_1070 PE=4 SV=1	0.51289267	0.0001661	down
F9UM73	AP2/ERF domain-containing protein	AP2/ERF domain-containing protein OS=Lactobacillus plantarum (strain ATCC BAA-793 / NCIMB 8826 / WCFS1) OX=220668 GN=lp_0862 PE=4 SV=1	2.509893455	9.90E-06	up
F9ULS6	lp_0689	Cell surface protein, lipoprotein OS=Lactobacillus plantarum (strain ATCC BAA-793 / NCIMB 8826 / WCFS1) OX=220668 GN=lp_0689 PE=4 SV=1	0.549763033	0.001583	down
F9UMC7	DUF2273 domain-c	Uncharacterized protein OS=Lactobacillus plantarum (strain ATCC BAA-793 / NCIMB 8826 / WCFS1) OX=220668 GN=lp_0928 PE=4 SV=1	1.579780617	0.003247	up

	ontaining protein				
F9ULM1	lp_3678	Cell surface protein, CscA/DUF916 family OS=Lactobacillus plantarum (strain ATCC BAA-793 / NCIMB 8826 / WCFS1) OX=220668 GN=lp_3678 PE=4 SV=1	0.4562 30032	0.00169 1	down
F9UKY5	Transcript ional regulator	Uncharacterized protein OS=Lactobacillus plantarum (strain ATCC BAA-793 / NCIMB 8826 / WCFS1) OX=220668 GN=lp_0507 PE=4 SV=1	1.8403 99002	2.78E-0 5	up
F9USF3	lp_3026	Short-chain dehydrogenase/oxidoreductase OS=Lactobacillus plantarum (strain ATCC BAA-793 / NCIMB 8826 / WCFS1) OX=220668 GN=lp_3026 PE=3 SV=1	0.6155 42938	0.03091	down
F9UR02	lp_2467	Prophage P2b protein 14, terminase small subunit OS=Lactobacillus plantarum (strain ATCC BAA-793 / NCIMB 8826 / WCFS1) OX=220668 GN=lp_2467 PE=4 SV=1	0.5378 37838	0.00149 4	down
F9UQX4	lp_2434	Prophage P2a protein 23 OS=Lactobacillus plantarum (strain ATCC BAA-793 / NCIMB 8826 / WCFS1) OX=220668 GN=lp_2434 PE=4 SV=1	0.4942 83837	0.00182 4	down
F9UR00	lp_2464	Prophage P2b protein 17, portal protein OS=Lactobacillus plantarum (strain ATCC BAA-793 / NCIMB 8826 / WCFS1) OX=220668 GN=lp_2464 PE=4 SV=1	0.6608 47334	0.03383	down
F9ULW6	Ribosome hibernatio n promotin g factor	Ribosome hibernation promoting factor OS=Lactobacillus plantarum (strain ATCC BAA-793 / NCIMB 8826 / WCFS1) OX=220668 GN=hpf PE=3 SV=1	0.5884 83146	3.37E-0 6	down
F9UNS3	lp_1539	Lipoprotein OS=Lactobacillus plantarum (strain ATCC BAA-793 / NCIMB 8826 / WCFS1) OX=220668 GN=lp_1539 PE=4 SV=1	0.6332 60712	0.00040 3	down
Q6LWH6	orf1	DNA-damage-inducible protein OS=Lactobacillus plantarum (strain ATCC BAA-793 / NCIMB 8826 / WCFS1) OX=220668 GN=orf1 PE=3 SV=1	0.6561 11929	0.00040 98	down
F9URT0	lp_2822	ABC transporter, permease protein OS=Lactobacillus plantarum (strain ATCC BAA-793 / NCIMB 8826 / WCFS1) OX=220668 GN=lp_2822 PE=4 SV=1	0.6091 62491	0.00019 92	down
Q88T16	Foldase protein PrsA 2	Foldase protein PrsA 2 OS=Lactobacillus plantarum (strain ATCC BAA-793 / NCIMB 8826 / WCFS1) OX=220668 GN=prsA2 PE=3 SV=1	0.6040 83095	0.00376 7	down
F9URD1	trxH	Thioredoxin H-type OS=Lactobacillus plantarum (strain ATCC BAA-793 / NCIMB 8826 / WCFS1) OX=220668 GN=trxH PE=4 SV=1	0.6182 21258	0.00086 49	down
F9UQX7	lp_2437	Prophage P2a protein 20, replication protein DnaD domain OS=Lactobacillus plantarum (strain ATCC BAA-793 / NCIMB 8826 / WCFS1) OX=220668 GN=lp_2437 PE=4 SV=1	0.6	0.02148	down
F9URR6	lp_2800	Transcription regulator. MarR family OS=Lactobacillus plantarum (strain ATCC BAA-793 / NCIMB 8826 / WCFS1) OX=220668 GN=lp_2800 PE=4 SV=1	1.7097 56098	0.00020 23	up
F9USD6	pts23B	PTS system, cellobiose-specific EIIB component OS=Lactobacillus plantarum (strain ATCC BAA-793 / NCIMB 8826 / WCFS1) OX=220668 GN=pts23B PE=4 SV=1	0.3785 31425	4.82E-0 6	down
Q88YF4	UDP-N-ac etylenolp yruvoylgl ucosamin e reductase	UDP-N-acetylenolpyruvoylglucosamine reductase OS=Lactobacillus plantarum (strain ATCC BAA-793 / NCIMB 8826 / WCFS1) OX=220668 GN=murB PE=3 SV=2	1.5234 28571	0.00027 96	up
F9UNX7	lp_1602	Polyprenyl synthetase OS=Lactobacillus plantarum (strain ATCC BAA-793 / NCIMB 8826 / WCFS1) OX=220668 GN=lp_1602 PE=3 SV=1	1.5674 4186	0.00308 6	up
F9UKV4	bla1	Beta-lactamase OS=Lactobacillus plantarum (strain ATCC BAA-793 / NCIMB 8826 / WCFS1) OX=220668 GN=bla1 PE=4 SV=1	0.6270 77151	0.00648 7	down
F9UTB9	Zinc_ribb on_2 domain-c ontaining protein	Uncharacterized protein OS=Lactobacillus plantarum (strain ATCC BAA-793 / NCIMB 8826 / WCFS1) OX=220668 GN=lp_3215 PE=4 SV=1	0.4710 63257	0.00035 07	down
F9USK5	lp_3091	Short-chain dehydrogenase/oxidoreductase,atypical SDR family, subgroup 1 OS=Lactobacillus plantarum (strain ATCC BAA-793 / NCIMB 8826 / WCFS1) OX=220668 GN=lp_3091 PE=4 SV=1	0.6517 42424	9.02E-0 6	down
F9ULS5	lp_0688	DNA entry nuclease OS=Lactobacillus plantarum (strain ATCC BAA-793 / NCIMB 8826 / WCFS1) OX=220668 GN=lp_0688 PE=4 SV=1	0.5053 1768	0.00181 4	down
Q88W97	Holliday junction resolvase RecU	Holliday junction resolvase RecU OS=Lactobacillus plantarum (strain ATCC BAA-793 / NCIMB 8826 / WCFS1) OX=220668 GN=recU PE=3 SV=1	2.1859 85015	2.47E-0 6	up

F9US20	def2	Formylmethionine deformylase OS=Lactobacillus plantarum (strain ATCC BAA-793 / NCIMB 8826 / WCFS1) OX=220668 GN=def2 PE=3 SV=1	1.845261632	0.0002593	up
F9URZ5	lp_2903	Transcription regulator, ArsR family OS=Lactobacillus plantarum (strain ATCC BAA-793 / NCIMB 8826 / WCFS1) OX=220668 GN=lp_2903 PE=4 SV=1	0.547766571	0.006943	down
F9UNG6	lp_1416	Phosphoesterase OS=Lactobacillus plantarum (strain ATCC BAA-793 / NCIMB 8826 / WCFS1) OX=220668 GN=lp_1416 PE=4 SV=1	0.554909091	0.000676	down
F9UUC0	pepO	Endopeptidase PepO OS=Lactobacillus plantarum (strain ATCC BAA-793 / NCIMB 8826 / WCFS1) OX=220668 GN=pepO PE=4 SV=1	1.565060241	0.0001023	up
F9UNC5	lp_1360	Transcription regulator, MarR family OS=Lactobacillus plantarum (strain ATCC BAA-793 / NCIMB 8826 / WCFS1) OX=220668 GN=lp_1360 PE=4 SV=1	1.588228141	0.0002589	up
F9UM15	Phosphotransferase enzyme family protein	Uncharacterized protein OS=Lactobacillus plantarum (strain ATCC BAA-793 / NCIMB 8826 / WCFS1) OX=220668 GN=lp_0793 PE=4 SV=1	1.548091971	0.004743	up
F9UMV6	lp_1156	Cell surface hydrolase, DUF915 family OS=Lactobacillus plantarum (strain ATCC BAA-793 / NCIMB 8826 / WCFS1) OX=220668 GN=lp_1156 PE=4 SV=1	0.576119403	0.00343	down
F9UR82	lp_2567	Transcription regulator, MerR family OS=Lactobacillus plantarum (strain ATCC BAA-793 / NCIMB 8826 / WCFS1) OX=220668 GN=lp_2567 PE=4 SV=1	0.669011858	0.0001862	down
F9UN83	lp_1310	Glycosyl transferase, group I OS=Lactobacillus plantarum (strain ATCC BAA-793 / NCIMB 8826 / WCFS1) OX=220668 GN=lp_1310 PE=4 SV=1	1.612623762	8.23E-05	up
F9UU98	XRE family transcriptional regulator	Uncharacterized protein OS=Lactobacillus plantarum (strain ATCC BAA-793 / NCIMB 8826 / WCFS1) OX=220668 GN=lp_3419 PE=4 SV=1	0.4955	0.0004319	down
Q88UH8	UPF0237 protein lp_2508	UPF0237 protein lp_2508 OS=Lactobacillus plantarum (strain ATCC BAA-793 / NCIMB 8826 / WCFS1) OX=220668 GN=lp_2508 PE=3 SV=2	1.610763454	3.38E-05	up
F9UQ76	Endopeptidase La	Endopeptidase La OS=Lactobacillus plantarum (strain ATCC BAA-793 / NCIMB 8826 / WCFS1) OX=220668 GN=lon PE=3 SV=1	0.639192399	6.21E-05	down
F9UR18	lp_2486	Mucus-binding protein, LPXTG-motif cell wall anchor OS=Lactobacillus plantarum (strain ATCC BAA-793 / NCIMB 8826 / WCFS1) OX=220668 GN=lp_2486 PE=4 SV=1	0.597602475	0.0004204	down
Q88X40	D-alanyl carrier protein 2	D-alanyl carrier protein 2 OS=Lactobacillus plantarum (strain ATCC BAA-793 / NCIMB 8826 / WCFS1) OX=220668 GN=dltC2 PE=3 SV=1	0.535315985	7.23E-05	down
F9UQB3	Cysteine desulfurase	Cysteine desulfurase OS=Lactobacillus plantarum (strain ATCC BAA-793 / NCIMB 8826 / WCFS1) OX=220668 GN=iscS PE=3 SV=1	2.643351678	2.42E-05	up
F9UL55	manR	Sigma54 activator, mannose PTS operon regulator OS=Lactobacillus plantarum (strain ATCC BAA-793 / NCIMB 8826 / WCFS1) OX=220668 GN=manR PE=4 SV=1	2.062874252	0.0002351	up
F9UMR2	Type I site-specific deoxyribonuclease	Uncharacterized protein OS=Lactobacillus plantarum (strain ATCC BAA-793 / NCIMB 8826 / WCFS1) OX=220668 GN=lp_1098 PE=4 SV=1	2.216981132	3.77E-05	up
F9USJ3	lp_3077	Extracellular protein OS=Lactobacillus plantarum (strain ATCC BAA-793 / NCIMB 8826 / WCFS1) OX=220668 GN=lp_3077 PE=4 SV=1	0.399109589	0.0003945	down
Q88S84	L-arabinose isomerase	L-arabinose isomerase OS=Lactobacillus plantarum (strain ATCC BAA-793 / NCIMB 8826 / WCFS1) OX=220668 GN=araA PE=3 SV=1	0.45491453	3.12E-05	down
F9USA9	lp_2977	Cell surface protein, CscA/DUF916 family OS=Lactobacillus plantarum (strain ATCC BAA-793 / NCIMB 8826 / WCFS1) OX=220668 GN=lp_2977 PE=4 SV=1	0.498311307	2.84E-06	down
F9ULY5	lp_0758	Diguanylate cyclase/phosphodiesterase, EAL domain OS=Lactobacillus plantarum (strain ATCC BAA-793 / NCIMB 8826 / WCFS1) OX=220668 GN=lp_0758 PE=4 SV=1	1.572462848	1.80E-05	up
F9UTU9	hicD1	L-2-hydroxyisocaproate dehydrogenase OS=Lactobacillus plantarum (strain ATCC BAA-793 / NCIMB 8826 / WCFS1) OX=220668 GN=hicD1 PE=3 SV=1	1.559493671	0.0003185	up
Q88S55	lp_3588	Transcription regulator, Rrf2 family OS=Lactobacillus plantarum (strain ATCC BAA-793 / NCIMB 8826 / WCFS1) OX=220668 GN=lp_3588 PE=4 SV=1	1.607741935	8.77E-05	up

F9UPD3	pgm5	Phosphoglycerate mutase family protein OS=Lactobacillus plantarum (strain ATCC BAA-793 / NCIMB 8826 / WCFS1) OX=220668 GN=pgm5 PE=4 SV=1	1.8738 40445	5.70E-0 5	up
F9URL9	lp_2745	Nucleotide-binding protein, universal stress protein UspA family OS=Lactobacillus plantarum (strain ATCC BAA-793 / NCIMB 8826 / WCFS1) OX=220668 GN=lp_2745 PE=3 SV=1	0.5730 98039	2.84E-0 6	down
F9UM41	lp_0823	Diguanylate cyclase/phosphodiesterase, EAL domain OS=Lactobacillus plantarum (strain ATCC BAA-793 / NCIMB 8826 / WCFS1) OX=220668 GN=lp_0823 PE=4 SV=1	1.5928 85889	0.00160 5	up
Q88VY8	Segregati on and condensat ion protein B	Segregation and condensation protein B OS=Lactobacillus plantarum (strain ATCC BAA-793 / NCIMB 8826 / WCFS1) OX=220668 GN=scpB PE=3 SV=1	2.5171 86674	0.00815 7	up
F9UN46	lp_1256	Extracellular protein OS=Lactobacillus plantarum (strain ATCC BAA-793 / NCIMB 8826 / WCFS1) OX=220668 GN=lp_1256 PE=4 SV=1	0.6394 56343	0.00894 2	down
F9UUG8	lp_3502	Transcription regulator, LysR family OS=Lactobacillus plantarum (strain ATCC BAA-793 / NCIMB 8826 / WCFS1) OX=220668 GN=lp_3502 PE=3 SV=1	1.8326 92034	0.00202	up
Q88V55	Global transcripti onal regulator Spx	Global transcriptional regulator Spx OS=Lactobacillus plantarum (strain ATCC BAA-793 / NCIMB 8826 / WCFS1) OX=220668 GN=spx PE=3 SV=1	0.4666 42066	2.48E-0 5	down
Q88VQ3	D-aminoa cyl-tRNA deacylase	D-aminoacyl-tRNA deacylase OS=Lactobacillus plantarum (strain ATCC BAA-793 / NCIMB 8826 / WCFS1) OX=220668 GN=dtd PE=3 SV=1	1.5344 38776	0.00035 19	up
F9UL70	lp_0603	Acetyltransferase, GNAT family OS=Lactobacillus plantarum (strain ATCC BAA-793 / NCIMB 8826 / WCFS1) OX=220668 GN=lp_0603 PE=4 SV=1	1.5633 07494	0.00113 7	up
F9UPF1	DUF960 domain-c ontaining protein	Uncharacterized protein OS=Lactobacillus plantarum (strain ATCC BAA-793 / NCIMB 8826 / WCFS1) OX=220668 GN=lp_1806 PE=4 SV=1	2.1814 08088	0.00034 71	up
F9UKZ6	DEAD-bo x ATP-depe ndent RNA helicase CshA	DEAD-box ATP-dependent RNA helicase CshA OS=Lactobacillus plantarum (strain ATCC BAA-793 / NCIMB 8826 / WCFS1) OX=220668 GN=rhe1 PE=3 SV=1	1.6829 93197	0.00115 1	up
F9URK6	lp_2732	NADPH-dependent FMN reductase family protein OS=Lactobacillus plantarum (strain ATCC BAA-793 / NCIMB 8826 / WCFS1) OX=220668 GN=lp_2732 PE=4 SV=1	1.8804 04514	5.37E-0 7	up
F9USX8	lp_0165	Transcription regulator, TetR family OS=Lactobacillus plantarum (strain ATCC BAA-793 / NCIMB 8826 / WCFS1) OX=220668 GN=lp_0165 PE=4 SV=1	1.5226 07239	0.00056 31	up
F9UUC2	nox5	NADH oxidase OS=Lactobacillus plantarum (strain ATCC BAA-793 / NCIMB 8826 / WCFS1) OX=220668 GN=nox5 PE=4 SV=1	1.5456 79656	0.00065 05	up
F9UPK5	lp_1876	Hydrolase, HAD superfamily, Cof family OS=Lactobacillus plantarum (strain ATCC BAA-793 / NCIMB 8826 / WCFS1) OX=220668 GN=lp_1876 PE=4 SV=1	1.9185 26284	6.54E-0 5	up
F9USK0	asnB2	Asparagine synthase (Glutamine-hydrolysing) OS=Lactobacillus plantarum (strain ATCC BAA-793 / NCIMB 8826 / WCFS1) OX=220668 GN=asnB2 PE=3 SV=1	1.7268 90756	7.20E-0 5	up
F9UTE3	lp_3244	NADPH-dependent FMN reductase family protein OS=Lactobacillus plantarum (strain ATCC BAA-793 / NCIMB 8826 / WCFS1) OX=220668 GN=lp_3244 PE=4 SV=1	2.1125 85959	2.87E-0 5	up
F9URF7	Lipocalin-like domain-c ontaining protein	Uncharacterized protein OS=Lactobacillus plantarum (strain ATCC BAA-793 / NCIMB 8826 / WCFS1) OX=220668 GN=lp_2667 PE=4 SV=1	1.6650 67836	2.52E-0 5	up
F9UU18	DNA helicase	DNA helicase OS=Lactobacillus plantarum (strain ATCC BAA-793 / NCIMB 8826 / WCFS1) OX=220668 GN=lp_0432 PE=4 SV=1	1.6060 64672	6.38E-0 5	up
F9UTP0	Transglyc osylase	Uncharacterized protein OS=Lactobacillus plantarum (strain ATCC BAA-793 / NCIMB 8826 / WCFS1) OX=220668 GN=lp_0284 PE=4 SV=1	2.5309 09091	3.34E-0 5	up
Q88SC3	N-acetyl muramic acid 6-phosph	N-acetylmuramic acid 6-phosphate etherase 1 OS=Lactobacillus plantarum (strain ATCC BAA-793 / NCIMB 8826 / WCFS1) OX=220668 GN=murQ1 PE=3 SV=2	1.6437 4234	0.00013 42	up

	ate etherase 1				
Q88VE9	UvrABC system protein C	UvrABC system protein C OS=Lactobacillus plantarum (strain ATCC BAA-793 / NCIMB 8826 / WCFS1) OX=220668 GN=uvrC PE=3 SV=1	1.7535 14128	0.00011 73	up
F9UUC6	lp_3453	Cell surface protein, CscB family OS=Lactobacillus plantarum (strain ATCC BAA-793 / NCIMB 8826 / WCFS1) OX=220668 GN=lp_3453 PE=4 SV=1	0.2912 49165	0.00020 48	down
F9UPS7	RNA polymera se sigma factor SigA	RNA polymerase sigma factor SigA OS=Lactobacillus plantarum (strain ATCC BAA-793 / NCIMB 8826 / WCFS1) OX=220668 GN=rpoD PE=3 SV=1	1.5576 48932	2.04E-0 7	up
Q88WJ3	Ribosome maturatio n factor RimM	Ribosome maturation factor RimM OS=Lactobacillus plantarum (strain ATCC BAA-793 / NCIMB 8826 / WCFS1) OX=220668 GN=rimM PE=3 SV=1	1.7785 47712	5.80E-0 6	up
F9UQU6	lp_2406	Prophage P2a protein 51 OS=Lactobacillus plantarum (strain ATCC BAA-793 / NCIMB 8826 / WCFS1) OX=220668 GN=lp_2406 PE=4 SV=1	0.5396 31705	4.34E-0 5	down
F9UU51	Bacterioci n immunity protein	Uncharacterized protein OS=Lactobacillus plantarum (strain ATCC BAA-793 / NCIMB 8826 / WCFS1) OX=220668 GN=lp_3366 PE=4 SV=1	1.9331 59589	0.00020 54	up
F9UR83	lp_2568	Short-chain dehydrogenase/oxidoreductase OS=Lactobacillus plantarum (strain ATCC BAA-793 / NCIMB 8826 / WCFS1) OX=220668 GN=lp_2568 PE=3 SV=1	0.6388 12392	0.00095 1	down
F9UPE3	lp_1796	DegV family protein OS=Lactobacillus plantarum (strain ATCC BAA-793 / NCIMB 8826 / WCFS1) OX=220668 GN=lp_1796 PE=4 SV=1	1.9946 51565	3.40E-0 5	up
F9UUB4	Thioredox in	Thioredoxin OS=Lactobacillus plantarum (strain ATCC BAA-793 / NCIMB 8826 / WCFS1) OX=220668 GN=trxA3 PE=3 SV=1	1.5387 69518	0.00030 87	up
F9UST5	lp_0111	Medium chain dehydrogenases/reductase (MDR)/zinc-dependent alcohol dehydrogenase-like family protein OS=Lactobacillus plantarum (strain ATCC BAA-793 / NCIMB 8826 / WCFS1) OX=220668 GN=lp_0111 PE=4 SV=1	1.6114 39571	1.09E-0 5	up
F9UMI8	lp_1003	Acetyltransferase, GNAT family OS=Lactobacillus plantarum (strain ATCC BAA-793 / NCIMB 8826 / WCFS1) OX=220668 GN=lp_1003 PE=4 SV=1	2.3682 81001	5.41E-0 5	up
Q88V04	S-adenosy lmethioni ne:tRNA ribosyltra nsferase-i somerase	S-adenosylmethionine:tRNA ribosyltransferase-isomerase OS=Lactobacillus plantarum (strain ATCC BAA-793 / NCIMB 8826 / WCFS1) OX=220668 GN=queA PE=3 SV=1	1.6577 84011	6.86E-0 5	up
F9UTD6	lp_3236	Short-chain dehydrogenase/oxidoreductase,atypical SDR family, TMR-like OS=Lactobacillus plantarum (strain ATCC BAA-793 / NCIMB 8826 / WCFS1) OX=220668 GN=lp_3236 PE=4 SV=1	1.6770 53824	0.00014 44	up
F9UPK1	lp_1871	ABC transporter, ATP-binding protein, ChvD family OS=Lactobacillus plantarum (strain ATCC BAA-793 / NCIMB 8826 / WCFS1) OX=220668 GN=lp_1871 PE=4 SV=1	1.8863 60165	9.36E-0 6	up
F9UPG9	lp_1833	Integrase, N-terminal SAM-like OS=Lactobacillus plantarum (strain ATCC BAA-793 / NCIMB 8826 / WCFS1) OX=220668 GN=lp_1833 PE=4 SV=1	1.7559 04357	1.14E-0 5	up
F9UTA7	parB1	Chomosome partitioning protein, DNA-binding protein OS=Lactobacillus plantarum (strain ATCC BAA-793 / NCIMB 8826 / WCFS1) OX=220668 GN=parB1 PE=3 SV=1	1.5421 13115	1.97E-0 5	up
F9UP88	Phospho mevalona te kinase	Phosphomevalonate kinase OS=Lactobacillus plantarum (strain ATCC BAA-793 / NCIMB 8826 / WCFS1) OX=220668 GN=mvaK2 PE=4 SV=1	1.8806 7046	4.76E-0 5	up
F9UTG5	ptp2	Protein-tyrosine phosphatase OS=Lactobacillus plantarum (strain ATCC BAA-793 / NCIMB 8826 / WCFS1) OX=220668 GN=ptp2 PE=4 SV=1	1.6867 81609	0.00012 94	up
F9UTF9	glyK	Glycerate kinase OS=Lactobacillus plantarum (strain ATCC BAA-793 / NCIMB 8826 / WCFS1) OX=220668 GN=glyK PE=3 SV=1	2.6849 09776	6.64E-0 5	up
F9UPQ9	lp_1938	Transcription regulator, LysR family OS=Lactobacillus plantarum (strain ATCC BAA-793 / NCIMB 8826 / WCFS1) OX=220668 GN=lp_1938 PE=3 SV=1	1.5135 9394	1.26E-0 5	up
Q88S59	Putative AgrB-like protein	Putative AgrB-like protein OS=Lactobacillus plantarum (strain ATCC BAA-793 / NCIMB 8826 / WCFS1) OX=220668 GN=lp_3582 PE=3 SV=1	2.0204 97804	0.00060 4	up
F9URA0	lp_2591	Purine nucleosidase OS=Lactobacillus plantarum (strain ATCC BAA-793 / NCIMB 8826 / WCFS1) OX=220668 GN=lp_2591 PE=4 SV=1	1.6046 60957	0.00021 33	up

F9UL33	nagA	N-acetylglucosamine-6-phosphate deacetylase OS=Lactobacillus plantarum (strain ATCC BAA-793 / NCIMB 8826 / WCFS1) OX=220668 GN=nagA PE=3 SV=1	1.50879567	6.49E-05	up
F9UNL1	sufU	SUF system FeS assembly protein, NifU family OS=Lactobacillus plantarum (strain ATCC BAA-793 / NCIMB 8826 / WCFS1) OX=220668 GN=sufU PE=4 SV=1	1.656872581	5.08E-05	up
F9UNC9	lp_1369	Lipoprotein OS=Lactobacillus plantarum (strain ATCC BAA-793 / NCIMB 8826 / WCFS1) OX=220668 GN=lp_1369 PE=4 SV=1	0.55530303	0.009328	down
F9URK8	Lipoate--protein ligase	Lipoate--protein ligase OS=Lactobacillus plantarum (strain ATCC BAA-793 / NCIMB 8826 / WCFS1) OX=220668 GN=lplA2 PE=4 SV=1	1.811263318	3.89E-06	up
F9URK7	lp_2733	NADPH-dependent FMN reductase family protein OS=Lactobacillus plantarum (strain ATCC BAA-793 / NCIMB 8826 / WCFS1) OX=220668 GN=lp_2733 PE=4 SV=1	1.802130898	8.48E-06	up
F9UR15	Triple QxxK/R motif-containing protein	Uncharacterized protein OS=Lactobacillus plantarum (strain ATCC BAA-793 / NCIMB 8826 / WCFS1) OX=220668 GN=lp_2483 PE=4 SV=1	0.580915371	0.004995	down
F9UMD8	lp_0941	Phage integrase OS=Lactobacillus plantarum (strain ATCC BAA-793 / NCIMB 8826 / WCFS1) OX=220668 GN=lp_0941 PE=4 SV=1	1.61018161	0.0004484	up
F9UTE4	Condensation domain-containing protein	Uncharacterized protein OS=Lactobacillus plantarum (strain ATCC BAA-793 / NCIMB 8826 / WCFS1) OX=220668 GN=lp_3245 PE=4 SV=1	1.661313869	2.89E-06	up
Q88VK3	Ribosome maturation factor RimP	Ribosome maturation factor RimP OS=Lactobacillus plantarum (strain ATCC BAA-793 / NCIMB 8826 / WCFS1) OX=220668 GN=rimP PE=3 SV=1	2.120952544	3.16E-05	up
F9URN4	lp_2763	Nucleotide-binding protein, histidine triad family OS=Lactobacillus plantarum (strain ATCC BAA-793 / NCIMB 8826 / WCFS1) OX=220668 GN=lp_2763 PE=4 SV=1	1.681885125	2.29E-06	up
F9UT07	lp_0200	ABC transporter, substrate binding protein OS=Lactobacillus plantarum (strain ATCC BAA-793 / NCIMB 8826 / WCFS1) OX=220668 GN=lp_0200 PE=3 SV=1	1.583132188	0.000575	up
F9USD4	lp_3006	Transcription regulator, TetR family OS=Lactobacillus plantarum (strain ATCC BAA-793 / NCIMB 8826 / WCFS1) OX=220668 GN=lp_3006 PE=4 SV=1	2.693089431	1.62E-06	up
F9UNH5	lp_1427	Nucleoside 2-deoxyribosyltransferase OS=Lactobacillus plantarum (strain ATCC BAA-793 / NCIMB 8826 / WCFS1) OX=220668 GN=lp_1427 PE=4 SV=1	1.701652523	0.001004	up
Q88W33	Peptide methionine sulfoxide reductase MsrB	Peptide methionine sulfoxide reductase MsrB OS=Lactobacillus plantarum (strain ATCC BAA-793 / NCIMB 8826 / WCFS1) OX=220668 GN=msrB PE=3 SV=1	1.600974631	0.0002458	up
F9ULV1	gpp	Glycoprotein endopeptidase, M22 family OS=Lactobacillus plantarum (strain ATCC BAA-793 / NCIMB 8826 / WCFS1) OX=220668 GN=gpp PE=4 SV=1	1.614019905	1.53E-05	up
Q88YZ2	Acetyl-coenzyme A carboxylase carboxyl transferase subunit beta 1	Acetyl-coenzyme A carboxylase carboxyl transferase subunit beta 1 OS=Lactobacillus plantarum (strain ATCC BAA-793 / NCIMB 8826 / WCFS1) OX=220668 GN=accD1 PE=3 SV=1	1.99073308	1.11E-05	up
F9UKW0	lp_0477	Lipoate-protein ligase A OS=Lactobacillus plantarum (strain ATCC BAA-793 / NCIMB 8826 / WCFS1) OX=220668 GN=lp_0477 PE=4 SV=1	1.851969002	0.0006351	up
F9UQA4	TPR_REGION domain-containing protein	Uncharacterized protein OS=Lactobacillus plantarum (strain ATCC BAA-793 / NCIMB 8826 / WCFS1) OX=220668 GN=lp_2169 PE=4 SV=1	1.688796061	5.65E-07	up
F9UT57	DUF2089 family protein	Uncharacterized protein OS=Lactobacillus plantarum (strain ATCC BAA-793 / NCIMB 8826 / WCFS1) OX=220668 GN=lp_0260 PE=4 SV=1	1.619186047	2.19E-06	up
F9URL0	cah	Carbonate dehydratase OS=Lactobacillus plantarum (strain ATCC BAA-793 / NCIMB 8826 / WCFS1) OX=220668 GN=cah PE=4 SV=1	1.584927369	0.0001845	up

F9USH2	lp_3050	Extracellular transglycosylase, membrane-bound OS=Lactobacillus plantarum (strain ATCC BAA-793 / NCIMB 8826 / WCFS1) OX=220668 GN=lp_3050 PE=4 SV=1	0.517989865	0.006362	down
F9UMF8	Pseudouridine synthase	Pseudouridine synthase OS=Lactobacillus plantarum (strain ATCC BAA-793 / NCIMB 8826 / WCFS1) OX=220668 GN=rluE PE=3 SV=1	1.517444304	1.40E-05	up
F9UM08	rpoN	DNA-directed RNA polymerase, sigma factor 54 OS=Lactobacillus plantarum (strain ATCC BAA-793 / NCIMB 8826 / WCFS1) OX=220668 GN=rpoN PE=3 SV=1	1.648238243	1.20E-05	up
F9UMN4	hepB1	Heptaprenyl diphosphate synthase component II OS=Lactobacillus plantarum (strain ATCC BAA-793 / NCIMB 8826 / WCFS1) OX=220668 GN=hepB1 PE=3 SV=1	1.776712541	9.09E-06	up
F9UP74	Diaminopimelate decarboxylase	Diaminopimelate decarboxylase OS=Lactobacillus plantarum (strain ATCC BAA-793 / NCIMB 8826 / WCFS1) OX=220668 GN=lysA PE=3 SV=1	2.293005324	1.49E-07	up
F9URM9	thrC	Threonine synthase OS=Lactobacillus plantarum (strain ATCC BAA-793 / NCIMB 8826 / WCFS1) OX=220668 GN=thrC PE=3 SV=1	1.504189944	2.16E-07	up
F9UTN9	rrp2	Two-component system response regulator OS=Lactobacillus plantarum (strain ATCC BAA-793 / NCIMB 8826 / WCFS1) OX=220668 GN=rrp2 PE=4 SV=1	2.080247977	8.04E-05	up
F9UP79	gabT	4-aminobutyrate aminotransferase OS=Lactobacillus plantarum (strain ATCC BAA-793 / NCIMB 8826 / WCFS1) OX=220668 GN=gabT PE=3 SV=1	1.906621116	0.0002017	up
F9UMU5	ATP-dependent DNA helicase	ATP-dependent DNA helicase OS=Lactobacillus plantarum (strain ATCC BAA-793 / NCIMB 8826 / WCFS1) OX=220668 GN=pcrA PE=3 SV=1	1.612761598	6.82E-05	up
F9UQE3	lp_2219	6-phosphogluconolactonase OS=Lactobacillus plantarum (strain ATCC BAA-793 / NCIMB 8826 / WCFS1) OX=220668 GN=lp_2219 PE=1 SV=1	1.513445023	5.46E-05	up
Q88VR2	Probable endonuclease 4	Probable endonuclease 4 OS=Lactobacillus plantarum (strain ATCC BAA-793 / NCIMB 8826 / WCFS1) OX=220668 GN=nfo PE=3 SV=1	1.561151079	5.62E-06	up
F9UL78	lp_0613	Nuclease, NYN_YacP family OS=Lactobacillus plantarum (strain ATCC BAA-793 / NCIMB 8826 / WCFS1) OX=220668 GN=lp_0613 PE=4 SV=1	1.677448473	4.65E-05	up
F9UQ20	Extracellular protein	Uncharacterized protein OS=Lactobacillus plantarum (strain ATCC BAA-793 / NCIMB 8826 / WCFS1) OX=220668 GN=lp_2066 PE=4 SV=1	1.570953276	7.21E-05	up
F9UN85	tagE3	Poly(Glycerol-phosphate) alpha-glucosyltransferase OS=Lactobacillus plantarum (strain ATCC BAA-793 / NCIMB 8826 / WCFS1) OX=220668 GN=tagE3 PE=4 SV=1	1.871666397	9.17E-06	up
F9UQ86	50S ribosomal subunit assembly factor BipA	50S ribosomal subunit assembly factor BipA OS=Lactobacillus plantarum (strain ATCC BAA-793 / NCIMB 8826 / WCFS1) OX=220668 GN=typA PE=3 SV=1	1.780178488	1.42E-05	up
F9UU35	DUF72 domain-containing protein	Uncharacterized protein OS=Lactobacillus plantarum (strain ATCC BAA-793 / NCIMB 8826 / WCFS1) OX=220668 GN=lp_0450 PE=4 SV=1	1.517005835	5.49E-05	up
F9UP42	accC2	Acetyl-CoA carboxylase, biotin carboxylase subunit OS=Lactobacillus plantarum (strain ATCC BAA-793 / NCIMB 8826 / WCFS1) OX=220668 GN=accC2 PE=4 SV=1	1.806349206	1.14E-06	up
F9UQJ1	zapA	Cell-division Z-ring component, stimulator of FtsZ polymerization OS=Lactobacillus plantarum (strain ATCC BAA-793 / NCIMB 8826 / WCFS1) OX=220668 GN=zapA PE=4 SV=1	1.729195615	0.0001519	up
F9US18	nrdD	Anaerobic ribonucleoside-triphosphate reductase OS=Lactobacillus plantarum (strain ATCC BAA-793 / NCIMB 8826 / WCFS1) OX=220668 GN=nrdD PE=4 SV=1	1.726052753	2.33E-05	up
F9UTQ1	tag1	DNA-3-methyladenine glycosylase I OS=Lactobacillus plantarum (strain ATCC BAA-793 / NCIMB 8826 / WCFS1) OX=220668 GN=tag1 PE=4 SV=1	2.280669145	3.72E-05	up
F9USE0	lp_3013	Transcription regulator, MerR family OS=Lactobacillus plantarum (strain ATCC BAA-793 / NCIMB 8826 / WCFS1) OX=220668 GN=lp_3013 PE=4 SV=1	1.700596969	5.87E-05	up
F9UQT2	Branched-chain-amino-acid transaminase	Branched-chain-amino-acid transaminase OS=Lactobacillus plantarum (strain ATCC BAA-793 / NCIMB 8826 / WCFS1) OX=220668 GN=bcaT PE=3 SV=1	1.522268366	1.61E-06	up

Q88WG0	Acetyl-coenzyme A carboxylase carboxyl transferase subunit beta 2	Acetyl-coenzyme A carboxylase carboxyl transferase subunit beta 2 OS=Lactobacillus plantarum (strain ATCC BAA-793 / NCIMB 8826 / WCFS1) OX=220668 GN=accD2 PE=3 SV=1	1.558346721	4.83E-05	up
F9UM23	glnQ1	Glutamine ABC transporter, ATP-binding protein OS=Lactobacillus plantarum (strain ATCC BAA-793 / NCIMB 8826 / WCFS1) OX=220668 GN=glnQ1 PE=4 SV=1	1.625393967	4.25E-05	up
Q88UZ8	DNA mismatch repair protein MutL	DNA mismatch repair protein MutL OS=Lactobacillus plantarum (strain ATCC BAA-793 / NCIMB 8826 / WCFS1) OX=220668 GN=mutL PE=3 SV=1	2.107886772	0.0006735	up
F9URW4	Signal peptidase I	Signal peptidase I OS=Lactobacillus plantarum (strain ATCC BAA-793 / NCIMB 8826 / WCFS1) OX=220668 GN=sip2 PE=3 SV=1	0.357976654	0.003268	down
F9UTE7	lp_3248	Bacteriocin immunity protein OS=Lactobacillus plantarum (strain ATCC BAA-793 / NCIMB 8826 / WCFS1) OX=220668 GN=lp_3248 PE=4 SV=1	2.697033898	8.25E-06	up
Q88RX6	tRNA uridine 5-carboxymethylaminomethyl modification enzyme MnmG	tRNA uridine 5-carboxymethylaminomethyl modification enzyme MnmG OS=Lactobacillus plantarum (strain ATCC BAA-793 / NCIMB 8826 / WCFS1) OX=220668 GN=mnmG PE=3 SV=1	1.513622603	8.24E-05	up
F9UM44	Maltose epimerase	Maltose epimerase OS=Lactobacillus plantarum (strain ATCC BAA-793 / NCIMB 8826 / WCFS1) OX=220668 GN=galM1 PE=3 SV=1	1.744131086	1.56E-05	up
F9UR54	pts18CBA	PTS system, N-acetylglucosamine and glucose-specific EIICBA component OS=Lactobacillus plantarum (strain ATCC BAA-793 / NCIMB 8826 / WCFS1) OX=220668 GN=pts18CBA PE=4 SV=1	1.692736565	0.0003135	up
F9UTE1	nth2	Endonuclease III OS=Lactobacillus plantarum (strain ATCC BAA-793 / NCIMB 8826 / WCFS1) OX=220668 GN=nth2 PE=4 SV=1	1.83776726	2.22E-06	up
F9UME9	lp_0954	Transcription regulator, MarR family OS=Lactobacillus plantarum (strain ATCC BAA-793 / NCIMB 8826 / WCFS1) OX=220668 GN=lp_0954 PE=4 SV=1	1.546134663	0.0003881	up
F9UPP0	lp_1918	NAD(P)(H)-dependent oxidoreductase, quinone oxidoreductase (QOR) family OS=Lactobacillus plantarum (strain ATCC BAA-793 / NCIMB 8826 / WCFS1) OX=220668 GN=lp_1918 PE=4 SV=1	1.559036501	0.0001111	up
F9UMR1	mtsA	Manganese/zinc ABC transporter, substrate binding protein OS=Lactobacillus plantarum (strain ATCC BAA-793 / NCIMB 8826 / WCFS1) OX=220668 GN=mtsA PE=3 SV=1	0.665992293	0.001742	down
F9UQN3	csd2	Cysteine desulfurase OS=Lactobacillus plantarum (strain ATCC BAA-793 / NCIMB 8826 / WCFS1) OX=220668 GN=csd2 PE=3 SV=1	1.62043353	3.00E-06	up
F9UQD9	rrmA	rRNA large subunit methyltransferase A OS=Lactobacillus plantarum (strain ATCC BAA-793 / NCIMB 8826 / WCFS1) OX=220668 GN=rrmA PE=4 SV=1	1.573619175	3.75E-06	up
F9UT65	tagF1	CDP-glycerol glycerophosphotransferase OS=Lactobacillus plantarum (strain ATCC BAA-793 / NCIMB 8826 / WCFS1) OX=220668 GN=tagF1 PE=3 SV=1	1.541181671	1.86E-05	up
F9UP00	ATP-dependent DNA helicase RecG	ATP-dependent DNA helicase RecG OS=Lactobacillus plantarum (strain ATCC BAA-793 / NCIMB 8826 / WCFS1) OX=220668 GN=recG PE=3 SV=1	1.544931386	5.71E-05	up
F9UP96	dnaD	DNA replication protein DnaD OS=Lactobacillus plantarum (strain ATCC BAA-793 / NCIMB 8826 / WCFS1) OX=220668 GN=dnaD PE=4 SV=1	1.543455806	8.81E-05	up
F9UU33	IpaB/EvcA family protein	Uncharacterized protein OS=Lactobacillus plantarum (strain ATCC BAA-793 / NCIMB 8826 / WCFS1) OX=220668 GN=lp_0448 PE=4 SV=1	1.510204082	1.57E-05	up
Q88YI8	UvrABC system protein B	UvrABC system protein B OS=Lactobacillus plantarum (strain ATCC BAA-793 / NCIMB 8826 / WCFS1) OX=220668 GN=uvrB PE=3 SV=1	1.6828125	7.76E-05	up
F9UPH5	DNA topoisom	DNA topoisomerase 4 subunit A OS=Lactobacillus plantarum (strain ATCC BAA-793 / NCIMB 8826 / WCFS1) OX=220668 GN=parC PE=3	1.708510974	7.08E-06	up

	erase 4 subunit A	SV=1			
F9UNZ1	16S rRNA (cytosine(967)-C(5))-methyltransferase	16S rRNA m5C967 methyltransferase OS=Lactobacillus plantarum (strain ATCC BAA-793 / NCIMB 8826 / WCFS1) OX=220668 GN=sunL PE=3 SV=1	1.682110884	6.49E-07	up
F9UQ00	Transcription termination/antitermination protein NusA	Transcription termination/antitermination protein NusA OS=Lactobacillus plantarum (strain ATCC BAA-793 / NCIMB 8826 / WCFS1) OX=220668 GN=nusA PE=3 SV=1	1.553092706	8.80E-08	up
F9UPD7	DUF2087 domain-containing protein	DUF2087 domain-containing protein OS=Lactobacillus plantarum (strain ATCC BAA-793 / NCIMB 8826 / WCFS1) OX=220668 GN=lp_1789 PE=4 SV=1	1.65131887	0.0007096	up
F9UN91	Pseudouridine synthase	Pseudouridine synthase OS=Lactobacillus plantarum (strain ATCC BAA-793 / NCIMB 8826 / WCFS1) OX=220668 GN=rsuA PE=3 SV=1	2.018837747	2.63E-06	up
Q88WP5	tRNA dimethylallyltransferase	tRNA dimethylallyltransferase OS=Lactobacillus plantarum (strain ATCC BAA-793 / NCIMB 8826 / WCFS1) OX=220668 GN=miaA PE=3 SV=1	1.514747295	2.91E-05	up
F9UM35	lp_0816	Transcription regulator, MarR family OS=Lactobacillus plantarum (strain ATCC BAA-793 / NCIMB 8826 / WCFS1) OX=220668 GN=lp_0816 PE=4 SV=1	1.879251701	5.54E-06	up
Q88UZ7	DNA mismatch repair protein MutS	DNA mismatch repair protein MutS OS=Lactobacillus plantarum (strain ATCC BAA-793 / NCIMB 8826 / WCFS1) OX=220668 GN=mutS PE=3 SV=1	1.957015551	6.75E-07	up
Q88WM7	Exodeoxyribonuclease 7 large subunit	Exodeoxyribonuclease 7 large subunit OS=Lactobacillus plantarum (strain ATCC BAA-793 / NCIMB 8826 / WCFS1) OX=220668 GN=xseA PE=3 SV=1	1.610313417	1.94E-05	up
F9US91	lp_0073	Glutamine amidotransferase class-I OS=Lactobacillus plantarum (strain ATCC BAA-793 / NCIMB 8826 / WCFS1) OX=220668 GN=lp_0073 PE=4 SV=1	1.592200215	8.52E-06	up
F9UQJ8	lp_2279	Phosphoesterase, DHH family OS=Lactobacillus plantarum (strain ATCC BAA-793 / NCIMB 8826 / WCFS1) OX=220668 GN=lp_2279 PE=4 SV=1	1.621873544	1.94E-06	up
F9UL13	Transcription-repair-coupling factor	Transcription-repair-coupling factor OS=Lactobacillus plantarum (strain ATCC BAA-793 / NCIMB 8826 / WCFS1) OX=220668 GN=mfd PE=3 SV=1	1.551384895	0.0001103	up
F9ULV5	lp_0723	ABC transporter, ATP-binding protein OS=Lactobacillus plantarum (strain ATCC BAA-793 / NCIMB 8826 / WCFS1) OX=220668 GN=lp_0723 PE=4 SV=1	2.15851602	3.00E-06	up
F9UNT0	sbcC	Exonuclease SbcC OS=Lactobacillus plantarum (strain ATCC BAA-793 / NCIMB 8826 / WCFS1) OX=220668 GN=sbcC PE=4 SV=1	1.734611012	2.76E-06	up
Q88VG6	Ribonuclease Z	Ribonuclease Z OS=Lactobacillus plantarum (strain ATCC BAA-793 / NCIMB 8826 / WCFS1) OX=220668 GN=rnz PE=3 SV=1	1.524838661	7.20E-06	up
F9URQ1	lp_2783	Glycosyltransferase, family 2 OS=Lactobacillus plantarum (strain ATCC BAA-793 / NCIMB 8826 / WCFS1) OX=220668 GN=lp_2783 PE=4 SV=1	1.627362174	0.0006285	up
Q88V20	dITP/XTP pyrophosphatase	dITP/XTP pyrophosphatase OS=Lactobacillus plantarum (strain ATCC BAA-793 / NCIMB 8826 / WCFS1) OX=220668 GN=lp_2267 PE=3 SV=1	1.786839052	6.42E-07	up
Q88XF3	Peptide chain release factor 3	Peptide chain release factor 3 OS=Lactobacillus plantarum (strain ATCC BAA-793 / NCIMB 8826 / WCFS1) OX=220668 GN=prfC PE=3 SV=2	1.525627536	8.62E-06	up
F9URP6	pbg4	6-phospho-beta-glucosidase OS=Lactobacillus plantarum (strain ATCC BAA-793 / NCIMB 8826 / WCFS1) OX=220668 GN=pbg4 PE=3 SV=1	1.659015355	5.76E-05	up
F9UPI1	Tyrosine recombinase XerC	Tyrosine recombinase XerC OS=Lactobacillus plantarum (strain ATCC BAA-793 / NCIMB 8826 / WCFS1) OX=220668 GN=xerC PE=3 SV=1	1.648264984	6.33E-06	up
F9UTF3	lp_3256	DegV family protein OS=Lactobacillus plantarum (strain ATCC BAA-793 / NCIMB 8826 / WCFS1) OX=220668 GN=lp_3256 PE=4 SV=1	1.754472345	4.01E-06	up

F9URG5	lp_2677	Medium chain degydrogenase/reductase, MDR family OS=Lactobacillus plantarum (strain ATCC BAA-793 / NCIMB 8826 / WCFS1) OX=220668 GN=lp_2677 PE=4 SV=1	1.845005935	9.65E-05	up
F9UQP2	lp_2337	ATPase, AAA family OS=Lactobacillus plantarum (strain ATCC BAA-793 / NCIMB 8826 / WCFS1) OX=220668 GN=lp_2337 PE=3 SV=1	1.557114534	2.45E-06	up
P96349	Cold shock protein 2	Cold shock protein 2 OS=Lactobacillus plantarum (strain ATCC BAA-793 / NCIMB 8826 / WCFS1) OX=220668 GN=cspL PE=2 SV=1	0.576387582	0.006438	down
F9ULA4	lp_0641	Prophage P1 protein 18, DNA single-strand annealing protein RecT OS=Lactobacillus plantarum (strain ATCC BAA-793 / NCIMB 8826 / WCFS1) OX=220668 GN=lp_0641 PE=4 SV=1	0.621726479	0.004609	down
F9UNA0	dgk2	Deoxynucleoside kinase OS=Lactobacillus plantarum (strain ATCC BAA-793 / NCIMB 8826 / WCFS1) OX=220668 GN=dgk2 PE=3 SV=1	1.794314381	1.15E-05	up
F9URB3	lp_2606	NAD(P)-dependent oxidoreductase OS=Lactobacillus plantarum (strain ATCC BAA-793 / NCIMB 8826 / WCFS1) OX=220668 GN=lp_2606 PE=4 SV=1	1.658443419	8.25E-05	up
F9UQC0	lp_2190	Cell division protein, RNA-binding S4 domain-containing protein OS=Lactobacillus plantarum (strain ATCC BAA-793 / NCIMB 8826 / WCFS1) OX=220668 GN=lp_2190 PE=4 SV=1	1.553650696	0.000337	up
F9URJ0	NmrA domain-containing protein	NmrA domain-containing protein OS=Lactobacillus plantarum (strain ATCC BAA-793 / NCIMB 8826 / WCFS1) OX=220668 GN=lp_2713 PE=4 SV=1	1.542691309	0.0001253	up
F9USW7	lp_0152	Transcription regulator, GntR family OS=Lactobacillus plantarum (strain ATCC BAA-793 / NCIMB 8826 / WCFS1) OX=220668 GN=lp_0152 PE=4 SV=1	1.560862866	7.95E-06	up
Q88SI6	Demethylmenaquinone methyltransferase	Demethylmenaquinone methyltransferase OS=Lactobacillus plantarum (strain ATCC BAA-793 / NCIMB 8826 / WCFS1) OX=220668 GN=menG PE=3 SV=1	1.863793103	7.12E-07	up
F9UQA3	ATP-dependent RecD-like DNA helicase	ATP-dependent RecD-like DNA helicase OS=Lactobacillus plantarum (strain ATCC BAA-793 / NCIMB 8826 / WCFS1) OX=220668 GN=recD PE=3 SV=1	1.625	1.40E-05	up
F9UQG0	lp_2235	Acid sugar phosphatase OS=Lactobacillus plantarum (strain ATCC BAA-793 / NCIMB 8826 / WCFS1) OX=220668 GN=lp_2235 PE=3 SV=1	1.588887155	1.18E-05	up
F9ULU2	Ribosomal RNA small subunit methyltransferase I	Ribosomal RNA small subunit methyltransferase I OS=Lactobacillus plantarum (strain ATCC BAA-793 / NCIMB 8826 / WCFS1) OX=220668 GN=rsmI PE=3 SV=1	1.721647793	8.63E-05	up
F9US22	tRNA-dihydrouridine synthase	tRNA-dihydrouridine synthase OS=Lactobacillus plantarum (strain ATCC BAA-793 / NCIMB 8826 / WCFS1) OX=220668 GN=dus3 PE=3 SV=1	1.875759153	6.83E-06	up
F9UT60	treA	Trehalose-6-phosphate hydrolase OS=Lactobacillus plantarum (strain ATCC BAA-793 / NCIMB 8826 / WCFS1) OX=220668 GN=treA PE=3 SV=1	2.220493875	4.34E-06	up
F9URP7	pbg5	6-phospho-beta-glucosidase OS=Lactobacillus plantarum (strain ATCC BAA-793 / NCIMB 8826 / WCFS1) OX=220668 GN=pbg5 PE=3 SV=1	1.75912044	1.38E-05	up
F9USS1	lp_0092	Oligopeptide ABC transporter,lipoprotein-binding protein OS=Lactobacillus plantarum (strain ATCC BAA-793 / NCIMB 8826 / WCFS1) OX=220668 GN=lp_0092 PE=4 SV=1	0.611781889	0.01674	down
F9UMB1	DNA helicase	DNA helicase OS=Lactobacillus plantarum (strain ATCC BAA-793 / NCIMB 8826 / WCFS1) OX=220668 GN=lp_0910 PE=1 SV=1	1.561966806	9.03E-07	up
F9UNR2	lp_1527	Hydrolase, HAD superfamily OS=Lactobacillus plantarum (strain ATCC BAA-793 / NCIMB 8826 / WCFS1) OX=220668 GN=lp_1527 PE=4 SV=1	1.904343239	9.44E-06	up
F9UR81	DUF1801 domain-containing protein	DUF1801 domain-containing protein OS=Lactobacillus plantarum (strain ATCC BAA-793 / NCIMB 8826 / WCFS1) OX=220668 GN=lp_2566 PE=4 SV=1	1.592016344	0.001995	up
F9USZ3	mapB	Maltose phosphorylase OS=Lactobacillus plantarum (strain ATCC BAA-793 / NCIMB 8826 / WCFS1) OX=220668 GN=mapB PE=3 SV=1	1.660480878	1.51E-05	up
F9UP44	Acetyl-CoA carboxytransferase	Acetyl-CoA carboxytransferase OS=Lactobacillus plantarum (strain ATCC BAA-793 / NCIMB 8826 / WCFS1) OX=220668 GN=accA2 PE=4 SV=1	1.639659875	1.85E-05	up

Q88XY8	Elongation factor G	Elongation factor G OS=Lactobacillus plantarum (strain ATCC BAA-793 / NCIMB 8826 / WCFS1) OX=220668 GN=fusA PE=3 SV=1	1.500684203	6.88E-07	up
F9ULT4	DNA polymerase III subunit gamma/tau	DNA polymerase III subunit gamma/tau OS=Lactobacillus plantarum (strain ATCC BAA-793 / NCIMB 8826 / WCFS1) OX=220668 GN=dnaX PE=3 SV=1	1.818181818	9.02E-07	up
Q88VM8	D-alanyl carrier protein 1	D-alanyl carrier protein 1 OS=Lactobacillus plantarum (strain ATCC BAA-793 / NCIMB 8826 / WCFS1) OX=220668 GN=dltC1 PE=3 SV=1	0.661234818	0.003393	down
F9UPN7	lp_1914	Transcription regulator, MarR family OS=Lactobacillus plantarum (strain ATCC BAA-793 / NCIMB 8826 / WCFS1) OX=220668 GN=lp_1914 PE=4 SV=1	2.06928839	0.000465	up
F9USI6	lp_3069	Medium-chain degydrogenase/reductase, Qor family OS=Lactobacillus plantarum (strain ATCC BAA-793 / NCIMB 8826 / WCFS1) OX=220668 GN=lp_3069 PE=4 SV=1	1.615015974	0.0001186	up
F9USF1	lp_3024	Transcription regulator, LysR family OS=Lactobacillus plantarum (strain ATCC BAA-793 / NCIMB 8826 / WCFS1) OX=220668 GN=lp_3024 PE=4 SV=1	1.567457201	3.84E-06	up
F9UQP6	lp_2342	Transcription regulator, Xre family OS=Lactobacillus plantarum (strain ATCC BAA-793 / NCIMB 8826 / WCFS1) OX=220668 GN=lp_2342 PE=4 SV=1	1.898172787	0.0001217	up
F9UQU1	lp_2401	Prophage P2a protein 56, lysin OS=Lactobacillus plantarum (strain ATCC BAA-793 / NCIMB 8826 / WCFS1) OX=220668 GN=lp_2401 PE=3 SV=1	0.5981409	0.02298	down
F9UP86	galM2	Aldose 1-epimerase OS=Lactobacillus plantarum (strain ATCC BAA-793 / NCIMB 8826 / WCFS1) OX=220668 GN=galM2 PE=4 SV=1	1.63812601	1.74E-05	up
Q88WG8	Beta-ketoacyl-[acyl-carrier-protein] synthase III 2	3-oxoacyl-[acyl-carrier-protein] synthase 3 protein 2 OS=Lactobacillus plantarum (strain ATCC BAA-793 / NCIMB 8826 / WCFS1) OX=220668 GN=fabH2 PE=3 SV=1	1.516736079	0.0001621	up
Q88U29	Bifunctional purine biosynthesis protein PurH	Bifunctional purine biosynthesis protein PurH OS=Lactobacillus plantarum (strain ATCC BAA-793 / NCIMB 8826 / WCFS1) OX=220668 GN=purH PE=3 SV=1	1.899590529	6.63E-06	up
F9ULD3	Catalase	Catalase OS=Lactobacillus plantarum (strain ATCC BAA-793 / NCIMB 8826 / WCFS1) OX=220668 GN=kat PE=3 SV=1	1.844405594	1.46E-06	up
F9UL62	Acetyl-CoA carboxytransferase	Acetyl-CoA carboxytransferase OS=Lactobacillus plantarum (strain ATCC BAA-793 / NCIMB 8826 / WCFS1) OX=220668 GN=accA1 PE=4 SV=1	1.700996678	0.000264	up
Q88VS1	DNA repair protein RecO	DNA repair protein RecO OS=Lactobacillus plantarum (strain ATCC BAA-793 / NCIMB 8826 / WCFS1) OX=220668 GN=recO PE=3 SV=1	1.744127091	5.96E-06	up
F9UM42	lp_0824	Hydrolase, HAD superfamily, Cof family OS=Lactobacillus plantarum (strain ATCC BAA-793 / NCIMB 8826 / WCFS1) OX=220668 GN=lp_0824 PE=4 SV=1	1.616959536	3.32E-05	up
F9ULL4	lp_3668	Acetyltransferase, GNAT family OS=Lactobacillus plantarum (strain ATCC BAA-793 / NCIMB 8826 / WCFS1) OX=220668 GN=lp_3668 PE=4 SV=1	1.6968589	0.0006968	up
F9UL60	accC1	Biotin carboxylase OS=Lactobacillus plantarum (strain ATCC BAA-793 / NCIMB 8826 / WCFS1) OX=220668 GN=accC1 PE=4 SV=1	1.990757856	8.61E-07	up
F9UN47	SlpA domain-containing protein	Uncharacterized protein OS=Lactobacillus plantarum (strain ATCC BAA-793 / NCIMB 8826 / WCFS1) OX=220668 GN=lp_1257 PE=4 SV=1	1.928299837	6.13E-06	up
F9UPE8	lp_1801	Galactose-1-phosphate uridylyltransferase, HIT family OS=Lactobacillus plantarum (strain ATCC BAA-793 / NCIMB 8826 / WCFS1) OX=220668 GN=lp_1801 PE=4 SV=1	1.588499119	2.08E-06	up
F9UPE1	Rqc2 homolog RqcH	Rqc2 homolog RqcH OS=Lactobacillus plantarum (strain ATCC BAA-793 / NCIMB 8826 / WCFS1) OX=220668 GN=rqcH PE=3 SV=1	2.216464022	2.14E-06	up
Q88ZU5	Phosphoserine aminotransferase	Phosphoserine aminotransferase OS=Lactobacillus plantarum (strain ATCC BAA-793 / NCIMB 8826 / WCFS1) OX=220668 GN=serC PE=3 SV=1	1.533396197	0.005415	up

F9UM19	Ribonuclease R	Ribonuclease R OS=Lactobacillus plantarum (strain ATCC BAA-793 / NCIMB 8826 / WCFS1) OX=220668 GN=rnr PE=3 SV=1	1.640176962	1.10E-06	up
F9ULL3	lp_3666	Aromatic compound hydratase/decarboxylase OS=Lactobacillus plantarum (strain ATCC BAA-793 / NCIMB 8826 / WCFS1) OX=220668 GN=lp_3666 PE=4 SV=1	1.631260229	0.0007682	up
F9ULS9	Ribonucleoside-diphosphate reductase	Ribonucleoside-diphosphate reductase OS=Lactobacillus plantarum (strain ATCC BAA-793 / NCIMB 8826 / WCFS1) OX=220668 GN=nrdF PE=3 SV=1	1.74619463	2.50E-05	up
F9UQY0	lp_2441	Prophage P2a protein 16 OS=Lactobacillus plantarum (strain ATCC BAA-793 / NCIMB 8826 / WCFS1) OX=220668 GN=lp_2441 PE=4 SV=1	0.586264822	0.03203	down
F9UPS5	lp_1958	Acetoin ABC transporter, ATP-binding protein OS=Lactobacillus plantarum (strain ATCC BAA-793 / NCIMB 8826 / WCFS1) OX=220668 GN=lp_1958 PE=4 SV=1	1.698335295	3.87E-07	up
F9UT43	lp_0244	NADPH-dependent FMN reductase family protein OS=Lactobacillus plantarum (strain ATCC BAA-793 / NCIMB 8826 / WCFS1) OX=220668 GN=lp_0244 PE=4 SV=1	1.515298917	7.22E-05	up
Q88YL7	Protein translocase subunit SecA	Protein translocase subunit SecA OS=Lactobacillus plantarum (strain ATCC BAA-793 / NCIMB 8826 / WCFS1) OX=220668 GN=secA PE=3 SV=1	1.612391695	5.61E-06	up
F9UUJ2	bglG5	Transcription antiterminator, BlgB family OS=Lactobacillus plantarum (strain ATCC BAA-793 / NCIMB 8826 / WCFS1) OX=220668 GN=bglG5 PE=4 SV=1	1.511006289	1.54E-05	up
F9UPQ0	DUF1722 domain-containing protein	DUF1722 domain-containing protein OS=Lactobacillus plantarum (strain ATCC BAA-793 / NCIMB 8826 / WCFS1) OX=220668 GN=lp_1929 PE=4 SV=1	2.696316887	1.60E-05	up
F9UQ72	holA	DNA-directed DNA polymerase III, delta chain OS=Lactobacillus plantarum (strain ATCC BAA-793 / NCIMB 8826 / WCFS1) OX=220668 GN=holA PE=4 SV=1	1.777816472	1.28E-05	up
Q88U30	Phosphoribosylamine--glycine ligase	Phosphoribosylamine--glycine ligase OS=Lactobacillus plantarum (strain ATCC BAA-793 / NCIMB 8826 / WCFS1) OX=220668 GN=purD PE=3 SV=1	1.647000166	0.002954	up
F9UN56	lp_1267	Transcription regulator, MarR family OS=Lactobacillus plantarum (strain ATCC BAA-793 / NCIMB 8826 / WCFS1) OX=220668 GN=lp_1267 PE=4 SV=1	4.532499131	4.21E-06	up
Q88VM6	D-alanine--D-alanyl carrier protein ligase	D-alanine--D-alanyl carrier protein ligase OS=Lactobacillus plantarum (strain ATCC BAA-793 / NCIMB 8826 / WCFS1) OX=220668 GN=dltA PE=3 SV=1	1.806268815	3.78E-06	up
F9ULU0	holB	DNA-directed DNA polymerase III, delta' subunit OS=Lactobacillus plantarum (strain ATCC BAA-793 / NCIMB 8826 / WCFS1) OX=220668 GN=holB PE=4 SV=1	2.210953347	3.89E-06	up
F9ULW3	YigZ family protein	Uncharacterized protein OS=Lactobacillus plantarum (strain ATCC BAA-793 / NCIMB 8826 / WCFS1) OX=220668 GN=lp_0734 PE=3 SV=1	1.65993266	3.59E-05	up
F9UQL0	lp_2299	Metallophosphatase (MPP) superfamily protein OS=Lactobacillus plantarum (strain ATCC BAA-793 / NCIMB 8826 / WCFS1) OX=220668 GN=lp_2299 PE=4 SV=1	1.571803709	0.0004112	up
F9US70	lp_0048	Uracil-DNA glycosylase family protein OS=Lactobacillus plantarum (strain ATCC BAA-793 / NCIMB 8826 / WCFS1) OX=220668 GN=lp_0048 PE=4 SV=1	1.56950599	3.25E-06	up
F9URG1	nrdI	Ribonucleotide reductase protein NrdI OS=Lactobacillus plantarum (strain ATCC BAA-793 / NCIMB 8826 / WCFS1) OX=220668 GN=nrdI PE=4 SV=1	1.575037147	0.001588	up
Q88YI7	UvrABC system protein A	UvrABC system protein A OS=Lactobacillus plantarum (strain ATCC BAA-793 / NCIMB 8826 / WCFS1) OX=220668 GN=uvrA PE=3 SV=1	1.692214742	9.03E-05	up
F9UPU8	lp_1985	Phosphohydrolase OS=Lactobacillus plantarum (strain ATCC BAA-793 / NCIMB 8826 / WCFS1) OX=220668 GN=lp_1985 PE=4 SV=1	1.683304647	2.24E-05	up
Q88WD0	Probable tautomerase lp_1712	Probable tautomerase lp_1712 OS=Lactobacillus plantarum (strain ATCC BAA-793 / NCIMB 8826 / WCFS1) OX=220668 GN=lp_1712 PE=3 SV=3	1.717653222	0.005115	up
F9UNS7	rrp5	Two-component system response regulator OS=Lactobacillus plantarum (strain ATCC BAA-793 / NCIMB 8826 / WCFS1) OX=220668 GN=rrp5 PE=4 SV=1	1.534951931	6.03E-05	up
F9UPH6	DNA topoisom	DNA topoisomerase 4 subunit B OS=Lactobacillus plantarum (strain ATCC BAA-793 / NCIMB 8826 / WCFS1) OX=220668 GN=parE PE=3	1.882069349	1.58E-06	up

	erase 4 subunit B	SV=1				
F9UNK2	DNA translocase FtsK	DNA translocase FtsK OS=Lactobacillus plantarum (strain ATCC BAA-793 / NCIMB 8826 / WCFS1) OX=220668 GN=ftsK1 PE=3 SV=1	1.514026269	1.45E-05	up
F9USH5	copA	Copper transporting ATPase OS=Lactobacillus plantarum (strain ATCC BAA-793 / NCIMB 8826 / WCFS1) OX=220668 GN=copA PE=3 SV=1	0.451500938	0.001123	down
F9URU3	lp_2840	Ribosomal protein serine-acetylating enzyme OS=Lactobacillus plantarum (strain ATCC BAA-793 / NCIMB 8826 / WCFS1) OX=220668 GN=lp_2840 PE=4 SV=1	1.987241446	0.0005433	up
F9URR7	lp_2802	2,5 diketo-D-gluconic acid-like reductase NADP dependent (Promiscuous) OS=Lactobacillus plantarum (strain ATCC BAA-793 / NCIMB 8826 / WCFS1) OX=220668 GN=lp_2802 PE=4 SV=1	1.57841991	0.0002703	up
F9ULT0	Ribonucleoside-diphosphate reductase	Ribonucleoside-diphosphate reductase OS=Lactobacillus plantarum (strain ATCC BAA-793 / NCIMB 8826 / WCFS1) OX=220668 GN=nrdE PE=3 SV=1	2.396730727	2.49E-08	up
Q88V96	tRNA-specific 2-thiouridylase MnmA	tRNA-specific 2-thiouridylase MnmA OS=Lactobacillus plantarum (strain ATCC BAA-793 / NCIMB 8826 / WCFS1) OX=220668 GN=mnmA PE=3 SV=1	1.683353755	7.38E-06	up
F9UP26	lp_1660	Alcohol dehydrogenase, zinc-binding OS=Lactobacillus plantarum (strain ATCC BAA-793 / NCIMB 8826 / WCFS1) OX=220668 GN=lp_1660 PE=4 SV=1	1.569914185	8.18E-06	up
F9UL77	trmH	tRNA/rRNA methyltransferase, TrmH family OS=Lactobacillus plantarum (strain ATCC BAA-793 / NCIMB 8826 / WCFS1) OX=220668 GN=trmH PE=4 SV=1	1.774830803	2.61E-05	up
F9URV3	lp_2851	Short-chain dehydrogenase/oxidoreductase OS=Lactobacillus plantarum (strain ATCC BAA-793 / NCIMB 8826 / WCFS1) OX=220668 GN=lp_2851 PE=3 SV=1	1.535385645	0.0002515	up
F9UT56	pnuC1	Nicotinamide mononucleotide transporter OS=Lactobacillus plantarum (strain ATCC BAA-793 / NCIMB 8826 / WCFS1) OX=220668 GN=pnuC1 PE=3 SV=1	2.375	6.37E-06	up
F9UM45	Phage protein	Uncharacterized protein OS=Lactobacillus plantarum (strain ATCC BAA-793 / NCIMB 8826 / WCFS1) OX=220668 GN=lp_0827 PE=4 SV=1	1.51031614	0.0009739	up
Q88Z54	UDP-N-acetylglucosamine 1-carboxyvinyltransferase 2	UDP-N-acetylglucosamine 1-carboxyvinyltransferase 2 OS=Lactobacillus plantarum (strain ATCC BAA-793 / NCIMB 8826 / WCFS1) OX=220668 GN=murA2 PE=3 SV=1	1.836580291	4.93E-07	up
F9URG4	lp_2676	Transcription regulator, MerR family OS=Lactobacillus plantarum (strain ATCC BAA-793 / NCIMB 8826 / WCFS1) OX=220668 GN=lp_2676 PE=4 SV=1	1.635570589	0.0007663	up
Q88VL5	tRNA pseudouridine synthase B	tRNA pseudouridine synthase B OS=Lactobacillus plantarum (strain ATCC BAA-793 / NCIMB 8826 / WCFS1) OX=220668 GN=truB PE=3 SV=1	1.852424184	1.05E-07	up
F9UQU0	lp_2400	Prophage P2a protein 57 OS=Lactobacillus plantarum (strain ATCC BAA-793 / NCIMB 8826 / WCFS1) OX=220668 GN=lp_2400 PE=4 SV=1	0.657995495	0.0104	down
F9ULI4	lp_3635	ABC transporter, ATP-binding protein, mannose related oligosaccharides OS=Lactobacillus plantarum (strain ATCC BAA-793 / NCIMB 8826 / WCFS1) OX=220668 GN=lp_3635 PE=4 SV=1	1.792689891	2.04E-05	up
F9UPI8	lp_1856	5-methyltetrahydropteroyltriglutamate-homocysteine methyltransferase (Cobalamine-independent methonine synthase) OS=Lactobacillus plantarum (strain ATCC BAA-793 / NCIMB 8826 / WCFS1) OX=220668 GN=lp_1856 PE=4 SV=1	2.404205607	2.36E-06	up
Q88WZ9	tRNA (guanine-N(7)-)-methyltransferase	tRNA (guanine-N(7)-)-methyltransferase OS=Lactobacillus plantarum (strain ATCC BAA-793 / NCIMB 8826 / WCFS1) OX=220668 GN=trmB PE=3 SV=1	1.803774312	2.59E-05	up
Q88WK6	50S ribosomal protein L28	50S ribosomal protein L28 OS=Lactobacillus plantarum (strain ATCC BAA-793 / NCIMB 8826 / WCFS1) OX=220668 GN=rpmB PE=3 SV=1	0.381766382	5.51E-05	down
F9UTF7	crtM	Dehydrosqualene synthase OS=Lactobacillus plantarum (strain ATCC BAA-793 / NCIMB 8826 / WCFS1) OX=220668 GN=crtM PE=4 SV=1	2.398783056	3.95E-05	up

F9UM76	Transcriptional regulator	Uncharacterized protein OS=Lactobacillus plantarum (strain ATCC BAA-793 / NCIMB 8826 / WCFS1) OX=220668 GN=lp_0865 PE=4 SV=1	0.410893032	3.59E-05	down
F9UN61	lp_1275	1,2-diacylglycerol 3-glucosyltransferase OS=Lactobacillus plantarum (strain ATCC BAA-793 / NCIMB 8826 / WCFS1) OX=220668 GN=lp_1275 PE=4 SV=1	1.960230785	1.18E-05	up
F9UPP9	spx2	RNA polymerase (RNAP)-binding regulatory protein, arsenate reductase (ArsC) family, Spx subfamily OS=Lactobacillus plantarum (strain ATCC BAA-793 / NCIMB 8826 / WCFS1) OX=220668 GN=spx2 PE=3 SV=1	0.264472498	3.53E-05	down
F9UU28	tRNA-dihydrouridine synthase	tRNA-dihydrouridine synthase OS=Lactobacillus plantarum (strain ATCC BAA-793 / NCIMB 8826 / WCFS1) OX=220668 GN=dus1 PE=3 SV=1	1.586162362	0.0005255	up
Q88VI7	UPF0291 protein lp_2062	UPF0291 protein lp_2062 OS=Lactobacillus plantarum (strain ATCC BAA-793 / NCIMB 8826 / WCFS1) OX=220668 GN=lp_2062 PE=3 SV=1	1.646489564	0.0001104	up
Q88VP9	Ribosomal protein L11 methyltransferase	Ribosomal protein L11 methyltransferase OS=Lactobacillus plantarum (strain ATCC BAA-793 / NCIMB 8826 / WCFS1) OX=220668 GN=prmA PE=3 SV=1	1.556985294	0.0001127	up
Q6LWD7	DUF3847 domain-containing protein	Uncharacterized protein OS=Lactobacillus plantarum (strain ATCC BAA-793 / NCIMB 8826 / WCFS1) OX=220668 GN=orf40 PE=4 SV=1	1.824091001	0.0001402	up
F9UND2	gtcA1	Teichoic acid glycosylation protein OS=Lactobacillus plantarum (strain ATCC BAA-793 / NCIMB 8826 / WCFS1) OX=220668 GN=gtcA1 PE=3 SV=1	1.56987061	4.14E-05	up
F9UM47	lp_0829	Nitroreductase family protein OS=Lactobacillus plantarum (strain ATCC BAA-793 / NCIMB 8826 / WCFS1) OX=220668 GN=lp_0829 PE=3 SV=1	2.395107487	1.54E-05	up
F9UPU1	tagB3	Glycosyl/glycerophosphate transferase, teichoic acid biosynthesis protein B OS=Lactobacillus plantarum (strain ATCC BAA-793 / NCIMB 8826 / WCFS1) OX=220668 GN=tagB3 PE=3 SV=1	1.685229541	0.001292	up
F9UM31	exoA	Exodeoxyribonuclease III OS=Lactobacillus plantarum (strain ATCC BAA-793 / NCIMB 8826 / WCFS1) OX=220668 GN=exoA PE=3 SV=1	1.562170603	3.48E-05	up
Q88WM6	Exodeoxyribonuclease 7 small subunit	Exodeoxyribonuclease 7 small subunit OS=Lactobacillus plantarum (strain ATCC BAA-793 / NCIMB 8826 / WCFS1) OX=220668 GN=xseB PE=3 SV=1	1.598849891	0.008463	up
F9USD9	lp_3012	NAD-dependent epimerase/dehydratase protein family OS=Lactobacillus plantarum (strain ATCC BAA-793 / NCIMB 8826 / WCFS1) OX=220668 GN=lp_3012 PE=4 SV=1	1.557226563	0.00055	up
F9UTT4	lp_0332	Hypothetical membrane protein OS=Lactobacillus plantarum (strain ATCC BAA-793 / NCIMB 8826 / WCFS1) OX=220668 GN=lp_0332 PE=4 SV=1	1.876203899	0.001628	up
F9UM81	gph1	Phosphohydrolase OS=Lactobacillus plantarum (strain ATCC BAA-793 / NCIMB 8826 / WCFS1) OX=220668 GN=gph1 PE=1 SV=1	1.776923077	0.001575	up
F9UUF2	rafP	Putative PTS system EIIA component OS=Lactobacillus plantarum (strain ATCC BAA-793 / NCIMB 8826 / WCFS1) OX=220668 GN=rafP PE=3 SV=1	0.639510635	0.003308	down
F9UPR0	lp_1939	Oxidoreductase, medium chain dehydrogenases/reductase (MDR)/zinc-dependent alcohol dehydrogenase-like family OS=Lactobacillus plantarum (strain ATCC BAA-793 / NCIMB 8826 / WCFS1) OX=220668 GN=lp_1939 PE=4 SV=1	2.368221574	2.21E-05	up
F9UQ59	Glycogen biosynthesis protein GlgD	Uncharacterized protein OS=Lactobacillus plantarum (strain ATCC BAA-793 / NCIMB 8826 / WCFS1) OX=220668 GN=lp_2112 PE=4 SV=1	0.578895602	0.009178	down
F9UT44	lp_0245	Transcription regulator, GntR family OS=Lactobacillus plantarum (strain ATCC BAA-793 / NCIMB 8826 / WCFS1) OX=220668 GN=lp_0245 PE=4 SV=1	1.673663656	4.40E-05	up
F9UTB4	lp_3207	PLP-dependent aminotransferase OS=Lactobacillus plantarum (strain ATCC BAA-793 / NCIMB 8826 / WCFS1) OX=220668 GN=lp_3207 PE=4 SV=1	1.795814328	2.58E-05	up
F9UTT5	NfeD domain-containing protein	Uncharacterized protein OS=Lactobacillus plantarum (strain ATCC BAA-793 / NCIMB 8826 / WCFS1) OX=220668 GN=lp_0333 PE=4 SV=1	0.239185412	8.81E-05	down

	F9ULR3	lp_0675	Prophage P1 protein 52, endolysin OS=Lactobacillus plantarum (strain ATCC BAA-793 / NCIMB 8826 / WCFS1) OX=220668 GN=lp_0675 PE=3 SV=1	0.4608 8896	0.02079	down
	F9UMT0	DUF2187 domain-c ontaining protein	Uncharacterized protein OS=Lactobacillus plantarum (strain ATCC BAA-793 / NCIMB 8826 / WCFS1) OX=220668 GN=lp_1123 PE=4 SV=1	0.6122 58065	0.01126	down
	F9UUC4	lp_3451	Cell surface protein, CscA/DUF916 family OS=Lactobacillus plantarum (strain ATCC BAA-793 / NCIMB 8826 / WCFS1) OX=220668 GN=lp_3451 PE=4 SV=1	0.6652 92263	0.00683 1	down
**P/C**	F9UMQ5	ttdB	L(+)-tartrate dehydratase, subunit B OS=Lactobacillus plantarum (strain ATCC BAA-793 / NCIMB 8826 / WCFS1) OX=220668 GN=ttdB PE=3 SV=1	0.0939 30348	6.27E-0 5	down
	F9USC4	S9 family peptidase	Uncharacterized protein OS=Lactobacillus plantarum (strain ATCC BAA-793 / NCIMB 8826 / WCFS1) OX=220668 GN=lp_2994 PE=4 SV=1	0.0989 32269	2.68E-0 6	down
	F9UP14	lp_1643	Mucus-binding protein, LPXTG-motif cell wall anchor OS=Lactobacillus plantarum (strain ATCC BAA-793 / NCIMB 8826 / WCFS1) OX=220668 GN=lp_1643 PE=4 SV=1	0.1241 03109	1.65E-0 7	down
	F9USB9	zmp3	Extracellular zinc metalloproteinase, M10 family OS=Lactobacillus plantarum (strain ATCC BAA-793 / NCIMB 8826 / WCFS1) OX=220668 GN=zmp3 PE=4 SV=1	0.1424 41054	1.54E-0 5	down
	Q6LWH7	repA	Copy-number control protein OS=Lactobacillus plantarum (strain ATCC BAA-793 / NCIMB 8826 / WCFS1) OX=220668 GN=repA PE=4 SV=1	0.1491 0282	1.36E-0 8	down
	F9URU9	lp_2847	Extracellular transglycosylase, with LysM peptidoglycan binding domain OS=Lactobacillus plantarum (strain ATCC BAA-793 / NCIMB 8826 / WCFS1) OX=220668 GN=lp_2847 PE=4 SV=1	0.1550 90531	0.00024 64	down
	F9UTQ7	lp_0302	Extracellular transglycosylase OS=Lactobacillus plantarum (strain ATCC BAA-793 / NCIMB 8826 / WCFS1) OX=220668 GN=lp_0302 PE=4 SV=1	0.1535 37818	0.00010 42	down
	F9UQA0	lp_2162	Extracellular protein, NlpC/P60 family,gamma-D-glutamate-meso-diaminopimelate muropeptidase OS=Lactobacillus plantarum (strain ATCC BAA-793 / NCIMB 8826 / WCFS1) OX=220668 GN=lp_2162 PE=3 SV=1	0.1810 34483	0.00019 62	down
	F9USE1	lp_3014	Extracellular transglycosylase, with LysM peptidoglycan binding domain OS=Lactobacillus plantarum (strain ATCC BAA-793 / NCIMB 8826 / WCFS1) OX=220668 GN=lp_3014 PE=4 SV=1	0.1989 93851	0.00020 63	down
	F9UU93	lp_3414	Cell surface protein, CscB family OS=Lactobacillus plantarum (strain ATCC BAA-793 / NCIMB 8826 / WCFS1) OX=220668 GN=lp_3414 PE=4 SV=1	0.1884 60097	0.00038 9	down
	F9UP60	lp_1697	Adherence protein, chitin-binding domain OS=Lactobacillus plantarum (strain ATCC BAA-793 / NCIMB 8826 / WCFS1) OX=220668 GN=lp_1697 PE=4 SV=1	0.2107 75607	0.00012 07	down
	F9UM79	lp_0869	Extracellular protein, Ser/Thr-rich OS=Lactobacillus plantarum (strain ATCC BAA-793 / NCIMB 8826 / WCFS1) OX=220668 GN=lp_0869 PE=4 SV=1	0.1838 94612	6.26E-0 5	down
	F9UUA0	lp_3421	Extracellular protein,gamma-D-glutamate-meso-diaminopimelate muropeptidase OS=Lactobacillus plantarum (strain ATCC BAA-793 / NCIMB 8826 / WCFS1) OX=220668 GN=lp_3421 PE=3 SV=1	0.2331 31689	5.54E-0 5	down
	P77889	Orotate phosphori bosyltrans ferase	Orotate phosphoribosyltransferase OS=Lactobacillus plantarum (strain ATCC BAA-793 / NCIMB 8826 / WCFS1) OX=220668 GN=pyrE PE=3 SV=1	0.2237 31838	2.29E-0 5	down
	F9US24	lp_2940	Cell surface protein, LPXTG-motif cell wall anchor OS=Lactobacillus plantarum (strain ATCC BAA-793 / NCIMB 8826 / WCFS1) OX=220668 GN=lp_2940 PE=4 SV=1	0.2476 95128	0.00014 82	down
	F9USJ7	lp_3081	Transcription regulator, MarR family OS=Lactobacillus plantarum (strain ATCC BAA-793 / NCIMB 8826 / WCFS1) OX=220668 GN=lp_3081 PE=4 SV=1	0.3139 23321	3.60E-0 5	down
	F9URU8	lp_2845	Extracellular transglycosylase, with LysM peptidoglycan binding domain OS=Lactobacillus plantarum (strain ATCC BAA-793 / NCIMB 8826 / WCFS1) OX=220668 GN=lp_2845 PE=4 SV=1	0.3042 10828	0.00024 89	down
	Q890I8	Glycogen synthase	Glycogen synthase OS=Lactobacillus plantarum (strain ATCC BAA-793 / NCIMB 8826 / WCFS1) OX=220668 GN=glgA PE=3 SV=1	0.2870 58153	0.00013 97	down
	F9UM68	lp_0856	Acyltransferase OS=Lactobacillus plantarum (strain ATCC BAA-793 / NCIMB 8826 / WCFS1) OX=220668 GN=lp_0856 PE=4 SV=1	0.2675 80554	0.00010 78	down
	F9URS2	lp_2809	Extracellular protein OS=Lactobacillus plantarum (strain ATCC BAA-793 / NCIMB 8826 / WCFS1) OX=220668 GN=lp_2809 PE=4 SV=1	0.3271 9715	0.00036 56	down
	F9URE0	pts19A	PTS system,N-acetylglucosamine/galactosamine-specific EIIA component OS=Lactobacillus plantarum (strain ATCC BAA-793 / NCIMB 8826 / WCFS1) OX=220668 GN=pts19A PE=4 SV=1	0.2808 17972	0.00050 52	down

F9URS4	lp_2812	Extracellular protein, membrane-anchored OS=Lactobacillus plantarum (strain ATCC BAA-793 / NCIMB 8826 / WCFS1) OX=220668 GN=lp_2812 PE=4 SV=1	0.277002356	0.0004463	down
F9UQZ9	lp_2463	Prophage P2b protein 18, major capsid protein OS=Lactobacillus plantarum (strain ATCC BAA-793 / NCIMB 8826 / WCFS1) OX=220668 GN=lp_2463 PE=4 SV=1	0.55463097	0.01655	down
F9USB8	Conserved domain protein	Uncharacterized protein OS=Lactobacillus plantarum (strain ATCC BAA-793 / NCIMB 8826 / WCFS1) OX=220668 GN=lp_2987 PE=4 SV=1	0.296840491	1.20E-05	down
F9UR89	lp_2575	Transporter, MMPL family OS=Lactobacillus plantarum (strain ATCC BAA-793 / NCIMB 8826 / WCFS1) OX=220668 GN=lp_2575 PE=4 SV=1	0.622634222	0.04378	down
F9USV1	hsp1	Small heat shock protein OS=Lactobacillus plantarum (strain ATCC BAA-793 / NCIMB 8826 / WCFS1) OX=220668 GN=hsp1 PE=3 SV=1	0.313767651	4.32E-05	down
F9UMC3	lp_0924	Extracellular protein, MORN repeat family OS=Lactobacillus plantarum (strain ATCC BAA-793 / NCIMB 8826 / WCFS1) OX=220668 GN=lp_0924 PE=4 SV=1	0.305251361	0.0001807	down
F9UU85	Inner membrane protein	Uncharacterized protein OS=Lactobacillus plantarum (strain ATCC BAA-793 / NCIMB 8826 / WCFS1) OX=220668 GN=lp_3406 PE=4 SV=1	0.368671424	0.0002843	down
P77885	Carbamoyl-phosphate synthase pyrimidine-specific small chain	Carbamoyl-phosphate synthase pyrimidine-specific small chain OS=Lactobacillus plantarum (strain ATCC BAA-793 / NCIMB 8826 / WCFS1) OX=220668 GN=pyrAA PE=3 SV=2	0.371974088	8.04E-06	down
F9UKV7	lp_0473	Lipoprotein OS=Lactobacillus plantarum (strain ATCC BAA-793 / NCIMB 8826 / WCFS1) OX=220668 GN=lp_0473 PE=4 SV=1	0.339039946	0.0002561	down
F9UME2	lp_0946	Mucus-binding protein, LPXTG-motif cell wall anchor OS=Lactobacillus plantarum (strain ATCC BAA-793 / NCIMB 8826 / WCFS1) OX=220668 GN=lp_0946 PE=4 SV=1	0.336810229	0.001403	down
P77887	Dihydroorotate dehydrogenase A (fumarate)	Dihydroorotate dehydrogenase A (fumarate) OS=Lactobacillus plantarum (strain ATCC BAA-793 / NCIMB 8826 / WCFS1) OX=220668 GN=pyrD PE=3 SV=2	0.319234609	1.90E-05	down
F9UU91	lp_3412	Cell surface protein, CscB family OS=Lactobacillus plantarum (strain ATCC BAA-793 / NCIMB 8826 / WCFS1) OX=220668 GN=lp_3412 PE=4 SV=1	0.313853821	6.03E-05	down
Q88VY3	UPF0756 membrane protein lp_1894	UPF0756 membrane protein lp_1894 OS=Lactobacillus plantarum (strain ATCC BAA-793 / NCIMB 8826 / WCFS1) OX=220668 GN=lp_1894 PE=3 SV=1	0.343868087	0.0001408	down
F9UT05	lp_0197	Cell surface protein, LPXTG-motif cell wall anchor OS=Lactobacillus plantarum (strain ATCC BAA-793 / NCIMB 8826 / WCFS1) OX=220668 GN=lp_0197 PE=4 SV=1	0.359554473	0.0001269	down
F9UTM5	hsp3	Small heat shock protein OS=Lactobacillus plantarum (strain ATCC BAA-793 / NCIMB 8826 / WCFS1) OX=220668 GN=hsp3 PE=3 SV=1	0.361821366	1.35E-05	down
F9US95	lp_2960	Lipase/esterase, subfamily of SGNH-hydrolases OS=Lactobacillus plantarum (strain ATCC BAA-793 / NCIMB 8826 / WCFS1) OX=220668 GN=lp_2960 PE=4 SV=1	0.358517463	5.78E-05	down
F9US93	wapA	Cell surface protein, LPXTG-motif cell wall anchor OS=Lactobacillus plantarum (strain ATCC BAA-793 / NCIMB 8826 / WCFS1) OX=220668 GN=wapA PE=4 SV=1	0.36771625	0.0001026	down
F9UNG3	30S ribosomal protein S21	Uncharacterized protein OS=Lactobacillus plantarum (strain ATCC BAA-793 / NCIMB 8826 / WCFS1) OX=220668 GN=lp_1412 PE=4 SV=1	0.405891354	0.001006	down
F9URZ8	lp_2909	Acetyltransferase, GNAT family OS=Lactobacillus plantarum (strain ATCC BAA-793 / NCIMB 8826 / WCFS1) OX=220668 GN=lp_2909 PE=4 SV=1	0.511560694	0.02185	down
F9USJ2	lp_3075	Cell surface protein, CscC family OS=Lactobacillus plantarum (strain ATCC BAA-793 / NCIMB 8826 / WCFS1) OX=220668 GN=lp_3075 PE=4 SV=1	0.375735849	0.01873	down
F9UMC2	lp_0923	Cell surface protein, LPXTG-motif cell wall anchor OS=Lactobacillus plantarum (strain ATCC BAA-793 / NCIMB 8826 / WCFS1) OX=220668 GN=lp_0923 PE=4 SV=1	0.406044169	0.001396	down
P77883	Aspartate carbamoy	Aspartate carbamoyltransferase OS=Lactobacillus plantarum (strain ATCC BAA-793 / NCIMB 8826 / WCFS1) OX=220668 GN=pyrB PE=3	0.386153846	2.29E-05	down

	ltransfera se	SV=1			
F9USW1	lp_0141	Extracellular protein OS=Lactobacillus plantarum (strain ATCC BAA-793 / NCIMB 8826 / WCFS1) OX=220668 GN=lp_0141 PE=4 SV=1	0.3470 58824	0.00014 81	down
F9URD4	lp_2636	Extracellular protein OS=Lactobacillus plantarum (strain ATCC BAA-793 / NCIMB 8826 / WCFS1) OX=220668 GN=lp_2636 PE=4 SV=1	0.4177 86561	0.00064 81	down
F9UNI8	lp_1446	Cell surface protein, CscB family OS=Lactobacillus plantarum (strain ATCC BAA-793 / NCIMB 8826 / WCFS1) OX=220668 GN=lp_1446 PE=4 SV=1	0.5817 05151	0.04567	down
F9UQX6	lp_2436	Prophage P2a protein 21 OS=Lactobacillus plantarum (strain ATCC BAA-793 / NCIMB 8826 / WCFS1) OX=220668 GN=lp_2436 PE=4 SV=1	0.5106 74637	0.00047 8	down
F9UUC3	lp_3450	Cell surface protein, CscC family OS=Lactobacillus plantarum (strain ATCC BAA-793 / NCIMB 8826 / WCFS1) OX=220668 GN=lp_3450 PE=4 SV=1	0.4199 18699	0.00042 4	down
F9UU40	Ribonucle ase M5	Ribonuclease M5 OS=Lactobacillus plantarum (strain ATCC BAA-793 / NCIMB 8826 / WCFS1) OX=220668 GN=rnmV PE=3 SV=1	0.5376 77305	0.00162 6	down
F9UMD1	lp_0932	Extracellular lipoprotein OS=Lactobacillus plantarum (strain ATCC BAA-793 / NCIMB 8826 / WCFS1) OX=220668 GN=lp_0932 PE=4 SV=1	0.4871 13402	0.00346	down
Q88YM6	Co-chaper onin GroES	Co-chaperonin GroES OS=Lactobacillus plantarum (strain ATCC BAA-793 / NCIMB 8826 / WCFS1) OX=220668 GN=groES PE=3 SV=1	0.4673 25881	0.00022 96	down
F9ULL9	lp_3676	Cell surface protein, CscC family OS=Lactobacillus plantarum (strain ATCC BAA-793 / NCIMB 8826 / WCFS1) OX=220668 GN=lp_3676 PE=4 SV=1	0.4673 86609	0.00062 19	down
F9UQT3	Glyco_hy dro_38C domain-c ontaining protein	Uncharacterized protein OS=Lactobacillus plantarum (strain ATCC BAA-793 / NCIMB 8826 / WCFS1) OX=220668 GN=lp_2391 PE=4 SV=1	0.5940 17094	0.00459 8	down
F9UPB8	lp_1767	Glycosyl hydrolase, family 25 OS=Lactobacillus plantarum (strain ATCC BAA-793 / NCIMB 8826 / WCFS1) OX=220668 GN=lp_1767 PE=3 SV=1	0.4582 78724	0.00022 38	down
F9USW8	lp_0154	Transcription regulator, PadR family OS=Lactobacillus plantarum (strain ATCC BAA-793 / NCIMB 8826 / WCFS1) OX=220668 GN=lp_0154 PE=4 SV=1	0.5891 91774	0.00248 3	down
F9UM52	spx1	RNA polymerase (RNAP)-binding regulatory protein, arsenate reductase (ArsC) family, Spx subfamily OS=Lactobacillus plantarum (strain ATCC BAA-793 / NCIMB 8826 / WCFS1) OX=220668 GN=spx1 PE=3 SV=1	0.3749 27476	1.23E-0 5	down
F9UNC6	Pseudouri dylate synthase	Uncharacterized protein OS=Lactobacillus plantarum (strain ATCC BAA-793 / NCIMB 8826 / WCFS1) OX=220668 GN=lp_1362 PE=4 SV=1	0.3798 16896	1.26E-0 5	down
F9UMH5	lp_0988	Extracellular lipoprotein, Asp-rich OS=Lactobacillus plantarum (strain ATCC BAA-793 / NCIMB 8826 / WCFS1) OX=220668 GN=lp_0988 PE=4 SV=1	0.4396 17724	0.00016 62	down
F9UMC4	lp_0925	Acyltransferase OS=Lactobacillus plantarum (strain ATCC BAA-793 / NCIMB 8826 / WCFS1) OX=220668 GN=lp_0925 PE=4 SV=1	0.3835 50147	0.00025 33	down
F9UTQ8	lp_0304	Extracellular transglycosylase OS=Lactobacillus plantarum (strain ATCC BAA-793 / NCIMB 8826 / WCFS1) OX=220668 GN=lp_0304 PE=4 SV=1	0.4281 38718	4.98E-0 5	down
F9UNC2	lp_1357	Extracellular protein, membrane-anchored OS=Lactobacillus plantarum (strain ATCC BAA-793 / NCIMB 8826 / WCFS1) OX=220668 GN=lp_1357 PE=4 SV=1	0.4332 45033	0.00052 86	down
F9ULN5	lp_0646	Prophage P1 protein 23 OS=Lactobacillus plantarum (strain ATCC BAA-793 / NCIMB 8826 / WCFS1) OX=220668 GN=lp_0646 PE=4 SV=1	0.5519 73051	0.03885	down
F9USM7	lp_3114	Mucus-binding protein, LPXTG-motif cell wall anchor OS=Lactobacillus plantarum (strain ATCC BAA-793 / NCIMB 8826 / WCFS1) OX=220668 GN=lp_3114 PE=4 SV=1	0.4669 11765	0.00069 1	down
F9URZ3	lp_2901	Hypothetical membrane protein OS=Lactobacillus plantarum (strain ATCC BAA-793 / NCIMB 8826 / WCFS1) OX=220668 GN=lp_2901 PE=4 SV=1	0.4811 89039	0.00080 42	down
F9UMF6	lp_0961	Transposase OS=Lactobacillus plantarum (strain ATCC BAA-793 / NCIMB 8826 / WCFS1) OX=220668 GN=lp_0961 PE=4 SV=1	0.4932 95019	0.03491	down
F9USK7	lp_3093	Lysozyme/muramidase, glycoside hydrolase family 25 OS=Lactobacillus plantarum (strain ATCC BAA-793 / NCIMB 8826 / WCFS1) OX=220668 GN=lp_3093 PE=3 SV=1	0.4814 46242	0.00082 12	down
F9ULX3	Phosphat e-binding protein	Phosphate-binding protein OS=Lactobacillus plantarum (strain ATCC BAA-793 / NCIMB 8826 / WCFS1) OX=220668 GN=pstE PE=3 SV=1	0.4694 24286	2.41E-0 5	down
Q88X33	UPF0342 protein	UPF0342 protein lp_1415 OS=Lactobacillus plantarum (strain ATCC BAA-793 / NCIMB 8826 / WCFS1) OX=220668 GN=lp_1415 PE=3 SV=1	0.6096 91161	0.00185	down

	lp_1415				
F9URD9	acm2	Cell wall hydrolase/muramidase OS=Lactobacillus plantarum (strain ATCC BAA-793 / NCIMB 8826 / WCFS1) OX=220668 GN=acm2 PE=3 SV=1	0.4656 47744	0.00019 13	down
F9UPF5	lp_1812	Lipoprotein OS=Lactobacillus plantarum (strain ATCC BAA-793 / NCIMB 8826 / WCFS1) OX=220668 GN=lp_1812 PE=4 SV=1	0.4601 85638	0.00016 62	down
F9UTM7	lp_3355	Short-chain dehydrogenase/oxidoreductase,atypical SDR family, subgroup 6 OS=Lactobacillus plantarum (strain ATCC BAA-793 / NCIMB 8826 / WCFS1) OX=220668 GN=lp_3355 PE=4 SV=1	0.4777 22772	0.00011 87	down
F9USL7	fhuD	Iron chelatin ABC transporter, substrate binding protein OS=Lactobacillus plantarum (strain ATCC BAA-793 / NCIMB 8826 / WCFS1) OX=220668 GN=fhuD PE=4 SV=1	0.4689 96063	0.00059 48	down
F9URS3	lp_2810	Glycosyl hydrolase, family 25 OS=Lactobacillus plantarum (strain ATCC BAA-793 / NCIMB 8826 / WCFS1) OX=220668 GN=lp_2810 PE=3 SV=1	0.6297 91895	0.00471 3	down
F9UQH9	lp_2260	Extracellular protein, DUF336 family OS=Lactobacillus plantarum (strain ATCC BAA-793 / NCIMB 8826 / WCFS1) OX=220668 GN=lp_2260 PE=4 SV=1	0.5814 20182	0.00144 2	down
Q88S51	L-rhamnose isomerase	L-rhamnose isomerase OS=Lactobacillus plantarum (strain ATCC BAA-793 / NCIMB 8826 / WCFS1) OX=220668 GN=rhaA PE=3 SV=1	0.5540 32683	0.00102 6	down
F9USJ9	lp_3084	Cell surface protein, ErfK family OS=Lactobacillus plantarum (strain ATCC BAA-793 / NCIMB 8826 / WCFS1) OX=220668 GN=lp_3084 PE=4 SV=1	0.4490 16716	0.00112 7	down
F9UTV9	Biotin carboxyl carrier protein of acetyl-CoA carboxylase	Biotin carboxyl carrier protein of acetyl-CoA carboxylase OS=Lactobacillus plantarum (strain ATCC BAA-793 / NCIMB 8826 / WCFS1) OX=220668 GN=accB3 PE=4 SV=1	0.5520 33809	4.29E-0 5	down
Q6LWD8	Mobilization protein	Uncharacterized protein OS=Lactobacillus plantarum (strain ATCC BAA-793 / NCIMB 8826 / WCFS1) OX=220668 GN=orf39 PE=4 SV=1	0.4965 32381	0.00086 44	down
F9UQY6	lp_2447	Prophage P2a protein 10, phage transcription regulator, Cro/CI family OS=Lactobacillus plantarum (strain ATCC BAA-793 / NCIMB 8826 / WCFS1) OX=220668 GN=lp_2447 PE=4 SV=1	0.5287 6569	0.00073 91	down
F9UPN8	lp_1915	Lipoprotein OS=Lactobacillus plantarum (strain ATCC BAA-793 / NCIMB 8826 / WCFS1) OX=220668 GN=lp_1915 PE=4 SV=1	0.5109 9015	0.00077 46	down
F9UTP5	lp_0290	Transcriptional attenuator, cell envelope-related, LytR family OS=Lactobacillus plantarum (strain ATCC BAA-793 / NCIMB 8826 / WCFS1) OX=220668 GN=lp_0290 PE=3 SV=1	0.5010 30928	0.00017 9	down
F9UT63	DUF4123 domain-containing protein	Uncharacterized protein OS=Lactobacillus plantarum (strain ATCC BAA-793 / NCIMB 8826 / WCFS1) OX=220668 GN=lp_0266 PE=4 SV=1	0.5338 62434	7.47E-0 5	down
F9URK4	N5-carboxyaminoimidazole ribonucleotide synthase	N5-carboxyaminoimidazole ribonucleotide synthase OS=Lactobacillus plantarum (strain ATCC BAA-793 / NCIMB 8826 / WCFS1) OX=220668 GN=purK1 PE=3 SV=1	0.5079 45635	0.00049 71	down
F9UTM2	Adenine DNA glycosylase	Adenine DNA glycosylase OS=Lactobacillus plantarum (strain ATCC BAA-793 / NCIMB 8826 / WCFS1) OX=220668 GN=mutY PE=3 SV=1	0.6598 95531	0.00271 3	down
F9UT96	lp_3185	Branched-chain amino acid transport protein OS=Lactobacillus plantarum (strain ATCC BAA-793 / NCIMB 8826 / WCFS1) OX=220668 GN=lp_3185 PE=3 SV=1	0.5138 00425	0.00077 77	down
Q88VJ7	Ribosome-recycling factor	Ribosome-recycling factor OS=Lactobacillus plantarum (strain ATCC BAA-793 / NCIMB 8826 / WCFS1) OX=220668 GN=frr PE=3 SV=1	0.5639 47078	7.54E-0 5	down
F9UQ26	lp_2075	Transcriptional attenuator, cell envelope-related, LytR family OS=Lactobacillus plantarum (strain ATCC BAA-793 / NCIMB 8826 / WCFS1) OX=220668 GN=lp_2075 PE=3 SV=1	0.5011 68348	0.00080 3	down
F9UQH8	lp_2259	Transcription regulator, MerR family OS=Lactobacillus plantarum (strain ATCC BAA-793 / NCIMB 8826 / WCFS1) OX=220668 GN=lp_2259 PE=4 SV=1	0.5941 20972	0.00397 9	down
F9US19	lp_2934	Lipoprotein OS=Lactobacillus plantarum (strain ATCC BAA-793 / NCIMB 8826 / WCFS1) OX=220668 GN=lp_2934 PE=4 SV=1	0.5598 21925	0.00027 9	down

F9US02	lp_2914	Hypothetical membrane protein, DUF2207 family OS=Lactobacillus plantarum (strain ATCC BAA-793 / NCIMB 8826 / WCFS1) OX=220668 GN=lp_2914 PE=4 SV=1	0.591685649	0.0005384	down
F9UM21	lp_0800	Cell surface protein, LPXTG-motif cell wall anchor OS=Lactobacillus plantarum (strain ATCC BAA-793 / NCIMB 8826 / WCFS1) OX=220668 GN=lp_0800 PE=4 SV=1	0.590210586	0.001563	down
F9UQ45	lp_2098	Metallophosphoesterase, lipoprotein OS=Lactobacillus plantarum (strain ATCC BAA-793 / NCIMB 8826 / WCFS1) OX=220668 GN=lp_2098 PE=4 SV=1	0.528987899	0.0003326	down
F9URC0	lp_2616	Bacteriocin immunity protein OS=Lactobacillus plantarum (strain ATCC BAA-793 / NCIMB 8826 / WCFS1) OX=220668 GN=lp_2616 PE=4 SV=1	0.489908749	7.55E-05	down
F9ULD4	spx5	RNA polymerase (RNAP)-binding regulatory protein, arsenate reductase (ArsC) family, Spx subfamily OS=Lactobacillus plantarum (strain ATCC BAA-793 / NCIMB 8826 / WCFS1) OX=220668 GN=spx5 PE=3 SV=1	0.519188596	1.10E-05	down
F9UMT1	lp_1124	Cell surface hydrolase, LPXTG-motif cell wall anchor OS=Lactobacillus plantarum (strain ATCC BAA-793 / NCIMB 8826 / WCFS1) OX=220668 GN=lp_1124 PE=4 SV=1	0.588742102	0.0001288	down
F9UTD9	lp_3239	Short-chain dehydrogenase/oxidoreductase,atypical SDR family, TMR-like OS=Lactobacillus plantarum (strain ATCC BAA-793 / NCIMB 8826 / WCFS1) OX=220668 GN=lp_3239 PE=4 SV=1	0.639004149	0.003985	down
F9US78	lp_0058	Flavoprotein OS=Lactobacillus plantarum (strain ATCC BAA-793 / NCIMB 8826 / WCFS1) OX=220668 GN=lp_0058 PE=4 SV=1	0.531170272	2.05E-05	down
F9UQL7	lp_2306	Non-proteolytic protein, peptidase family M16 OS=Lactobacillus plantarum (strain ATCC BAA-793 / NCIMB 8826 / WCFS1) OX=220668 GN=lp_2306 PE=4 SV=1	0.651079137	0.0007906	down
F9UQC5	Cell division protein DivIB	Cell division protein DivIB OS=Lactobacillus plantarum (strain ATCC BAA-793 / NCIMB 8826 / WCFS1) OX=220668 GN=divIB PE=3 SV=1	0.61845973	0.0001458	down
F9UPQ6	lp_1935	Cell surface hydrolase, membrane-bound OS=Lactobacillus plantarum (strain ATCC BAA-793 / NCIMB 8826 / WCFS1) OX=220668 GN=lp_1935 PE=4 SV=1	0.557426304	0.007016	down
Q88Z77	Probable DNA-directed RNA polymerase subunit delta	Probable DNA-directed RNA polymerase subunit delta OS=Lactobacillus plantarum (strain ATCC BAA-793 / NCIMB 8826 / WCFS1) OX=220668 GN=rpoE PE=3 SV=1	0.55570051	2.01E-05	down
F9UPA0	lp_1746	D-methionine ABC transporter, substrate binding protein OS=Lactobacillus plantarum (strain ATCC BAA-793 / NCIMB 8826 / WCFS1) OX=220668 GN=lp_1746 PE=3 SV=1	0.539554795	0.0005363	down
F9UT67	Flavin prenyltransferase LpdB	Flavin prenyltransferase LpdB OS=Lactobacillus plantarum (strain ATCC BAA-793 / NCIMB 8826 / WCFS1) OX=220668 GN=lpdB PE=3 SV=1	0.603448276	0.0002412	down
F9URZ6	endA	DNA-entry nuclease OS=Lactobacillus plantarum (strain ATCC BAA-793 / NCIMB 8826 / WCFS1) OX=220668 GN=endA PE=4 SV=1	0.59384251	0.001696	down
F9US28	zinc_ribbon_2 domain-containing protein	zinc_ribbon_2 domain-containing protein OS=Lactobacillus plantarum (strain ATCC BAA-793 / NCIMB 8826 / WCFS1) OX=220668 GN=lp_2948 PE=4 SV=1	0.61668682	0.0004052	down
Q88V12	UPF0297 protein lp_2275	UPF0297 protein lp_2275 OS=Lactobacillus plantarum (strain ATCC BAA-793 / NCIMB 8826 / WCFS1) OX=220668 GN=lp_2275 PE=3 SV=1	0.653846154	0.0005229	down
F9UL67	zmp2	Extracellular zinc metalloproteinase, M10 family OS=Lactobacillus plantarum (strain ATCC BAA-793 / NCIMB 8826 / WCFS1) OX=220668 GN=zmp2 PE=4 SV=1	0.638733706	0.0005751	down
F9US12	lp_2925	Cell surface protein, LPXTG-motif cell wall anchor OS=Lactobacillus plantarum (strain ATCC BAA-793 / NCIMB 8826 / WCFS1) OX=220668 GN=lp_2925 PE=4 SV=1	0.65681961	0.00659	down
F9UQY4	lp_2445	Prophage P2a protein 12 OS=Lactobacillus plantarum (strain ATCC BAA-793 / NCIMB 8826 / WCFS1) OX=220668 GN=lp_2445 PE=4 SV=1	0.572188995	0.001011	down
F9URF9	Type I site-specific deoxyribonuclease	Uncharacterized protein OS=Lactobacillus plantarum (strain ATCC BAA-793 / NCIMB 8826 / WCFS1) OX=220668 GN=lp_2669 PE=4 SV=1	0.578725962	0.0003711	down

F9UU09	plnG	Bacteriocin ABC-transporter, ATP-binding and permease protein PlnG OS=Lactobacillus plantarum (strain ATCC BAA-793 / NCIMB 8826 / WCFS1) OX=220668 GN=plnG PE=4 SV=1	0.6308266	4.51E-05	down
P71479	Bifunctional protein PyrR 1	Bifunctional protein PyrR 1 OS=Lactobacillus plantarum (strain ATCC BAA-793 / NCIMB 8826 / WCFS1) OX=220668 GN=pyrR1 PE=3 SV=1	0.505912744	0.0001555	down
F9UTL5	lp_3341	Cell surface hydrolase, DUF915 family,membrane-bound OS=Lactobacillus plantarum (strain ATCC BAA-793 / NCIMB 8826 / WCFS1) OX=220668 GN=lp_3341 PE=4 SV=1	0.566425558	0.002069	down
Q88YW7	50S ribosomal protein L7/L12	50S ribosomal protein L7/L12 OS=Lactobacillus plantarum (strain ATCC BAA-793 / NCIMB 8826 / WCFS1) OX=220668 GN=rplL PE=3 SV=1	0.616241444	0.0001548	down
F9UPF6	DUF4192 family protein	Uncharacterized protein OS=Lactobacillus plantarum (strain ATCC BAA-793 / NCIMB 8826 / WCFS1) OX=220668 GN=lp_1813 PE=4 SV=1	0.669027688	0.006806	down
F9UPA5	pbp1A	DD-transpeptidase OS=Lactobacillus plantarum (strain ATCC BAA-793 / NCIMB 8826 / WCFS1) OX=220668 GN=pbp1A PE=4 SV=1	0.630749842	0.0002618	down
F9UNT6	lp_1556	Metal-dependent phosphohydrolase, HD family OS=Lactobacillus plantarum (strain ATCC BAA-793 / NCIMB 8826 / WCFS1) OX=220668 GN=lp_1556 PE=4 SV=1	0.534009547	0.0009888	down
F9USD6	pts23B	PTS system, cellobiose-specific EIIB component OS=Lactobacillus plantarum (strain ATCC BAA-793 / NCIMB 8826 / WCFS1) OX=220668 GN=pts23B PE=4 SV=1	0.597199751	6.44E-05	down
Q88VL9	Protein GrpE	Protein GrpE OS=Lactobacillus plantarum (strain ATCC BAA-793 / NCIMB 8826 / WCFS1) OX=220668 GN=grpE PE=3 SV=1	0.58707483	0.0001418	down
F9UUJ0	pbg10	6-phospho-beta-glucosidase OS=Lactobacillus plantarum (strain ATCC BAA-793 / NCIMB 8826 / WCFS1) OX=220668 GN=pbg10 PE=3 SV=1	0.650128866	0.001066	down
F9UPT6	GatB/YqeY domain-containing protein	Uncharacterized protein OS=Lactobacillus plantarum (strain ATCC BAA-793 / NCIMB 8826 / WCFS1) OX=220668 GN=lp_1972 PE=4 SV=1	0.626704908	0.0007672	down
Q88XP8	Aspartyl/glutamyl-tRNA(Asn/Gln) amidotransferase subunit C	Aspartyl/glutamyl-tRNA(Asn/Gln) amidotransferase subunit C OS=Lactobacillus plantarum (strain ATCC BAA-793 / NCIMB 8826 / WCFS1) OX=220668 GN=gatC PE=3 SV=1	0.644509044	0.001453	down
F9UR96	lp_2586	Cell surface hydrolase, DUF915 family,membrane-bound OS=Lactobacillus plantarum (strain ATCC BAA-793 / NCIMB 8826 / WCFS1) OX=220668 GN=lp_2586 PE=4 SV=1	0.588826467	7.60E-05	down
F9UM28	tRNA threonylcarbamoyladenosine biosynthesis protein TsaE	t(6)A37 threonylcarbamoyladenosine biosynthesis protein TsaE OS=Lactobacillus plantarum (strain ATCC BAA-793 / NCIMB 8826 / WCFS1) OX=220668 GN=lp_0809 PE=3 SV=1	0.629276105	2.16E-05	down
F9ULM1	lp_3678	Cell surface protein, CscA/DUF916 family OS=Lactobacillus plantarum (strain ATCC BAA-793 / NCIMB 8826 / WCFS1) OX=220668 GN=lp_3678 PE=4 SV=1	0.623642173	0.003138	down
F9UU92	lp_3413	Cell surface protein, CscA/DUF916 family OS=Lactobacillus plantarum (strain ATCC BAA-793 / NCIMB 8826 / WCFS1) OX=220668 GN=lp_3413 PE=4 SV=1	0.645838752	0.001041	down
F9UN64	dacA2	Serine-type D-Ala-D-Ala carboxypeptidase OS=Lactobacillus plantarum (strain ATCC BAA-793 / NCIMB 8826 / WCFS1) OX=220668 GN=dacA2 PE=3 SV=1	0.554484029	0.0005541	down
F9UR61	lp_2541	ABC transporter, substrate binding protein OS=Lactobacillus plantarum (strain ATCC BAA-793 / NCIMB 8826 / WCFS1) OX=220668 GN=lp_2541 PE=4 SV=1	0.622001283	0.0005657	down
P77886	Carbamoyl-phosphate synthase pyrimidine-specific large chain	Carbamoyl-phosphate synthase pyrimidine-specific large chain OS=Lactobacillus plantarum (strain ATCC BAA-793 / NCIMB 8826 / WCFS1) OX=220668 GN=pyrAB PE=3 SV=2	0.647058824	9.72E-05	down

F9UQX4	lp_2434	Prophage P2a protein 23 OS=Lactobacillus plantarum (strain ATCC BAA-793 / NCIMB 8826 / WCFS1) OX=220668 GN=lp_2434 PE=4 SV=1	0.6513 1406	0.00259 5	down
F9UMN8	lp_1070	Lipoprotein, FMN-binding protein OS=Lactobacillus plantarum (strain ATCC BAA-793 / NCIMB 8826 / WCFS1) OX=220668 GN=lp_1070 PE=4 SV=1	0.6385 4712	0.00055 04	down
F9UMC9	asp2	Alkaline shock protein OS=Lactobacillus plantarum (strain ATCC BAA-793 / NCIMB 8826 / WCFS1) OX=220668 GN=asp2 PE=3 SV=1	0.6432 32589	1.72E-0 5	down
F9UMW3	Universal stress protein	Universal stress protein OS=Lactobacillus plantarum (strain ATCC BAA-793 / NCIMB 8826 / WCFS1) OX=220668 GN=lp_1163 PE=1 SV=1	0.6677 61394	0.00202 7	down
F9UTB9	Zinc_ribb on_2 domain-c ontaining protein	Uncharacterized protein OS=Lactobacillus plantarum (strain ATCC BAA-793 / NCIMB 8826 / WCFS1) OX=220668 GN=lp_3215 PE=4 SV=1	0.6689 09825	0.00147 7	down
F9USC3	lp_2993	Nucleotide-binding protein, universal stress protein UspA family OS=Lactobacillus plantarum (strain ATCC BAA-793 / NCIMB 8826 / WCFS1) OX=220668 GN=lp_2993 PE=3 SV=1	0.6513 65756	0.00043 61	down
F9URL1	lp_2737	Cell surface hydrolase, DUF915 family,membrane-bound OS=Lactobacillus plantarum (strain ATCC BAA-793 / NCIMB 8826 / WCFS1) OX=220668 GN=lp_2737 PE=4 SV=1	0.4977 54854	0.00133 4	down
F9UR02	lp_2467	Prophage P2b protein 14, terminase small subunit OS=Lactobacillus plantarum (strain ATCC BAA-793 / NCIMB 8826 / WCFS1) OX=220668 GN=lp_2467 PE=4 SV=1	0.6655 40541	0.00383 1	down
F9UUC6	lp_3453	Cell surface protein, CscB family OS=Lactobacillus plantarum (strain ATCC BAA-793 / NCIMB 8826 / WCFS1) OX=220668 GN=lp_3453 PE=4 SV=1	0.6366 06546	0.00297 9	down
Q88YP9	Nucleoid-associated protein lp_0699	Nucleoid-associated protein lp_0699 OS=Lactobacillus plantarum (strain ATCC BAA-793 / NCIMB 8826 / WCFS1) OX=220668 GN=lp_0699 PE=3 SV=1	0.5791 07374	0.00075 21	down
F9ULS6	lp_0689	Cell surface protein, lipoprotein OS=Lactobacillus plantarum (strain ATCC BAA-793 / NCIMB 8826 / WCFS1) OX=220668 GN=lp_0689 PE=4 SV=1	0.6506 43196	0.00357 7	down
Q88YG1	Uracil-D NA glycosylas e	Uracil-DNA glycosylase OS=Lactobacillus plantarum (strain ATCC BAA-793 / NCIMB 8826 / WCFS1) OX=220668 GN=ung PE=3 SV=1	0.6347 26905	0.02775	down
F9ULS5	lp_0688	DNA entry nuclease OS=Lactobacillus plantarum (strain ATCC BAA-793 / NCIMB 8826 / WCFS1) OX=220668 GN=lp_0688 PE=4 SV=1	0.6581 49171	0.00165 6	down
F9USJ3	lp_3077	Extracellular protein OS=Lactobacillus plantarum (strain ATCC BAA-793 / NCIMB 8826 / WCFS1) OX=220668 GN=lp_3077 PE=4 SV=1	0.5899 31507	0.00306 8	down
F9ULW6	Ribosome hibernatio n promotin g factor	Ribosome hibernation promoting factor OS=Lactobacillus plantarum (strain ATCC BAA-793 / NCIMB 8826 / WCFS1) OX=220668 GN=hpf PE=3 SV=1	0.6252 10674	0.00077 17	down
F9UNN3	Molybdo pterin molybden umtransfe rase	Molybdopterin molybdenumtransferase OS=Lactobacillus plantarum (strain ATCC BAA-793 / NCIMB 8826 / WCFS1) OX=220668 GN=moeA PE=3 SV=1	0.5815 61822	0.00889 4	down
F9UMH9	lp_0992	Transcription regulator, MerR family OS=Lactobacillus plantarum (strain ATCC BAA-793 / NCIMB 8826 / WCFS1) OX=220668 GN=lp_0992 PE=4 SV=1	1.7172 53474	0.02091	up
F9UUC2	nox5	NADH oxidase OS=Lactobacillus plantarum (strain ATCC BAA-793 / NCIMB 8826 / WCFS1) OX=220668 GN=nox5 PE=4 SV=1	1.6173 59573	0.01738	up
F9UUG8	lp_3502	Transcription regulator, LysR family OS=Lactobacillus plantarum (strain ATCC BAA-793 / NCIMB 8826 / WCFS1) OX=220668 GN=lp_3502 PE=3 SV=1	1.6203 50577	0.00682 1	up
F9UPD3	pgm5	Phosphoglycerate mutase family protein OS=Lactobacillus plantarum (strain ATCC BAA-793 / NCIMB 8826 / WCFS1) OX=220668 GN=pgm5 PE=4 SV=1	1.5670 04424	0.00132 4	up
Q88WJ3	Ribosome maturatio n factor RimM	Ribosome maturation factor RimM OS=Lactobacillus plantarum (strain ATCC BAA-793 / NCIMB 8826 / WCFS1) OX=220668 GN=rimM PE=3 SV=1	1.5952 0716	0.00146 9	up
F9UL55	manR	Sigma54 activator, mannose PTS operon regulator OS=Lactobacillus plantarum (strain ATCC BAA-793 / NCIMB 8826 / WCFS1) OX=220668 GN=manR PE=4 SV=1	1.6766 46707	0.00166 8	up

F9UNL1	sufU	SUF system FeS assembly protein, NifU family OS=Lactobacillus plantarum (strain ATCC BAA-793 / NCIMB 8826 / WCFS1) OX=220668 GN=sufU PE=4 SV=1	1.535043715	0.0005767	up
F9UQB3	Cysteine desulfurase	Cysteine desulfurase OS=Lactobacillus plantarum (strain ATCC BAA-793 / NCIMB 8826 / WCFS1) OX=220668 GN=iscS PE=3 SV=1	2.139179833	0.003376	up
F9UPE3	lp_1796	DegV family protein OS=Lactobacillus plantarum (strain ATCC BAA-793 / NCIMB 8826 / WCFS1) OX=220668 GN=lp_1796 PE=4 SV=1	1.777568035	0.001668	up
F9URK6	lp_2732	NADPH-dependent FMN reductase family protein OS=Lactobacillus plantarum (strain ATCC BAA-793 / NCIMB 8826 / WCFS1) OX=220668 GN=lp_2732 PE=4 SV=1	1.563828228	0.0003465	up
F9UM73	AP2/ERF domain-containing protein	AP2/ERF domain-containing protein OS=Lactobacillus plantarum (strain ATCC BAA-793 / NCIMB 8826 / WCFS1) OX=220668 GN=lp_0862 PE=4 SV=1	1.654490107	0.0006611	up
F9UMU5	ATP-dependent DNA helicase	ATP-dependent DNA helicase OS=Lactobacillus plantarum (strain ATCC BAA-793 / NCIMB 8826 / WCFS1) OX=220668 GN=pcrA PE=3 SV=1	1.546904727	0.0006034	up
F9UPK5	lp_1876	Hydrolase, HAD superfamily, Cof family OS=Lactobacillus plantarum (strain ATCC BAA-793 / NCIMB 8826 / WCFS1) OX=220668 GN=lp_1876 PE=4 SV=1	1.601018421	0.0003146	up
F9UQE1	lp_2217	Transport protein OS=Lactobacillus plantarum (strain ATCC BAA-793 / NCIMB 8826 / WCFS1) OX=220668 GN=lp_2217 PE=3 SV=1	1.631259484	0.0007066	up
F9URN4	lp_2763	Nucleotide-binding protein, histidine triad family OS=Lactobacillus plantarum (strain ATCC BAA-793 / NCIMB 8826 / WCFS1) OX=220668 GN=lp_2763 PE=4 SV=1	1.546391753	0.0003609	up
F9UR81	DUF1801 domain-containing protein	DUF1801 domain-containing protein OS=Lactobacillus plantarum (strain ATCC BAA-793 / NCIMB 8826 / WCFS1) OX=220668 GN=lp_2566 PE=4 SV=1	1.703598931	0.0008546	up
F9UPG9	lp_1833	Integrase, N-terminal SAM-like OS=Lactobacillus plantarum (strain ATCC BAA-793 / NCIMB 8826 / WCFS1) OX=220668 GN=lp_1833 PE=4 SV=1	1.519730087	0.0001939	up
F9UPK1	lp_1871	ABC transporter, ATP-binding protein, ChvD family OS=Lactobacillus plantarum (strain ATCC BAA-793 / NCIMB 8826 / WCFS1) OX=220668 GN=lp_1871 PE=4 SV=1	1.623644417	0.000219	up
F9UQ86	50S ribosomal subunit assembly factor BipA	50S ribosomal subunit assembly factor BipA OS=Lactobacillus plantarum (strain ATCC BAA-793 / NCIMB 8826 / WCFS1) OX=220668 GN=typA PE=3 SV=1	1.68310631	0.000348	up
F9UQN3	csd2	Cysteine desulfurase OS=Lactobacillus plantarum (strain ATCC BAA-793 / NCIMB 8826 / WCFS1) OX=220668 GN=csd2 PE=3 SV=1	1.59011672	0.00024	up
F9UQN5	lp_2330	Signaling protein containing GAF domain OS=Lactobacillus plantarum (strain ATCC BAA-793 / NCIMB 8826 / WCFS1) OX=220668 GN=lp_2330 PE=4 SV=1	1.502202643	0.0005045	up
F9USE0	lp_3013	Transcription regulator, MerR family OS=Lactobacillus plantarum (strain ATCC BAA-793 / NCIMB 8826 / WCFS1) OX=220668 GN=lp_3013 PE=4 SV=1	1.591917955	0.0002925	up
F9UT43	lp_0244	NADPH-dependent FMN reductase family protein OS=Lactobacillus plantarum (strain ATCC BAA-793 / NCIMB 8826 / WCFS1) OX=220668 GN=lp_0244 PE=4 SV=1	1.655421309	0.000279	up
F9URK8	Lipoate--protein ligase	Lipoate--protein ligase OS=Lactobacillus plantarum (strain ATCC BAA-793 / NCIMB 8826 / WCFS1) OX=220668 GN=lplA2 PE=4 SV=1	1.573820396	4.52E-05	up
F9UPD7	DUF2087 domain-containing protein	DUF2087 domain-containing protein OS=Lactobacillus plantarum (strain ATCC BAA-793 / NCIMB 8826 / WCFS1) OX=220668 GN=lp_1789 PE=4 SV=1	1.638832527	0.0005416	up
F9UUC4	lp_3451	Cell surface protein, CscA/DUF916 family OS=Lactobacillus plantarum (strain ATCC BAA-793 / NCIMB 8826 / WCFS1) OX=220668 GN=lp_3451 PE=4 SV=1	3.099684696	0.01547	up
F9URK7	lp_2733	NADPH-dependent FMN reductase family protein OS=Lactobacillus plantarum (strain ATCC BAA-793 / NCIMB 8826 / WCFS1) OX=220668 GN=lp_2733 PE=4 SV=1	1.569254186	4.18E-05	up
F9ULR9	lp_0681	Prophage P1 protein 58, lysin OS=Lactobacillus plantarum (strain ATCC BAA-793 / NCIMB 8826 / WCFS1) OX=220668 GN=lp_0681 PE=3 SV=1	1.813930182	0.0007245	up

Q88WM7	Exodeoxy ribonucle ase 7 large subunit	Exodeoxyribonuclease 7 large subunit OS=Lactobacillus plantarum (strain ATCC BAA-793 / NCIMB 8826 / WCFS1) OX=220668 GN=xseA PE=3 SV=1	1.5995 05944	9.19E-0 5	up
F9UQA4	TPR_REG ION domain-c ontaining protein	Uncharacterized protein OS=Lactobacillus plantarum (strain ATCC BAA-793 / NCIMB 8826 / WCFS1) OX=220668 GN=lp_2169 PE=4 SV=1	1.5097 71744	3.50E-0 6	up
F9US91	lp_0073	Glutamine amidotransferase class-I OS=Lactobacillus plantarum (strain ATCC BAA-793 / NCIMB 8826 / WCFS1) OX=220668 GN=lp_0073 PE=4 SV=1	1.5829 8787	5.09E-0 5	up
F9UT86	lp_3173	Cell surface protein, membrane-anchored OS=Lactobacillus plantarum (strain ATCC BAA-793 / NCIMB 8826 / WCFS1) OX=220668 GN=lp_3173 PE=4 SV=1	1.5523 52049	0.00093 89	up
F9UUB9	lp_3444	Transcription regulator, Crp family OS=Lactobacillus plantarum (strain ATCC BAA-793 / NCIMB 8826 / WCFS1) OX=220668 GN=lp_3444 PE=4 SV=1	1.5877 90967	0.00026 13	up
Q88YZ2	Acetyl-co enzyme A carboxyla se carboxyl transferas e subunit beta 1	Acetyl-coenzyme A carboxylase carboxyl transferase subunit beta 1 OS=Lactobacillus plantarum (strain ATCC BAA-793 / NCIMB 8826 / WCFS1) OX=220668 GN=accD1 PE=3 SV=1	1.7772 62949	0.00025 94	up
Q88YI8	UvrABC system protein B	UvrABC system protein B OS=Lactobacillus plantarum (strain ATCC BAA-793 / NCIMB 8826 / WCFS1) OX=220668 GN=uvrB PE=3 SV=1	1.6171 875	4.38E-0 5	up
F9UPF1	DUF960 domain-c ontaining protein	Uncharacterized protein OS=Lactobacillus plantarum (strain ATCC BAA-793 / NCIMB 8826 / WCFS1) OX=220668 GN=lp_1806 PE=4 SV=1	1.6980 8281	0.00013 38	up
F9UPH5	DNA topoisom erase 4 subunit A	DNA topoisomerase 4 subunit A OS=Lactobacillus plantarum (strain ATCC BAA-793 / NCIMB 8826 / WCFS1) OX=220668 GN=parC PE=3 SV=1	1.6421 91694	8.97E-0 5	up
F9UTE3	lp_3244	NADPH-dependent FMN reductase family protein OS=Lactobacillus plantarum (strain ATCC BAA-793 / NCIMB 8826 / WCFS1) OX=220668 GN=lp_3244 PE=4 SV=1	1.6759 95522	0.00025 26	up
F9UT31	Dipeptida se	Dipeptidase OS=Lactobacillus plantarum (strain ATCC BAA-793 / NCIMB 8826 / WCFS1) OX=220668 GN=pepD1 PE=3 SV=1	1.5849 69847	9.04E-0 6	up
F9UL78	lp_0613	Nuclease, NYN_YacP family OS=Lactobacillus plantarum (strain ATCC BAA-793 / NCIMB 8826 / WCFS1) OX=220668 GN=lp_0613 PE=4 SV=1	1.5119 60283	4.82E-0 5	up
F9UPK6	TPR_REG ION domain-c ontaining protein	Uncharacterized protein OS=Lactobacillus plantarum (strain ATCC BAA-793 / NCIMB 8826 / WCFS1) OX=220668 GN=lp_1877 PE=4 SV=1	1.6835 83051	7.71E-0 5	up
F9UPG3	lp_1820	Acetyltransferase, GNAT family OS=Lactobacillus plantarum (strain ATCC BAA-793 / NCIMB 8826 / WCFS1) OX=220668 GN=lp_1820 PE=4 SV=1	1.6480 89172	0.00012 78	up
F9UP88	Phospho mevalona te kinase	Phosphomevalonate kinase OS=Lactobacillus plantarum (strain ATCC BAA-793 / NCIMB 8826 / WCFS1) OX=220668 GN=mvaK2 PE=4 SV=1	1.5546 67077	9.02E-0 6	up
F9UR54	pts18CBA	PTS system, N-acetylglucosamine and glucose-specific EIICBA component OS=Lactobacillus plantarum (strain ATCC BAA-793 / NCIMB 8826 / WCFS1) OX=220668 GN=pts18CBA PE=4 SV=1	1.5719 37432	0.00020 98	up
F9URQ1	lp_2783	Glycosyltransferase, family 2 OS=Lactobacillus plantarum (strain ATCC BAA-793 / NCIMB 8826 / WCFS1) OX=220668 GN=lp_2783 PE=4 SV=1	1.5898 79744	0.00013 76	up
F9URJ0	NmrA domain-c ontaining protein	NmrA domain-containing protein OS=Lactobacillus plantarum (strain ATCC BAA-793 / NCIMB 8826 / WCFS1) OX=220668 GN=lp_2713 PE=4 SV=1	1.5319 99389	5.46E-0 7	up
F9US70	lp_0048	Uracil-DNA glycosylase family protein OS=Lactobacillus plantarum (strain ATCC BAA-793 / NCIMB 8826 / WCFS1) OX=220668 GN=lp_0048 PE=4 SV=1	1.7200 06565	7.36E-0 5	up
F9UM44	Maltose epimerase	Maltose epimerase OS=Lactobacillus plantarum (strain ATCC BAA-793 / NCIMB 8826 / WCFS1) OX=220668 GN=galM1 PE=3 SV=1	1.6054 82905	2.62E-0 5	up
F9UQP2	lp_2337	ATPase, AAA family OS=Lactobacillus plantarum (strain ATCC BAA-793 / NCIMB 8826 / WCFS1) OX=220668 GN=lp_2337 PE=3 SV=1	1.5205 12429	1.65E-0 6	up

F9UP44	Acetyl-CoA carboxytransferase	Acetyl-CoA carboxytransferase OS=Lactobacillus plantarum (strain ATCC BAA-793 / NCIMB 8826 / WCFS1) OX=220668 GN=accA2 PE=4 SV=1	1.650890422	2.53E-05	up
F9UM03	Putative gluconeogenesis factor	Putative gluconeogenesis factor OS=Lactobacillus plantarum (strain ATCC BAA-793 / NCIMB 8826 / WCFS1) OX=220668 GN=lp_0780 PE=1 SV=1	1.659416788	3.29E-05	up
F9UQA3	ATP-dependent RecD-like DNA helicase	ATP-dependent RecD-like DNA helicase OS=Lactobacillus plantarum (strain ATCC BAA-793 / NCIMB 8826 / WCFS1) OX=220668 GN=recD PE=3 SV=1	1.610759494	7.34E-06	up
F9UMN4	hepB1	Heptaprenyl diphosphate synthase component II OS=Lactobacillus plantarum (strain ATCC BAA-793 / NCIMB 8826 / WCFS1) OX=220668 GN=hepB1 PE=3 SV=1	1.550950982	2.10E-07	up
F9UUA8	lp_3430	Peroxidase OS=Lactobacillus plantarum (strain ATCC BAA-793 / NCIMB 8826 / WCFS1) OX=220668 GN=lp_3430 PE=4 SV=1	1.549535604	8.48E-05	up
F9US18	nrdD	Anaerobic ribonucleoside-triphosphate reductase OS=Lactobacillus plantarum (strain ATCC BAA-793 / NCIMB 8826 / WCFS1) OX=220668 GN=nrdD PE=4 SV=1	1.539873515	5.64E-05	up
Q88XY8	Elongation factor G	Elongation factor G OS=Lactobacillus plantarum (strain ATCC BAA-793 / NCIMB 8826 / WCFS1) OX=220668 GN=fusA PE=3 SV=1	1.503268968	3.28E-06	up
F9UPI1	Tyrosine recombinase XerC	Tyrosine recombinase XerC OS=Lactobacillus plantarum (strain ATCC BAA-793 / NCIMB 8826 / WCFS1) OX=220668 GN=xerC PE=3 SV=1	1.594637224	0.0001315	up
F9UUJ2	bglG5	Transcription antiterminator, BlgB family OS=Lactobacillus plantarum (strain ATCC BAA-793 / NCIMB 8826 / WCFS1) OX=220668 GN=bglG5 PE=4 SV=1	1.586477987	0.001402	up
F9UPE8	lp_1801	Galactose-1-phosphate uridylyltransferase, HIT family OS=Lactobacillus plantarum (strain ATCC BAA-793 / NCIMB 8826 / WCFS1) OX=220668 GN=lp_1801 PE=4 SV=1	1.633829889	9.13E-06	up
F9USF1	lp_3024	Transcription regulator, LysR family OS=Lactobacillus plantarum (strain ATCC BAA-793 / NCIMB 8826 / WCFS1) OX=220668 GN=lp_3024 PE=4 SV=1	1.581592587	5.18E-06	up
F9UMB1	DNA helicase	DNA helicase OS=Lactobacillus plantarum (strain ATCC BAA-793 / NCIMB 8826 / WCFS1) OX=220668 GN=lp_0910 PE=1 SV=1	1.548006825	7.25E-07	up
F9UN85	tagE3	Poly(Glycerol-phosphate) alpha-glucosyltransferase OS=Lactobacillus plantarum (strain ATCC BAA-793 / NCIMB 8826 / WCFS1) OX=220668 GN=tagE3 PE=4 SV=1	1.653466947	6.24E-06	up
F9UNZ1	16S rRNA (cytosine(967)-C(5))-methyltransferase	16S rRNA m5C967 methyltransferase OS=Lactobacillus plantarum (strain ATCC BAA-793 / NCIMB 8826 / WCFS1) OX=220668 GN=sunL PE=3 SV=1	1.567457201	7.15E-07	up
Q88VK3	Ribosome maturation factor RimP	Ribosome maturation factor RimP OS=Lactobacillus plantarum (strain ATCC BAA-793 / NCIMB 8826 / WCFS1) OX=220668 GN=rimP PE=3 SV=1	1.800582491	0.0001397	up
F9UU51	Bacteriocin immunity protein	Uncharacterized protein OS=Lactobacillus plantarum (strain ATCC BAA-793 / NCIMB 8826 / WCFS1) OX=220668 GN=lp_3366 PE=4 SV=1	1.505442281	0.004137	up
Q88W23	Methylenetetrahydrofolate--tRNA-(uracil-5-)-methyltransferase TrmFO	Methylenetetrahydrofolate--tRNA-(uracil-5-)-methyltransferase TrmFO OS=Lactobacillus plantarum (strain ATCC BAA-793 / NCIMB 8826 / WCFS1) OX=220668 GN=trmFO PE=3 SV=1	1.591305727	5.98E-05	up
F9URG1	nrdI	Ribonucleotide reductase protein NrdI OS=Lactobacillus plantarum (strain ATCC BAA-793 / NCIMB 8826 / WCFS1) OX=220668 GN=nrdI PE=4 SV=1	1.695558858	2.56E-05	up
F9USZ8	Sucrose-6-phosphate hydrolase	Sucrose-6-phosphate hydrolase OS=Lactobacillus plantarum (strain ATCC BAA-793 / NCIMB 8826 / WCFS1) OX=220668 GN=scrB PE=3 SV=1	1.572915024	1.80E-05	up
F9UP74	Diaminopimelate decarboxy	Diaminopimelate decarboxylase OS=Lactobacillus plantarum (strain ATCC BAA-793 / NCIMB 8826 / WCFS1) OX=220668 GN=lysA PE=3 SV=1	1.991922159	0.0002723	up

	lase				
Q88VS1	DNA repair protein RecO	DNA repair protein RecO OS=Lactobacillus plantarum (strain ATCC BAA-793 / NCIMB 8826 / WCFS1) OX=220668 GN=recO PE=3 SV=1	1.75257732	2.22E-05	up
Q88WG8	Beta-ketoacyl-[acyl-carrier-protein] synthase III 2	3-oxoacyl-[acyl-carrier-protein] synthase 3 protein 2 OS=Lactobacillus plantarum (strain ATCC BAA-793 / NCIMB 8826 / WCFS1) OX=220668 GN=fabH2 PE=3 SV=1	1.510566096	0.0002734	up
F9UP42	accC2	Acetyl-CoA carboxylase, biotin carboxylase subunit OS=Lactobacillus plantarum (strain ATCC BAA-793 / NCIMB 8826 / WCFS1) OX=220668 GN=accC2 PE=4 SV=1	1.582539683	4.79E-06	up
F9UQJ1	zapA	Cell-division Z-ring component, stimulator of FtsZ polymerization OS=Lactobacillus plantarum (strain ATCC BAA-793 / NCIMB 8826 / WCFS1) OX=220668 GN=zapA PE=4 SV=1	1.510421491	0.0003306	up
F9UM45	Phage protein	Uncharacterized protein OS=Lactobacillus plantarum (strain ATCC BAA-793 / NCIMB 8826 / WCFS1) OX=220668 GN=lp_0827 PE=4 SV=1	1.703826955	0.0001415	up
F9URB3	lp_2606	NAD(P)-dependent oxidoreductase OS=Lactobacillus plantarum (strain ATCC BAA-793 / NCIMB 8826 / WCFS1) OX=220668 GN=lp_2606 PE=4 SV=1	1.584752507	2.38E-07	up
F9ULR3	lp_0675	Prophage P1 protein 52, endolysin OS=Lactobacillus plantarum (strain ATCC BAA-793 / NCIMB 8826 / WCFS1) OX=220668 GN=lp_0675 PE=3 SV=1	1.580850725	0.003035	up
F9UNK2	DNA translocase FtsK	DNA translocase FtsK OS=Lactobacillus plantarum (strain ATCC BAA-793 / NCIMB 8826 / WCFS1) OX=220668 GN=ftsK1 PE=3 SV=1	1.624777039	2.02E-05	up
F9UQG0	lp_2235	Acid sugar phosphatase OS=Lactobacillus plantarum (strain ATCC BAA-793 / NCIMB 8826 / WCFS1) OX=220668 GN=lp_2235 PE=3 SV=1	1.524426409	1.73E-05	up
F9UP86	galM2	Aldose 1-epimerase OS=Lactobacillus plantarum (strain ATCC BAA-793 / NCIMB 8826 / WCFS1) OX=220668 GN=galM2 PE=4 SV=1	1.611147011	4.63E-06	up
F9UPJ2	lp_1860	Flavin monooxygenase, luciferase-like monooxygenase family OS=Lactobacillus plantarum (strain ATCC BAA-793 / NCIMB 8826 / WCFS1) OX=220668 GN=lp_1860 PE=4 SV=1	1.649180328	2.43E-05	up
F9UKW0	lp_0477	Lipoate-protein ligase A OS=Lactobacillus plantarum (strain ATCC BAA-793 / NCIMB 8826 / WCFS1) OX=220668 GN=lp_0477 PE=4 SV=1	1.552585798	0.0002795	up
F9UNA0	dgk2	Deoxynucleoside kinase OS=Lactobacillus plantarum (strain ATCC BAA-793 / NCIMB 8826 / WCFS1) OX=220668 GN=dgk2 PE=3 SV=1	1.697324415	1.38E-06	up
F9UMR2	Type I site-specific deoxyribonuclease	Uncharacterized protein OS=Lactobacillus plantarum (strain ATCC BAA-793 / NCIMB 8826 / WCFS1) OX=220668 GN=lp_1098 PE=4 SV=1	1.535062893	0.004395	up
Q88UZ7	DNA mismatch repair protein MutS	DNA mismatch repair protein MutS OS=Lactobacillus plantarum (strain ATCC BAA-793 / NCIMB 8826 / WCFS1) OX=220668 GN=mutS PE=3 SV=1	1.815481391	2.11E-05	up
F9UNT0	sbcC	Exonuclease SbcC OS=Lactobacillus plantarum (strain ATCC BAA-793 / NCIMB 8826 / WCFS1) OX=220668 GN=sbcC PE=4 SV=1	1.616046776	4.89E-07	up
F9UM19	Ribonuclease R	Ribonuclease R OS=Lactobacillus plantarum (strain ATCC BAA-793 / NCIMB 8826 / WCFS1) OX=220668 GN=rnr PE=3 SV=1	1.638538424	1.05E-07	up
F9URG5	lp_2677	Medium chain degydrogenase/reductase, MDR family OS=Lactobacillus plantarum (strain ATCC BAA-793 / NCIMB 8826 / WCFS1) OX=220668 GN=lp_2677 PE=4 SV=1	1.729693064	6.46E-06	up
Q88YL7	Protein translocase subunit SecA	Protein translocase subunit SecA OS=Lactobacillus plantarum (strain ATCC BAA-793 / NCIMB 8826 / WCFS1) OX=220668 GN=secA PE=3 SV=1	1.629393494	9.77E-07	up
F9UTF3	lp_3256	DegV family protein OS=Lactobacillus plantarum (strain ATCC BAA-793 / NCIMB 8826 / WCFS1) OX=220668 GN=lp_3256 PE=4 SV=1	1.637945183	1.03E-07	up
F9UQA9	ktrA	Potassium uptake protein OS=Lactobacillus plantarum (strain ATCC BAA-793 / NCIMB 8826 / WCFS1) OX=220668 GN=ktrA PE=4 SV=1	2.15324165	0.001161	up
F9USI6	lp_3069	Medium-chain degydrogenase/reductase, Qor family OS=Lactobacillus plantarum (strain ATCC BAA-793 / NCIMB 8826 / WCFS1) OX=220668 GN=lp_3069 PE=4 SV=1	1.562300319	0.0001943	up
F9UR87	Lipoprotein	Uncharacterized protein OS=Lactobacillus plantarum (strain ATCC BAA-793 / NCIMB 8826 / WCFS1) OX=220668 GN=lp_2573 PE=4 SV=1	1.626002947	8.93E-05	up
Q88ZU5	Phosphos	Phosphoserine aminotransferase OS=Lactobacillus plantarum (strain	1.5201	0.00010	up

	erine aminotransferase	ATCC BAA-793 / NCIMB 8826 / WCFS1) OX=220668 GN=serC PE=3 SV=1	94877	61	
F9UP26	lp_1660	Alcohol dehydrogenase, zinc-binding OS=Lactobacillus plantarum (strain ATCC BAA-793 / NCIMB 8826 / WCFS1) OX=220668 GN=lp_1660 PE=4 SV=1	1.694430422	6.47E-05	up
F9URG8	lp_2683	Aminotransferase with N-terminal regulator domain OS=Lactobacillus plantarum (strain ATCC BAA-793 / NCIMB 8826 / WCFS1) OX=220668 GN=lp_2683 PE=3 SV=1	1.656971913	0.0002526	up
Q88S59	Putative AgrB-like protein	Putative AgrB-like protein OS=Lactobacillus plantarum (strain ATCC BAA-793 / NCIMB 8826 / WCFS1) OX=220668 GN=lp_3582 PE=3 SV=1	1.597527249	0.0006674	up
Q88YI7	UvrABC system protein A	UvrABC system protein A OS=Lactobacillus plantarum (strain ATCC BAA-793 / NCIMB 8826 / WCFS1) OX=220668 GN=uvrA PE=3 SV=1	1.755567064	6.90E-06	up
Q88UZ8	DNA mismatch repair protein MutL	DNA mismatch repair protein MutL OS=Lactobacillus plantarum (strain ATCC BAA-793 / NCIMB 8826 / WCFS1) OX=220668 GN=mutL PE=3 SV=1	1.840840306	0.001914	up
F9UTE1	nth2	Endonuclease III OS=Lactobacillus plantarum (strain ATCC BAA-793 / NCIMB 8826 / WCFS1) OX=220668 GN=nth2 PE=4 SV=1	1.604374082	1.70E-05	up
F9UTF9	glyK	Glycerate kinase OS=Lactobacillus plantarum (strain ATCC BAA-793 / NCIMB 8826 / WCFS1) OX=220668 GN=glyK PE=3 SV=1	2.163394805	0.0002546	up
F9URR7	lp_2802	2,5 diketo-D-gluconic acid-like reductase NADP dependent (Promiscuous) OS=Lactobacillus plantarum (strain ATCC BAA-793 / NCIMB 8826 / WCFS1) OX=220668 GN=lp_2802 PE=4 SV=1	1.664105562	0.0001259	up
F9URP6	pbg4	6-phospho-beta-glucosidase OS=Lactobacillus plantarum (strain ATCC BAA-793 / NCIMB 8826 / WCFS1) OX=220668 GN=pbg4 PE=3 SV=1	1.523982903	0.0001193	up
Q88VP9	Ribosomal protein L11 methyltransferase	Ribosomal protein L11 methyltransferase OS=Lactobacillus plantarum (strain ATCC BAA-793 / NCIMB 8826 / WCFS1) OX=220668 GN=prmA PE=3 SV=1	1.928308824	4.38E-05	up
F9URQ2	pgm7	Phosphoglycerate mutase family protein OS=Lactobacillus plantarum (strain ATCC BAA-793 / NCIMB 8826 / WCFS1) OX=220668 GN=pgm7 PE=4 SV=1	1.511374408	0.0001488	up
F9US22	tRNA-dihydrouridine synthase	tRNA-dihydrouridine synthase OS=Lactobacillus plantarum (strain ATCC BAA-793 / NCIMB 8826 / WCFS1) OX=220668 GN=dus3 PE=3 SV=1	1.757765053	5.40E-06	up
F9USL2	lp_3098	NAD-dependent epimerase/dehydratase family protein OS=Lactobacillus plantarum (strain ATCC BAA-793 / NCIMB 8826 / WCFS1) OX=220668 GN=lp_3098 PE=4 SV=1	1.669411765	1.65E-05	up
F9UTP0	Transglycosylase	Uncharacterized protein OS=Lactobacillus plantarum (strain ATCC BAA-793 / NCIMB 8826 / WCFS1) OX=220668 GN=lp_0284 PE=4 SV=1	1.883636364	6.17E-05	up
F9URP7	pbg5	6-phospho-beta-glucosidase OS=Lactobacillus plantarum (strain ATCC BAA-793 / NCIMB 8826 / WCFS1) OX=220668 GN=pbg5 PE=3 SV=1	1.637514576	4.59E-06	up
F9USD8	pbg6	6-phospho-beta-glucosidase OS=Lactobacillus plantarum (strain ATCC BAA-793 / NCIMB 8826 / WCFS1) OX=220668 GN=pbg6 PE=3 SV=1	1.681864407	5.14E-05	up
F9ULT4	DNA polymerase III subunit gamma/tau	DNA polymerase III subunit gamma/tau OS=Lactobacillus plantarum (strain ATCC BAA-793 / NCIMB 8826 / WCFS1) OX=220668 GN=dnaX PE=3 SV=1	1.7135506	2.40E-06	up
F9UUF0	Beta-galactosidase	Beta-galactosidase OS=Lactobacillus plantarum (strain ATCC BAA-793 / NCIMB 8826 / WCFS1) OX=220668 GN=lacM PE=4 SV=1	1.680847458	3.62E-05	up
F9USZ3	mapB	Maltose phosphorylase OS=Lactobacillus plantarum (strain ATCC BAA-793 / NCIMB 8826 / WCFS1) OX=220668 GN=mapB PE=3 SV=1	1.551879942	0.0003046	up
Q88V96	tRNA-specific 2-thiouridylase MnmA	tRNA-specific 2-thiouridylase MnmA OS=Lactobacillus plantarum (strain ATCC BAA-793 / NCIMB 8826 / WCFS1) OX=220668 GN=mnmA PE=3 SV=1	1.760896202	2.13E-06	up
Q88WD0	Probable tautomerase lp_1712	Probable tautomerase lp_1712 OS=Lactobacillus plantarum (strain ATCC BAA-793 / NCIMB 8826 / WCFS1) OX=220668 GN=lp_1712 PE=3 SV=3	1.746114894	0.0001689	up
F9ULU2	Ribosomal RNA small	Ribosomal RNA small subunit methyltransferase I OS=Lactobacillus plantarum (strain ATCC BAA-793 / NCIMB 8826 / WCFS1) OX=220668 GN=rsmI PE=3 SV=1	1.575086164	0.0001623	up

	subunit methyltra nsferase I				
F9UTQ1	tag1	DNA-3-methyladenine glycosylase I OS=Lactobacillus plantarum (strain ATCC BAA-793 / NCIMB 8826 / WCFS1) OX=220668 GN=tag1 PE=4 SV=1	1.9144 98141	0.00029 46	up
F9UMI8	lp_1003	Acetyltransferase, GNAT family OS=Lactobacillus plantarum (strain ATCC BAA-793 / NCIMB 8826 / WCFS1) OX=220668 GN=lp_1003 PE=4 SV=1	1.7810 89232	2.79E-0 5	up
Q88V20	dITP/XTP pyrophos phatase	dITP/XTP pyrophosphatase OS=Lactobacillus plantarum (strain ATCC BAA-793 / NCIMB 8826 / WCFS1) OX=220668 GN=lp_2267 PE=3 SV=1	1.5957 23521	6.25E-0 5	up
F9UN47	SlpA domain-c ontaining protein	Uncharacterized protein OS=Lactobacillus plantarum (strain ATCC BAA-793 / NCIMB 8826 / WCFS1) OX=220668 GN=lp_1257 PE=4 SV=1	1.8341 48108	5.61E-0 6	up
F9UL62	Acetyl-Co A carboxytr ansferase	Acetyl-CoA carboxytransferase OS=Lactobacillus plantarum (strain ATCC BAA-793 / NCIMB 8826 / WCFS1) OX=220668 GN=accA1 PE=4 SV=1	1.5996 67774	4.48E-0 5	up
F9UL77	trmH	tRNA/rRNA methyltransferase, TrmH family OS=Lactobacillus plantarum (strain ATCC BAA-793 / NCIMB 8826 / WCFS1) OX=220668 GN=trmH PE=4 SV=1	1.8602 52424	3.87E-0 6	up
F9UP79	gabT	4-aminobutyrate aminotransferase OS=Lactobacillus plantarum (strain ATCC BAA-793 / NCIMB 8826 / WCFS1) OX=220668 GN=gabT PE=3 SV=1	1.5405 88905	0.00373	up
F9UPT9	lp_1975	Short-chain dehydrogenase/oxidoreductase OS=Lactobacillus plantarum (strain ATCC BAA-793 / NCIMB 8826 / WCFS1) OX=220668 GN=lp_1975 PE=4 SV=1	1.6462 71186	0.00036 79	up
Q88SI6	Demethyl menaquin one methyltra nsferase	Demethylmenaquinone methyltransferase OS=Lactobacillus plantarum (strain ATCC BAA-793 / NCIMB 8826 / WCFS1) OX=220668 GN=menG PE=3 SV=1	1.6908 62069	7.25E-0 6	up
F9UPS5	lp_1958	Acetoin ABC transporter, ATP-binding protein OS=Lactobacillus plantarum (strain ATCC BAA-793 / NCIMB 8826 / WCFS1) OX=220668 GN=lp_1958 PE=4 SV=1	1.6226 66891	4.25E-0 5	up
F9UQ72	holA	DNA-directed DNA polymerase III, delta chain OS=Lactobacillus plantarum (strain ATCC BAA-793 / NCIMB 8826 / WCFS1) OX=220668 GN=holA PE=4 SV=1	1.7146 09089	6.58E-0 6	up
F9UNS7	rrp5	Two-component system response regulator OS=Lactobacillus plantarum (strain ATCC BAA-793 / NCIMB 8826 / WCFS1) OX=220668 GN=rrp5 PE=4 SV=1	1.5382 10852	0.00059 97	up
F9UM42	lp_0824	Hydrolase, HAD superfamily, Cof family OS=Lactobacillus plantarum (strain ATCC BAA-793 / NCIMB 8826 / WCFS1) OX=220668 GN=lp_0824 PE=4 SV=1	1.5078 18797	0.00081 39	up
F9USF6	lp_3029	Short-chain dehydrogenase/oxidoreductase,atypical SDR family, subgroup 6 OS=Lactobacillus plantarum (strain ATCC BAA-793 / NCIMB 8826 / WCFS1) OX=220668 GN=lp_3029 PE=4 SV=1	1.5354 63884	0.00141 7	up
F9UNR2	lp_1527	Hydrolase, HAD superfamily OS=Lactobacillus plantarum (strain ATCC BAA-793 / NCIMB 8826 / WCFS1) OX=220668 GN=lp_1527 PE=4 SV=1	1.7337 78794	1.93E-0 5	up
F9URV3	lp_2851	Short-chain dehydrogenase/oxidoreductase OS=Lactobacillus plantarum (strain ATCC BAA-793 / NCIMB 8826 / WCFS1) OX=220668 GN=lp_2851 PE=3 SV=1	1.5989 6269	0.00062 64	up
Q88U29	Bifunction al purine biosynthe sis protein PurH	Bifunctional purine biosynthesis protein PurH OS=Lactobacillus plantarum (strain ATCC BAA-793 / NCIMB 8826 / WCFS1) OX=220668 GN=purH PE=3 SV=1	1.7642 86986	5.86E-0 6	up
Q88VM6	D-alanine --D-alanyl carrier protein ligase	D-alanine--D-alanyl carrier protein ligase OS=Lactobacillus plantarum (strain ATCC BAA-793 / NCIMB 8826 / WCFS1) OX=220668 GN=dltA PE=3 SV=1	1.7483 61962	3.56E-0 7	up
F9ULV5	lp_0723	ABC transporter, ATP-binding protein OS=Lactobacillus plantarum (strain ATCC BAA-793 / NCIMB 8826 / WCFS1) OX=220668 GN=lp_0723 PE=4 SV=1	1.9018 175	3.67E-0 6	up
F9ULS9	Ribonucle oside-dip hosphate	Ribonucleoside-diphosphate reductase OS=Lactobacillus plantarum (strain ATCC BAA-793 / NCIMB 8826 / WCFS1) OX=220668 GN=nrdF PE=3 SV=1	1.6475 11544	5.49E-0 5	up

	reductase				
F9ULD3	Catalase	Catalase OS=Lactobacillus plantarum (strain ATCC BAA-793 / NCIMB 8826 / WCFS1) OX=220668 GN=kat PE=3 SV=1	1.7057 69231	1.58E-0 5	up
F9ULL3	lp_3666	Aromatic compound hydratase/decarboxylase OS=Lactobacillus plantarum (strain ATCC BAA-793 / NCIMB 8826 / WCFS1) OX=220668 GN=lp_3666 PE=4 SV=1	1.5314 23895	0.00090 99	up
F9UTN9	rrp2	Two-component system response regulator OS=Lactobacillus plantarum (strain ATCC BAA-793 / NCIMB 8826 / WCFS1) OX=220668 GN=rrp2 PE=4 SV=1	1.6629 9294	0.00010 62	up
F9US31	lp_2953	Esterase OS=Lactobacillus plantarum (strain ATCC BAA-793 / NCIMB 8826 / WCFS1) OX=220668 GN=lp_2953 PE=4 SV=1	1.5872 51129	0.00197 1	up
F9ULW3	YigZ family protein	Uncharacterized protein OS=Lactobacillus plantarum (strain ATCC BAA-793 / NCIMB 8826 / WCFS1) OX=220668 GN=lp_0734 PE=3 SV=1	1.6031 98653	0.00024 53	up
F9UU28	tRNA-dih ydrouridi ne synthase	tRNA-dihydrouridine synthase OS=Lactobacillus plantarum (strain ATCC BAA-793 / NCIMB 8826 / WCFS1) OX=220668 GN=dus1 PE=3 SV=1	1.8444 64945	9.51E-0 5	up
F9UL60	accC1	Biotin carboxylase OS=Lactobacillus plantarum (strain ATCC BAA-793 / NCIMB 8826 / WCFS1) OX=220668 GN=accC1 PE=4 SV=1	1.8484 28835	2.84E-0 6	up
Q88VY8	Segregati on and condensat ion protein B	Segregation and condensation protein B OS=Lactobacillus plantarum (strain ATCC BAA-793 / NCIMB 8826 / WCFS1) OX=220668 GN=scpB PE=3 SV=1	1.7151 419	0.02191	up
Q88U30	Phosphori bosylami ne--glycin e ligase	Phosphoribosylamine--glycine ligase OS=Lactobacillus plantarum (strain ATCC BAA-793 / NCIMB 8826 / WCFS1) OX=220668 GN=purD PE=3 SV=1	1.5584 17816	0.00309 5	up
F9UPU8	lp_1985	Phosphohydrolase OS=Lactobacillus plantarum (strain ATCC BAA-793 / NCIMB 8826 / WCFS1) OX=220668 GN=lp_1985 PE=4 SV=1	1.6454 38898	8.06E-0 5	up
F9UT60	treA	Trehalose-6-phosphate hydrolase OS=Lactobacillus plantarum (strain ATCC BAA-793 / NCIMB 8826 / WCFS1) OX=220668 GN=treA PE=3 SV=1	1.9871 67023	1.25E-0 5	up
F9UN91	Pseudouri dine synthase	Pseudouridine synthase OS=Lactobacillus plantarum (strain ATCC BAA-793 / NCIMB 8826 / WCFS1) OX=220668 GN=rsuA PE=3 SV=1	1.7291 62964	9.09E-0 6	up
F9UKY3	L-serine dehydrata se	L-serine dehydratase OS=Lactobacillus plantarum (strain ATCC BAA-793 / NCIMB 8826 / WCFS1) OX=220668 GN=sdhB PE=3 SV=1	2.3720 62376	0.00024 59	up
F9UM35	lp_0816	Transcription regulator, MarR family OS=Lactobacillus plantarum (strain ATCC BAA-793 / NCIMB 8826 / WCFS1) OX=220668 GN=lp_0816 PE=4 SV=1	1.6071 42857	0.00028 11	up
Q88Z54	UDP-N-ac etylglucos amine 1-carboxy vinyltrans ferase 2	UDP-N-acetylglucosamine 1-carboxyvinyltransferase 2 OS=Lactobacillus plantarum (strain ATCC BAA-793 / NCIMB 8826 / WCFS1) OX=220668 GN=murA2 PE=3 SV=1	1.8857 57518	2.32E-0 7	up
F9UPH6	DNA topoisom erase 4 subunit B	DNA topoisomerase 4 subunit B OS=Lactobacillus plantarum (strain ATCC BAA-793 / NCIMB 8826 / WCFS1) OX=220668 GN=parE PE=3 SV=1	1.8147 59874	5.03E-0 6	up
F9ULL4	lp_3668	Acetyltransferase, GNAT family OS=Lactobacillus plantarum (strain ATCC BAA-793 / NCIMB 8826 / WCFS1) OX=220668 GN=lp_3668 PE=4 SV=1	1.5218 54745	0.00232 5	up
Q88YX4	50S ribosomal protein L33	50S ribosomal protein L33 OS=Lactobacillus plantarum (strain ATCC BAA-793 / NCIMB 8826 / WCFS1) OX=220668 GN=rpmG PE=3 SV=1	1.8504 60267	0.00041 03	up
F9ULU0	holB	DNA-directed DNA polymerase III, delta' subunit OS=Lactobacillus plantarum (strain ATCC BAA-793 / NCIMB 8826 / WCFS1) OX=220668 GN=holB PE=4 SV=1	2.0689 65517	2.23E-0 6	up
F9UPE1	Rqc2 homolog RqcH	Rqc2 homolog RqcH OS=Lactobacillus plantarum (strain ATCC BAA-793 / NCIMB 8826 / WCFS1) OX=220668 GN=rqcH PE=3 SV=1	2.0131 55272	1.94E-0 7	up
F9UTK3	lp_3324	Glycine betaine/carnitine/choline transport protein OS=Lactobacillus plantarum (strain ATCC BAA-793 / NCIMB 8826 / WCFS1) OX=220668 GN=lp_3324 PE=3 SV=1	1.7112 8656	0.00131 1	up
F9UPN7	lp_1914	Transcription regulator, MarR family OS=Lactobacillus plantarum	1.8202	0.00065	up

		(strain ATCC BAA-793 / NCIMB 8826 / WCFS1) OX=220668 GN=lp_1914 PE=4 SV=1	24719	18	
Q88WZ9	tRNA (guanine-N(7)-)-methyltransferase	tRNA (guanine-N(7)-)-methyltransferase OS=Lactobacillus plantarum (strain ATCC BAA-793 / NCIMB 8826 / WCFS1) OX=220668 GN=trmB PE=3 SV=1	1.8929 32794	1.12E-0 6	up
F9UQP6	lp_2342	Transcription regulator, Xre family OS=Lactobacillus plantarum (strain ATCC BAA-793 / NCIMB 8826 / WCFS1) OX=220668 GN=lp_2342 PE=4 SV=1	1.6563 77506	0.00065 85	up
F9UMI2	cspC	Cold shock protein CspC OS=Lactobacillus plantarum (strain ATCC BAA-793 / NCIMB 8826 / WCFS1) OX=220668 GN=cspC PE=4 SV=1	1.8181 81818	0.00150 8	up
F9USD4	lp_3006	Transcription regulator, TetR family OS=Lactobacillus plantarum (strain ATCC BAA-793 / NCIMB 8826 / WCFS1) OX=220668 GN=lp_3006 PE=4 SV=1	2.0426 82927	3.76E-0 6	up
Q88XQ4	Xanthine phosphoribosyltransferase	Xanthine phosphoribosyltransferase OS=Lactobacillus plantarum (strain ATCC BAA-793 / NCIMB 8826 / WCFS1) OX=220668 GN=xpt PE=3 SV=1	1.7129 10841	0.00057 31	up
F9ULT0	Ribonucleoside-diphosphate reductase	Ribonucleoside-diphosphate reductase OS=Lactobacillus plantarum (strain ATCC BAA-793 / NCIMB 8826 / WCFS1) OX=220668 GN=nrdE PE=3 SV=1	2.2973 27148	1.10E-0 5	up
Q88VL5	tRNA pseudouridine synthase B	tRNA pseudouridine synthase B OS=Lactobacillus plantarum (strain ATCC BAA-793 / NCIMB 8826 / WCFS1) OX=220668 GN=truB PE=3 SV=1	1.8775 35252	1.16E-0 5	up
F9ULI4	lp_3635	ABC transporter, ATP-binding protein, mannose related oligosaccharides OS=Lactobacillus plantarum (strain ATCC BAA-793 / NCIMB 8826 / WCFS1) OX=220668 GN=lp_3635 PE=4 SV=1	1.8256 23453	5.73E-0 5	up
F9UL40	tex	Transcription accessory protein, contains S1 RNA binding domain OS=Lactobacillus plantarum (strain ATCC BAA-793 / NCIMB 8826 / WCFS1) OX=220668 GN=tex PE=4 SV=1	1.8161 59696	0.00025 09	up
Q88VI7	UPF0291 protein lp_2062	UPF0291 protein lp_2062 OS=Lactobacillus plantarum (strain ATCC BAA-793 / NCIMB 8826 / WCFS1) OX=220668 GN=lp_2062 PE=3 SV=1	1.7912 71347	0.00057 38	up
Q88WM6	Exodeoxyribonuclease 7 small subunit	Exodeoxyribonuclease 7 small subunit OS=Lactobacillus plantarum (strain ATCC BAA-793 / NCIMB 8826 / WCFS1) OX=220668 GN=xseB PE=3 SV=1	1.9155 26472	0.00028 48	up
F9USD9	lp_3012	NAD-dependent epimerase/dehydratase protein family OS=Lactobacillus plantarum (strain ATCC BAA-793 / NCIMB 8826 / WCFS1) OX=220668 GN=lp_3012 PE=4 SV=1	1.8398 4375	0.00020 43	up
F9UPU1	tagB3	Glycosyl/glycerophosphate transferase, teichoic acid biosynthesis protein B OS=Lactobacillus plantarum (strain ATCC BAA-793 / NCIMB 8826 / WCFS1) OX=220668 GN=tagB3 PE=3 SV=1	1.8962 07585	0.00022 98	up
P37063	Pyruvate oxidase	Pyruvate oxidase OS=Lactobacillus plantarum (strain ATCC BAA-793 / NCIMB 8826 / WCFS1) OX=220668 GN=pox5 PE=1 SV=3	1.8422 60153	0.00019 07	up
F9UPQ0	DUF1722 domain-containing protein	DUF1722 domain-containing protein OS=Lactobacillus plantarum (strain ATCC BAA-793 / NCIMB 8826 / WCFS1) OX=220668 GN=lp_1929 PE=4 SV=1	2.3256 89136	5.93E-0 7	up
F9URG4	lp_2676	Transcription regulator, MerR family OS=Lactobacillus plantarum (strain ATCC BAA-793 / NCIMB 8826 / WCFS1) OX=220668 GN=lp_2676 PE=4 SV=1	1.5542 10433	0.01962	up
F9URU3	lp_2840	Ribosomal protein serine-acetylating enzyme OS=Lactobacillus plantarum (strain ATCC BAA-793 / NCIMB 8826 / WCFS1) OX=220668 GN=lp_2840 PE=4 SV=1	1.7726 65765	0.00072 88	up
Q88SE8	Galactokinase	Galactokinase OS=Lactobacillus plantarum (strain ATCC BAA-793 / NCIMB 8826 / WCFS1) OX=220668 GN=galK PE=3 SV=1	1.9545 45455	0.00184 3	up
F9UMT0	DUF2187 domain-containing protein	Uncharacterized protein OS=Lactobacillus plantarum (strain ATCC BAA-793 / NCIMB 8826 / WCFS1) OX=220668 GN=lp_1123 PE=4 SV=1	2.0638 70968	0.00068 64	up
F9UTE7	lp_3248	Bacteriocin immunity protein OS=Lactobacillus plantarum (strain ATCC BAA-793 / NCIMB 8826 / WCFS1) OX=220668 GN=lp_3248 PE=4 SV=1	2.0148 30508	0.00013 26	up
Q6LWD7	DUF3847 domain-c	Uncharacterized protein OS=Lactobacillus plantarum (strain ATCC BAA-793 / NCIMB 8826 / WCFS1) OX=220668 GN=orf40 PE=4 SV=1	1.8856 38838	0.00125 6	up

		ontaining protein				
	F9UTF7	crtM	Dehydrosqualene synthase OS=Lactobacillus plantarum (strain ATCC BAA-793 / NCIMB 8826 / WCFS1) OX=220668 GN=crtM PE=4 SV=1	2.3185 11584	7.53E-0 8	up
	F9UND2	gtcA1	Teichoic acid glycosylation protein OS=Lactobacillus plantarum (strain ATCC BAA-793 / NCIMB 8826 / WCFS1) OX=220668 GN=gtcA1 PE=3 SV=1	1.6205 1756	0.00747 3	up
	F9UN61	lp_1275	1,2-diacylglycerol 3-glucosyltransferase OS=Lactobacillus plantarum (strain ATCC BAA-793 / NCIMB 8826 / WCFS1) OX=220668 GN=lp_1275 PE=4 SV=1	1.9142 79827	0.00073 96	up
	F9UM31	exoA	Exodeoxyribonuclease III OS=Lactobacillus plantarum (strain ATCC BAA-793 / NCIMB 8826 / WCFS1) OX=220668 GN=exoA PE=3 SV=1	1.7489 7521	0.00207 2	up
	F9UM47	lp_0829	Nitroreductase family protein OS=Lactobacillus plantarum (strain ATCC BAA-793 / NCIMB 8826 / WCFS1) OX=220668 GN=lp_0829 PE=3 SV=1	2.4709 66148	1.84E-0 7	up
	F9UN56	lp_1267	Transcription regulator, MarR family OS=Lactobacillus plantarum (strain ATCC BAA-793 / NCIMB 8826 / WCFS1) OX=220668 GN=lp_1267 PE=4 SV=1	3.8790 40667	0.00018 81	up
	F9URP0	lp_2770	Transcription regulator, biotin repressor family OS=Lactobacillus plantarum (strain ATCC BAA-793 / NCIMB 8826 / WCFS1) OX=220668 GN=lp_2770 PE=4 SV=1	2.3365 81887	0.00040 95	up
	F9UPI8	lp_1856	5-methyltetrahydropteroyltriglutamate-homocysteine methyltransferase (Cobalamine-independent methonine synthase) OS=Lactobacillus plantarum (strain ATCC BAA-793 / NCIMB 8826 / WCFS1) OX=220668 GN=lp_1856 PE=4 SV=1	2.2516 35514	1.99E-0 5	up
	F9UT44	lp_0245	Transcription regulator, GntR family OS=Lactobacillus plantarum (strain ATCC BAA-793 / NCIMB 8826 / WCFS1) OX=220668 GN=lp_0245 PE=4 SV=1	2.3871 12521	1.36E-0 5	up
	F9UTT4	lp_0332	Hypothetical membrane protein OS=Lactobacillus plantarum (strain ATCC BAA-793 / NCIMB 8826 / WCFS1) OX=220668 GN=lp_0332 PE=4 SV=1	2.2205 77872	6.26E-0 5	up
	F9UTB4	lp_3207	PLP-dependent aminotransferase OS=Lactobacillus plantarum (strain ATCC BAA-793 / NCIMB 8826 / WCFS1) OX=220668 GN=lp_3207 PE=4 SV=1	2.6447 54494	2.20E-0 6	up
	F9UT56	pnuC1	Nicotinamide mononucleotide transporter OS=Lactobacillus plantarum (strain ATCC BAA-793 / NCIMB 8826 / WCFS1) OX=220668 GN=pnuC1 PE=3 SV=1	1.9948 66071	0.00143 3	up
	F9UN99	glpQ1	Glycerophosphodiester phosphodiesterase OS=Lactobacillus plantarum (strain ATCC BAA-793 / NCIMB 8826 / WCFS1) OX=220668 GN=glpQ1 PE=4 SV=1	3.1216 98113	4.62E-0 5	up
	F9UTW8	Alpha-gly cerophosp hate oxidase	Alpha-glycerophosphate oxidase OS=Lactobacillus plantarum (strain ATCC BAA-793 / NCIMB 8826 / WCFS1) OX=220668 GN=glpD PE=3 SV=1	2.6875	9.22E-0 5	up
	Q88ZF1	Glycerol kinase 1	Glycerol kinase 1 OS=Lactobacillus plantarum (strain ATCC BAA-793 / NCIMB 8826 / WCFS1) OX=220668 GN=glpK1 PE=3 SV=1	3.0834 14161	6.54E-0 5	up
	F9UPR0	lp_1939	Oxidoreductase, medium chain dehydrogenases/reductase (MDR)/zinc-dependent alcohol dehydrogenase-like family OS=Lactobacillus plantarum (strain ATCC BAA-793 / NCIMB 8826 / WCFS1) OX=220668 GN=lp_1939 PE=4 SV=1	2.6084 54811	0.00066 36	up
	F9UM81	gph1	Phosphohydrolase OS=Lactobacillus plantarum (strain ATCC BAA-793 / NCIMB 8826 / WCFS1) OX=220668 GN=gph1 PE=1 SV=1	1.8321 67832	0.04432	up
	F9UNI1	GTP cyclohydr olase-2	GTP cyclohydrolase-2 OS=Lactobacillus plantarum (strain ATCC BAA-793 / NCIMB 8826 / WCFS1) OX=220668 GN=ribA PE=3 SV=1	1.8155 81395	0.04703	up
**P/N**	F9UL76	Mini-ribo nuclease 3	Mini-ribonuclease 3 OS=Lactobacillus plantarum (strain ATCC BAA-793 / NCIMB 8826 / WCFS1) OX=220668 GN=mrnC PE=3 SV=1	0.5323 26569	0.0315	down
	F9UM73	AP2/ERF domain-c ontaining protein	AP2/ERF domain-containing protein OS=Lactobacillus plantarum (strain ATCC BAA-793 / NCIMB 8826 / WCFS1) OX=220668 GN=lp_0862 PE=4 SV=1	0.6591 87386	0.00054 28	down
	F9UNT7	lp_1557	Transcription regulator, MarR family OS=Lactobacillus plantarum (strain ATCC BAA-793 / NCIMB 8826 / WCFS1) OX=220668 GN=lp_1557 PE=4 SV=1	0.5265 64278	3.72E-0 5	down
	F9UN26	lp_1233	Priming glycosyltransferase,undecaprenyl-phosphate beta-glucosephosphotransferase OS=Lactobacillus plantarum (strain ATCC BAA-793 / NCIMB 8826 / WCFS1) OX=220668 GN=lp_1233 PE=3 SV=1	0.5732 17496	1.90E-0 5	down
	Q88YG1	Uracil-D NA glycosylas	Uracil-DNA glycosylase OS=Lactobacillus plantarum (strain ATCC BAA-793 / NCIMB 8826 / WCFS1) OX=220668 GN=ung PE=3 SV=1	0.6209 1029	0.02028	down

	e				
Q88W97	Holliday junction resolvase RecU	Holliday junction resolvase RecU OS=Lactobacillus plantarum (strain ATCC BAA-793 / NCIMB 8826 / WCFS1) OX=220668 GN=recU PE=3 SV=1	0.6451 6129	5.58E-0 5	down
F9UUF7	lp_3491	Fumarate reductase, flavoprotein subunit OS=Lactobacillus plantarum (strain ATCC BAA-793 / NCIMB 8826 / WCFS1) OX=220668 GN=lp_3491 PE=1 SV=1	0.6017 88171	0.00093 34	down
F9UR15	Triple QxxK/R motif-cont aining protein	Uncharacterized protein OS=Lactobacillus plantarum (strain ATCC BAA-793 / NCIMB 8826 / WCFS1) OX=220668 GN=lp_2483 PE=4 SV=1	1.7020 96031	0.00301 4	up
F9URU8	lp_2845	Extracellular transglycosylase, with LysM peptidoglycan binding domain OS=Lactobacillus plantarum (strain ATCC BAA-793 / NCIMB 8826 / WCFS1) OX=220668 GN=lp_2845 PE=4 SV=1	1.5113 13505	0.00443	up
F9URD4	lp_2636	Extracellular protein OS=Lactobacillus plantarum (strain ATCC BAA-793 / NCIMB 8826 / WCFS1) OX=220668 GN=lp_2636 PE=4 SV=1	1.5274 56647	0.00401	up
F9URP2	lp_2772	Transcription regulator, TetR family OS=Lactobacillus plantarum (strain ATCC BAA-793 / NCIMB 8826 / WCFS1) OX=220668 GN=lp_2772 PE=4 SV=1	1.6183 39153	0.03796	up
F9UUB9	lp_3444	Transcription regulator, Crp family OS=Lactobacillus plantarum (strain ATCC BAA-793 / NCIMB 8826 / WCFS1) OX=220668 GN=lp_3444 PE=4 SV=1	1.5008 01399	0.00028 37	up
F9UQA9	ktrA	Potassium uptake protein OS=Lactobacillus plantarum (strain ATCC BAA-793 / NCIMB 8826 / WCFS1) OX=220668 GN=ktrA PE=4 SV=1	1.8026 31579	0.00244 1	up
F9UP60	lp_1697	Adherence protein, chitin-binding domain OS=Lactobacillus plantarum (strain ATCC BAA-793 / NCIMB 8826 / WCFS1) OX=220668 GN=lp_1697 PE=4 SV=1	1.6979 33227	0.00069 59	up
F9USB9	zmp3	Extracellular zinc metalloproteinase, M10 family OS=Lactobacillus plantarum (strain ATCC BAA-793 / NCIMB 8826 / WCFS1) OX=220668 GN=zmp3 PE=4 SV=1	1.5381 1592	0.00056 9	up
F9USK7	lp_3093	Lysozyme/muramidase, glycoside hydrolase family 25 OS=Lactobacillus plantarum (strain ATCC BAA-793 / NCIMB 8826 / WCFS1) OX=220668 GN=lp_3093 PE=3 SV=1	1.5195 1952	0.00506 1	up
F9UTM4	Phage protein	Uncharacterized protein OS=Lactobacillus plantarum (strain ATCC BAA-793 / NCIMB 8826 / WCFS1) OX=220668 GN=lp_3351 PE=4 SV=1	1.6220 86657	0.00782 2	up
Q88VM8	D-alanyl carrier protein 1	D-alanyl carrier protein 1 OS=Lactobacillus plantarum (strain ATCC BAA-793 / NCIMB 8826 / WCFS1) OX=220668 GN=dltC1 PE=3 SV=1	1.5597 73458	0.00511 4	up
F9US19	lp_2934	Lipoprotein OS=Lactobacillus plantarum (strain ATCC BAA-793 / NCIMB 8826 / WCFS1) OX=220668 GN=lp_2934 PE=4 SV=1	1.5242 42424	0.00022 93	up
F9UQU1	lp_2401	Prophage P2a protein 56, lysin OS=Lactobacillus plantarum (strain ATCC BAA-793 / NCIMB 8826 / WCFS1) OX=220668 GN=lp_2401 PE=3 SV=1	1.7029 28186	0.02193	up
F9UTJ1	DUF2187 domain-c ontaining protein	Uncharacterized protein OS=Lactobacillus plantarum (strain ATCC BAA-793 / NCIMB 8826 / WCFS1) OX=220668 GN=lp_3305 PE=4 SV=1	1.5086 56351	0.00224 2	up
P96349	Cold shock protein 2	Cold shock protein 2 OS=Lactobacillus plantarum (strain ATCC BAA-793 / NCIMB 8826 / WCFS1) OX=220668 GN=cspL PE=2 SV=1	1.6500 73445	0.00106 4	up
Q88S84	L-arabino se isomerase	L-arabinose isomerase OS=Lactobacillus plantarum (strain ATCC BAA-793 / NCIMB 8826 / WCFS1) OX=220668 GN=araA PE=3 SV=1	1.5191 79584	0.00049 03	up
F9UKZ2	LemA family protein	Uncharacterized protein OS=Lactobacillus plantarum (strain ATCC BAA-793 / NCIMB 8826 / WCFS1) OX=220668 GN=lp_0515 PE=3 SV=1	1.7986 88641	7.17E-0 6	up
F9USH2	lp_3050	Extracellular transglycosylase, membrane-bound OS=Lactobacillus plantarum (strain ATCC BAA-793 / NCIMB 8826 / WCFS1) OX=220668 GN=lp_3050 PE=4 SV=1	1.5713 35399	0.02953	up
F9USS1	lp_0092	Oligopeptide ABC transporter,lipoprotein-binding protein OS=Lactobacillus plantarum (strain ATCC BAA-793 / NCIMB 8826 / WCFS1) OX=220668 GN=lp_0092 PE=4 SV=1	1.5029 44453	0.01009	up
F9USD6	pts23B	PTS system, cellobiose-specific EIIB component OS=Lactobacillus plantarum (strain ATCC BAA-793 / NCIMB 8826 / WCFS1) OX=220668 GN=pts23B PE=4 SV=1	1.5776 75489	0.00010 6	up
F9URS4	lp_2812	Extracellular protein, membrane-anchored OS=Lactobacillus plantarum (strain ATCC BAA-793 / NCIMB 8826 / WCFS1) OX=220668 GN=lp_2812 PE=4 SV=1	1.5027 15655	0.00236 3	up

F9UT86	lp_3173	Cell surface protein, membrane-anchored OS=Lactobacillus plantarum (strain ATCC BAA-793 / NCIMB 8826 / WCFS1) OX=220668 GN=lp_3173 PE=4 SV=1	1.9110779	0.000325	up
F9ULR9	lp_0681	Prophage P1 protein 58, lysin OS=Lactobacillus plantarum (strain ATCC BAA-793 / NCIMB 8826 / WCFS1) OX=220668 GN=lp_0681 PE=3 SV=1	2.325481799	0.0002539	up
F9UUC4	lp_3451	Cell surface protein, CscA/DUF916 family OS=Lactobacillus plantarum (strain ATCC BAA-793 / NCIMB 8826 / WCFS1) OX=220668 GN=lp_3451 PE=4 SV=1	4.659132337	0.009096	up
Q6LWH7	repA	Copy-number control protein OS=Lactobacillus plantarum (strain ATCC BAA-793 / NCIMB 8826 / WCFS1) OX=220668 GN=repA PE=4 SV=1	2.091072499	0.001548	up
F9UPA0	lp_1746	D-methionine ABC transporter, substrate binding protein OS=Lactobacillus plantarum (strain ATCC BAA-793 / NCIMB 8826 / WCFS1) OX=220668 GN=lp_1746 PE=3 SV=1	1.594097808	0.005821	up
F9UTK3	lp_3324	Glycine betaine/carnitine/choline transport protein OS=Lactobacillus plantarum (strain ATCC BAA-793 / NCIMB 8826 / WCFS1) OX=220668 GN=lp_3324 PE=3 SV=1	1.670227671	0.001566	up
F9URP0	lp_2770	Transcription regulator, biotin repressor family OS=Lactobacillus plantarum (strain ATCC BAA-793 / NCIMB 8826 / WCFS1) OX=220668 GN=lp_2770 PE=4 SV=1	1.821382249	0.001172	up
F9UQU0	lp_2400	Prophage P2a protein 57 OS=Lactobacillus plantarum (strain ATCC BAA-793 / NCIMB 8826 / WCFS1) OX=220668 GN=lp_2400 PE=4 SV=1	1.595071025	0.00635	up
Q88YX4	50S ribosomal protein L33	50S ribosomal protein L33 OS=Lactobacillus plantarum (strain ATCC BAA-793 / NCIMB 8826 / WCFS1) OX=220668 GN=rpmG PE=3 SV=1	1.879770992	0.004058	up
F9UM52	spx1	RNA polymerase (RNAP)-binding regulatory protein, arsenate reductase (ArsC) family, Spx subfamily OS=Lactobacillus plantarum (strain ATCC BAA-793 / NCIMB 8826 / WCFS1) OX=220668 GN=spx1 PE=3 SV=1	1.50282392	0.004013	up
F9UMI2	cspC	Cold shock protein CspC OS=Lactobacillus plantarum (strain ATCC BAA-793 / NCIMB 8826 / WCFS1) OX=220668 GN=cspC PE=4 SV=1	1.812382739	0.001571	up
F9UMC4	lp_0925	Acyltransferase OS=Lactobacillus plantarum (strain ATCC BAA-793 / NCIMB 8826 / WCFS1) OX=220668 GN=lp_0925 PE=4 SV=1	1.591502699	0.02425	up
F9UUF2	rafP	Putative PTS system EIIA component OS=Lactobacillus plantarum (strain ATCC BAA-793 / NCIMB 8826 / WCFS1) OX=220668 GN=rafP PE=3 SV=1	2.232608696	6.77E-05	up
Q88SE8	Galactokinase	Galactokinase OS=Lactobacillus plantarum (strain ATCC BAA-793 / NCIMB 8826 / WCFS1) OX=220668 GN=galK PE=3 SV=1	1.779198796	0.002226	up
P37063	Pyruvate oxidase	Pyruvate oxidase OS=Lactobacillus plantarum (strain ATCC BAA-793 / NCIMB 8826 / WCFS1) OX=220668 GN=pox5 PE=1 SV=3	1.844793713	0.0002031	up
F9UKY3	L-serine dehydratase	L-serine dehydratase OS=Lactobacillus plantarum (strain ATCC BAA-793 / NCIMB 8826 / WCFS1) OX=220668 GN=sdhB PE=3 SV=1	3.155127082	0.000273	up
F9UTJ6	Pyruvate formate-lyase-activating enzyme	Pyruvate formate-lyase-activating enzyme OS=Lactobacillus plantarum (strain ATCC BAA-793 / NCIMB 8826 / WCFS1) OX=220668 GN=pflA PE=3 SV=1	1.743753632	0.002787	up
F9UTQ7	lp_0302	Extracellular transglycosylase OS=Lactobacillus plantarum (strain ATCC BAA-793 / NCIMB 8826 / WCFS1) OX=220668 GN=lp_0302 PE=4 SV=1	1.711676705	0.02767	up
F9UQ59	Glycogen biosynthesis protein GlgD	Uncharacterized protein OS=Lactobacillus plantarum (strain ATCC BAA-793 / NCIMB 8826 / WCFS1) OX=220668 GN=lp_2112 PE=4 SV=1	2.384223301	0.0001971	up
F9UUC6	lp_3453	Cell surface protein, CscB family OS=Lactobacillus plantarum (strain ATCC BAA-793 / NCIMB 8826 / WCFS1) OX=220668 GN=lp_3453 PE=4 SV=1	2.185779817	4.11E-05	up
F9UM76	Transcriptional regulator	Uncharacterized protein OS=Lactobacillus plantarum (strain ATCC BAA-793 / NCIMB 8826 / WCFS1) OX=220668 GN=lp_0865 PE=4 SV=1	2.240983998	0.0002903	up
F9URW4	Signal peptidase I	Signal peptidase I OS=Lactobacillus plantarum (strain ATCC BAA-793 / NCIMB 8826 / WCFS1) OX=220668 GN=sip2 PE=3 SV=1	1.924565217	0.006251	up
F9UPP9	spx2	RNA polymerase (RNAP)-binding regulatory protein, arsenate reductase (ArsC) family, Spx subfamily OS=Lactobacillus plantarum (strain ATCC BAA-793 / NCIMB 8826 / WCFS1) OX=220668 GN=spx2 PE=3 SV=1	3.559495397	7.07E-05	up

F9URE0	pts19A	PTS system,N-acetylglucosamine/galactosamine-specific EIIA component OS=Lactobacillus plantarum (strain ATCC BAA-793 / NCIMB 8826 / WCFS1) OX=220668 GN=pts19A PE=4 SV=1	2.7620 3966	4.01E-0 5	up
Q88WK6	50S ribosomal protein L28	50S ribosomal protein L28 OS=Lactobacillus plantarum (strain ATCC BAA-793 / NCIMB 8826 / WCFS1) OX=220668 GN=rpmB PE=3 SV=1	2.2296 0199	0.00231 5	up
F9UN99	glpQ1	Glycerophosphodiester phosphodiesterase OS=Lactobacillus plantarum (strain ATCC BAA-793 / NCIMB 8826 / WCFS1) OX=220668 GN=glpQ1 PE=4 SV=1	3.3012 96974	7.00E-0 5	up
F9ULR3	lp_0675	Prophage P1 protein 52, endolysin OS=Lactobacillus plantarum (strain ATCC BAA-793 / NCIMB 8826 / WCFS1) OX=220668 GN=lp_0675 PE=3 SV=1	3.4300 03457	0.00111 5	up
F9UMT0	DUF2187 domain-c ontaining protein	Uncharacterized protein OS=Lactobacillus plantarum (strain ATCC BAA-793 / NCIMB 8826 / WCFS1) OX=220668 GN=lp_1123 PE=4 SV=1	3.3709 16754	0.00047 35	up
F9UTW8	Alpha-gly cerophosp hate oxidase	Alpha-glycerophosphate oxidase OS=Lactobacillus plantarum (strain ATCC BAA-793 / NCIMB 8826 / WCFS1) OX=220668 GN=glpD PE=3 SV=1	3.2253 66519	5.82E-0 5	up
Q88ZF1	Glycerol kinase 1	Glycerol kinase 1 OS=Lactobacillus plantarum (strain ATCC BAA-793 / NCIMB 8826 / WCFS1) OX=220668 GN=glpK1 PE=3 SV=1	4.0531 23672	3.98E-0 5	up
F9UNI1	GTP cyclohydr olase-2	GTP cyclohydrolase-2 OS=Lactobacillus plantarum (strain ATCC BAA-793 / NCIMB 8826 / WCFS1) OX=220668 GN=ribA PE=3 SV=1	2.1214 67391	0.0307	up
F9UTT5	NfeD domain-c ontaining protein	Uncharacterized protein OS=Lactobacillus plantarum (strain ATCC BAA-793 / NCIMB 8826 / WCFS1) OX=220668 GN=lp_0333 PE=4 SV=1	5.3494 71341	0.00011 07	up
